# Stressomic: A wearable microfluidic biosensor for dynamic profiling of multiple stress hormones in sweat

**DOI:** 10.1126/sciadv.adx6491

**Published:** 2025-08-06

**Authors:** Jiaobing Tu, Jeonghee Yeom, Joshua Chaj Ulloa, Samuel A. Solomon, Jihong Min, Wenzheng Heng, Gwangmook Kim, Jadelynn Dao, Rohan Vemu, Marion Pang, Canran Wang, Dong-Hwan Kim, Wei Gao

**Affiliations:** ^1^ Andrew and Peggy Cherng Department of Medical Engineering, Division of Engineering and Applied Science, California Institute of Technology, Pasadena, CA, USA.; ^2^ School of Chemical Engineering, Sungkyunkwan University, Suwon, Republic of Korea.

## Abstract

Managing stress is essential for mental and physical health, yet current methods rely on subjective self-assessments or indirect physiological measurements, often lacking accuracy. Existing wearable sensors primarily target a single stress hormone, cortisol, using single-point measurements that fail to capture real-time changes and distinguish between acute and chronic stress. To address this, we present Stressomic, a wearable multiplexed microfluidic biosensor for noninvasive monitoring of cortisol, epinephrine, and norepinephrine in sweat. Stressomic integrates iontophoresis-driven sweat extraction with bursting valve-regulated microfluidic channels for continuous sampling and analysis. Gold nanodendrite–decorated laser-engraved graphene electrodes achieve picomolar-level sensitivity, enabling simultaneous detection of multiple stress hormones. Electrochemical assays and human studies demonstrate that Stressomic reliably tracks hormone fluctuations in response to physical, psychological, and pharmacological stressors. Distinct temporal patterns reveal the dynamic interplay between the hypothalamic-pituitary-adrenal axis and the sympathetic nervous system. This platform enables continuous, multiplexed stress profiling, offering opportunities for early detection of maladaptive responses, personalized stress management, and deeper insights into stress biology.

## Teaser

A wearable sensor enables dynamic and noninvasive monitoring of multiple stress hormones to personalize stress management.

## INTRODUCTION

Stress is mankind’s fundamental physiological and psychological response to perceived threats or challenges, triggering hormonal and neural cascades that mobilize energy while suppressing nonessential functions. This adaptive mechanism, categorized into eustress (positive stress) and distress (negative stress), plays a dual role: enhancing performance and focus in the former while impairing cognitive and emotional well-being in the latter (*1*–*3*). Prolonged or chronic distress is associated with severe health consequences, including mental health disorders, weakened immune functions, and cardiovascular disease (*4*). At a broader scale, stress-related conditions impose substantial burdens on public health systems, with mental health encounters ranked as the second most common reason for primary care visits. This underscores the pressing need for improved approaches to assess and manage stress-related health issues at the point of care (*5*).

**Fig. 1.**
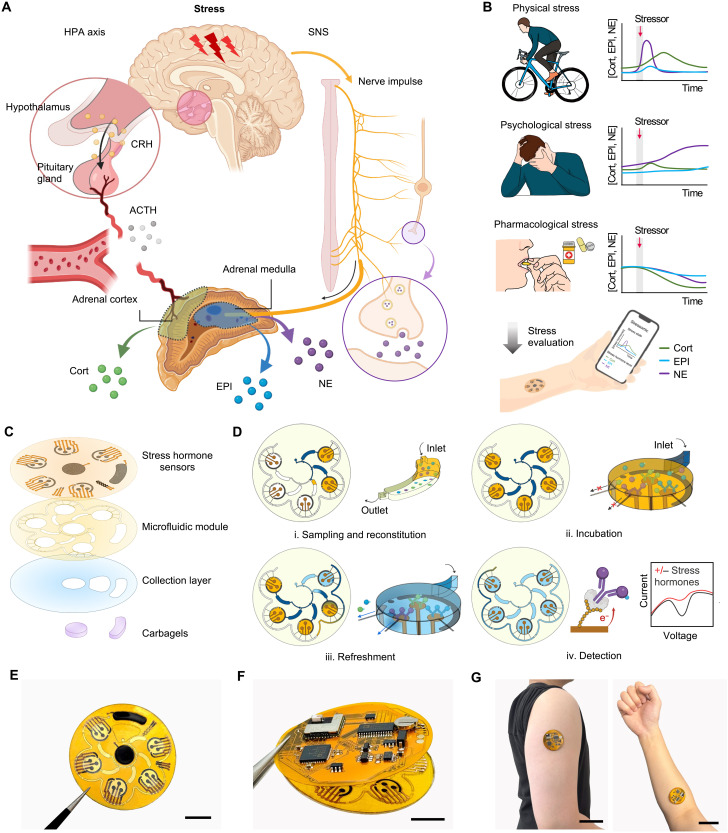
Overview of Stressomic, a multiplexed wearable stress hormone monitoring system. (**A**) Schematic illustration of the body’s stress response pathways. The HPA axis triggers cortisol (Cort) release via corticotropin-releasing hormone (CRH) and adrenocorticotropic hormone (ACTH), while the SNS stimulates the adrenal medulla to release epinephrine (EPI) and norepinephrine (NE) into the bloodstream and secrete NE at nerve terminals to regulate local tissue responses. (**B**) Representative profiles of Cort, EPI, and NE levels under physical stress (HIIT), emotional stress (IAPS), and after stress modulation (supplementation). These data illustrate example trends rather than continuous hormone dynamics. (**C**) Design of Stressomic, consisting of three hormone biosensors, a microfluidic module for automated sequential sweat sampling, reconstitution, and reagent delivery, a collection layer that facilitates sweat sampling and accumulation, and a pair of carbachol-loaded hydrogels for on-demand sweat induction. (**D**) Workflow of the microfluidic system for stress hormone detection: (i) Sweat fills reagent and detection chambers, reconstituting analytes in buffer with redox-labeled competitors; (ii) Analytes incubate and compete for binding at electrodes in detection chambers; (iii) Unbound molecules are flushed to preserve sensor accuracy during automated refresh cycles; (iv) Electrochemical detection quantifies hormones by measuring redox signals, enabling sensitive, real-time monitoring. (**E** to **G**) Photos of Stressomic including the microfluidic sensor patch (E), fully integrated wireless system (F), and a system worn on the upper arm and forearm (G). Scale bars, 1 cm (E and F) and 5 cm (G).

The multifaceted nature of stress necessitates comprehensive monitoring strategies. Current assessments primarily rely on subjective self-reported questionnaires, such as the Perceived Stress Scale (*6*). While valuable for long-term mental health tracking, these methods fall short in capturing short-term fluctuations or providing quantitative insights into physiological changes. Wearable devices capable of measuring physiological parameters, such as heart rate variability, offer real-time insights into the body’s responses to stress (*7*–*9*) but lack specificity and fail to distinguish between acute and chronic stress responses. Moreover, they cannot differentiate between eustress and distress, limiting their use for comprehensive stress profiling. To achieve a more precise and granular understanding of stress dynamics, monitoring primary stress hormones—cortisol (Cort), epinephrine (EPI), and norepinephrine (NE)—is critical (*10*). These hormones play distinct yet interconnected roles in the regulation of the body’s two primary stress response systems: the hypothalamic-pituitary-adrenal (HPA) axis and the sympathetic nervous system (SNS) (Fig. 1A) (*10*, *11*). Cort, a key regulator of energy metabolism, reflects prolonged stress and HPA-axis activity, while EPI and NE are rapidly released by the SNS during acute stress, driving the immediate “fight-or-flight” response.

Sweat analysis has emerged as a noninvasive and continuous method to monitor stress-related biomarkers, offering high temporal resolution of stress dynamics without the need for invasive, episodic, and stress-inducing blood sampling (*12*–*16*). Recent advances in wearable sweat sensors have demonstrated the potential of hormone tracking in near real time (*17*–*22*). However, current devices are limited to detecting Cort only due to its relatively high nanomolar concentrations and typically provide only single-point measurements. This singular focus on cortisol overlooks acute stress events, where catecholamines—EPI and NE—play a dominant role. Beyond acute stress, catecholamines are integral to emotional regulation and mood, reinforcing the need for their simultaneous monitoring alongside Cort (*23*, *24*). Capturing the dynamic interplay between these hormones enables a holistic evaluation of both immediate stress responses and the cumulative impact of chronic stress, known as allostatic load. Despite its potential, in situ detection of EPI and NE in sweat remains unexplored, primarily because of their low physiological concentrations (picomolar levels), their short half-lives, and the inherent complexities of multiplexed hormone analysis.

To address these gaps, we present Stressomic, a multiplexed wearable microfluidic biosensor designed to capture real-time dynamic profiles of multiple stress hormones in sweat (Fig. 1B). The device features an autonomous iontophoresis module loaded with carbachol hydrogels, enabling on-demand and controlled sweat extraction, seamlessly integrated with a skin-interfaced microfluidic module that facilitates sequential sweat sampling, on-skin reagent delivery, and multiplexed stress hormone analysis (Fig. 1, C and D). The platform uses gold nanodendrite–decorated laser-engraved graphene (AuND-LEG) electrodes, providing highly sensitive electrochemical detection of each stress hormone. Using methylene blue (MB)–labeled antigens, competitive binding at the AuND-LEG surface generates a direct reduction peak current inversely proportional to hormone concentration. The microfluidic design incorporates a series of capillary burst valves (CBVs) to regulate sweat sampling, routing, and reagent refresh cycles, enabling high temporal resolution and continuous profiling of stress biomarkers (Fig. 1D). The mechanically flexible wearable system conforms seamlessly to the skin, enabling continuous, wireless data collection across diverse real-life scenarios (Fig. 1, E to G).

In this study, we demonstrate the presence of EPI and NE in human sweat and uncover differential dynamic profiles of all three stress hormones in response to physical, psychological, and pharmacological modulators. This multiplexed sensing approach offers critical insights into the interplay between the SNS and HPA axis, enabling differentiation between acute and chronic stress responses and quantifying stress response. Stressomic holds the potential to transform stress assessment, enabling personalized interventions and improving our understanding of stress physiology.

## RESULTS

### Electrochemical characterization of the multiplexed stress immunosensor

**Fig. 2.**
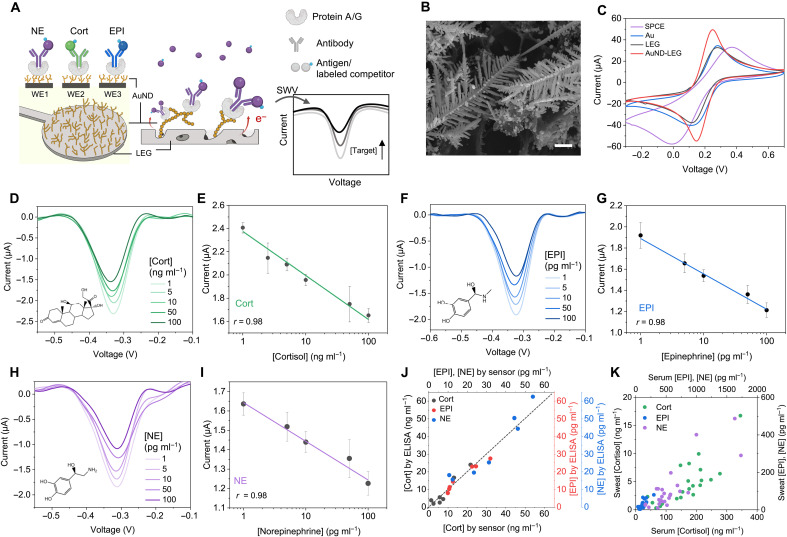
Electrochemical characterization of the multiplexed stress hormone immunosensor. (**A**) Schematic of the sensor design and the electrochemical detection of stress hormone Cort, EPI, and NE in human sweat. (**B**) Scanning electron microscope image of the AuNDs deposited on an LEG electrode. Scale bar, 1 μm. (**C**) Cyclic voltammetry (CV) scans comparing screen-printed carbon electrode (SPCE), Au, LEG, and AuND-LEG electrodes in a solution containing 5 mM [Fe(CN)_6_]^3−^ and 0.1 M KCl. (**D** to **I**) Square wave voltammetry (SWV) voltammograms and corresponding calibration curves of Cort (D and E), EPI (F and G), and NE (H and I) sensors in 1× phosphate-buffered saline (PBS) (pH 7.4). Error bars represent the SD from three sensors. (**J**) Validation of the stress immunosensors in human sweat samples (*n* = 21 biological replicates) with enzyme-linked immunosorbent assay (ELISA). (**K**) Correlation between serum and sweat stress hormone levels. Sweat stress hormone concentrations were determined using the stress immunosensors, while serum hormone levels were determined by ELISA (*n* = 74 biological replicates).

The preparation process of the multiplexed stress immunosensor, designed to detect three stress hormones—Cort, EPI, and NE—in sweat, is illustrated in Fig. 2A. Each working electrode (WE) is modified with protein A or protein G to facilitate the specific capture antibody (cAb) immobilization. During sensing, competitive binding occurs between target hormones and redox probe–labeled antigens at the cAb sites. The generated reduction current inversely correlates with hormone concentration in sweat.

LEG serves as the foundational material for the immunosensor due to its large surface area and rich electrocatalytic properties. To enhance sensitivity, we electrodeposited Au nanodendrites onto the porous graphene scaffold, further improving electron transfer. This overpotential-driven deposition uses hydrogen bubbles as templates to form nanodendritic structures, substantially improving sensor performance (Fig. 2, B and C, and figs. S1 to S3) (*25*). Functionalization of AuND-LEG is achieved by conjugating protein A or protein G to the AuNDs via thiol bonding (*26*), enabling oriented antibody attachment. This ensures optimal antigen binding and reduces variability (fig. S4). Compared to gold nanoparticle (AuNP)–modified LEG electrodes, the AuND-LEG electrodes provide a greater surface area, resulting in higher currents after surface modification (fig. S5).

Differential pulse voltammetry (DPV) and electrochemical impedance spectroscopy (EIS) were used to verify sensor functionalization after each modification step (fig. S6). The AuND deposition increased the DPV peak height and lowered the EIS resistance, indicating enhanced electron transfer. In contrast, subsequent protein G functionalization and unmodified Au surface blocking with mercaptohexanol enhanced its insulating property, as indicated by a reduced DPV peak height and increasing EIS resistance, confirming successful modification.

Hormone detection is based on the competitive binding between target stress hormones and in-house synthesized MB-labeled antigens. These antigens were synthesized by reacting *N*-hydroxysuccinimide (NHS) ester derivatives of the stress hormone and MB with cationized bovine serum albumin (BSA), forming MB-labeled hapten-carrier conjugates via stable amide bonds (fig. S7). To prepare EPI-NHS ester and NE-NHS ester, we electrolyzed catecholamines to yield bright orange *o*-quinone intermediates with reactive *o*-hydroxyl moieties (*27*). This process is carefully monitored and optimized using ultraviolet-visible (UV-vis) absorbance spectroscopy (fig. S8). These intermediates are subsequently modified by direct ring conjugation with a short HS–polyethylene glycol (PEG)–COOH linker. The carboxylated forms of EPI and NE are further activated using 1-ethyl-3-(3-dimethylaminopropyl)carbodiimide (EDC)–NHS chemistry, facilitating the conversion of carboxylic groups into NHS esters (fig. S7). Cationized BSA serves as the carrier protein, providing high antigen and redox probe loading capacity and enhancing the sensitivity and dynamic range of the competitive binding assay.

The electrochemical performance of the sensors was evaluated using square wave voltammetry (SWV) (Fig. 2, D to I). The competitive binding assay results in an MB reduction peak inversely proportional to hormone concentrations. The sensors exhibit log-linear responses across physiologically relevant target ranges in sweat (Cort, 0 to 100 ng ml^−1^; EPI and NE, 0 to 100 pg ml^−1^). Reproducibility tests show consistent sensor performance across multiple batches (fig. S9). The sensors achieved picomolar sensitivity for EPI and NE, maintaining specificity even in the presence of interferents at 1000-fold higher concentrations, despite the structural similarity of catecholamines (fig. S10).

The sensor accuracy was validated against the laboratory standard enzyme-linked immunosorbent assay (ELISA) (Fig. 2J). Strong correlations between hormone levels in sweat and serum were identified, supporting a passive diffusion mechanism (Fig. 2K and fig. S11). Differences in dilution may result from molecular size, diffusivity, and interactions with local carrier proteins or enzymatic activity. Systematic studies evaluated the effects of incubation volume, time, pH, ionic strength, and temperature (figs. S12 and S13). The sensors exhibited stable current response with sample volumes >5 μl at 10-min incubation times (fig. S12). The sensors showed high selectivity against potential interferents in sweat and exhibited minimal pH and ionic strength effects near physiological levels due to the buffering capacity of salt-enriched MB-labeled competitors and negligible current drift at physiological temperatures, ensuring reliable on-body performance with reduced signal fluctuations from interpersonal sweat variations (fig. S13). The sensors achieved low detection limits of 2.70 ng ml^−1^ for Cort, 2.73 pg ml^−1^ for EPI, and 9.14 pg ml^−1^ for NE, offering improved sensitivity compared to most existing electrochemical and sweat-based sensors, which typically detect in the higher picomolar to nanomolar range (table S1). The demonstrated detection ranges encompass typical concentrations observed in sweat during acute stress responses, supporting the use of the platform for real-time neuroendocrine monitoring.

### Multiplexed microfluidic patch for capturing dynamic stress hormone profiles

We previously demonstrated the feasibility of predepositing labeled electrochemical reagents in a reagent reservoir for in situ protein detection (*28*); however, this approach was limited to single-use applications, restricting its potential for continuous hormone monitoring essential for stress response investigations. Real-time immunosensing of sweat hormones presents several major challenges. First, the need for labeling reagents and washing steps complicates the detection process, particularly for low-concentration targets requiring extended incubation time for adequate diffusion and binding. This creates a mismatch, as hormone fluctuations often occur on much shorter timescales than required incubation periods. In addition, the slow dissociation rates (<10^−5^ s^−1^) of high-affinity receptors further hinder alignment with the dynamic and rapidly shifting hormone concentrations in sweat.

**Fig. 3.**
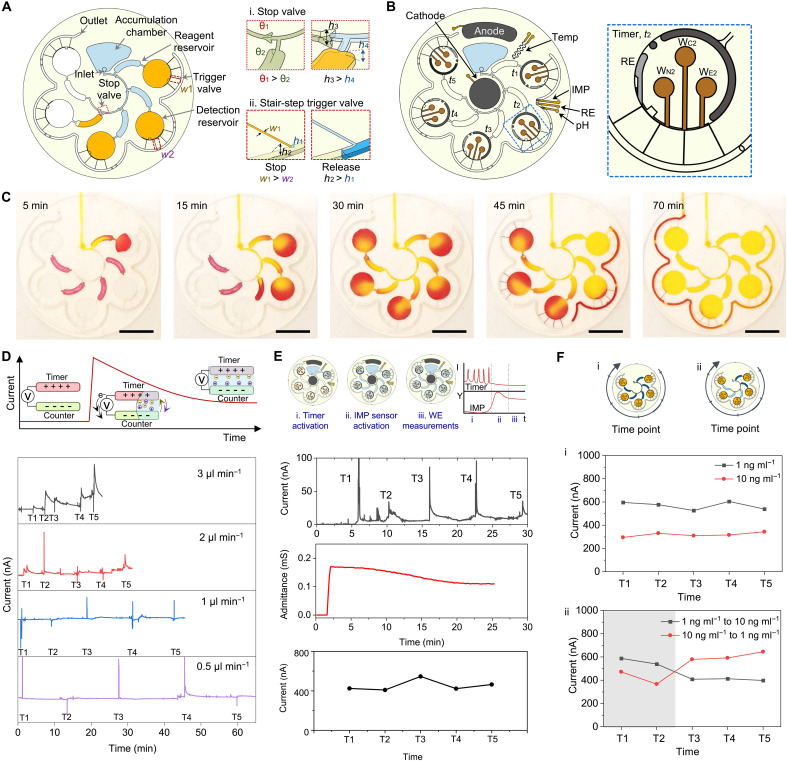
Design and characterization of the multiplexed microfluidic stress hormone sampling system. (**A**) Schematic of the microfluidic system, showing the sweat accumulation chamber, reagent and detection reservoirs, and valve mechanisms. The stop valve (i) and stair-step trigger valve (ii) regulate fluid flow through capillary burst pressure differences, precisely timing reagent release by varying channel dimensions and geometries. (**B**) Electrochemical sensing platform layout with cathode, anode, timer, integrated pH, ionic strength (IMP), and temperature (temp) sensors. Inset: timer structure and WEs for NE (W_N_), Cort (W_C_), EPI (W_E_), reference electrode (RE), and counter electrode (CE) in the second detection reservoir. (**C**) Optical images showing fluid movement, mixing with predeposited competitors (red dye) at different time intervals, and visualizing flow dynamics and reagent refreshment. Scale bars, 1 cm. (**D**) Timer electrode readouts. Top: timer electrode working principle. Bottom: amperometric profiles at different flow rates. (**E**) Workflow of sensing stages: (i) timer activation, (ii) IMP sensor activation, and (iii) WE measurements. Representative current and impedance signals at 1.5 μl min^−1^ for detecting Cort at 1 ng ml^−1^. (**F**) Sensitivity and switching dynamics: (i) current readouts for Cort at 1 and 10 ng ml^−1^; (ii) dynamic detection of Cort concentration shifts (10 to 1 ng ml^−1^ and vice versa) at 1.5 μl min^−1^.

To address these limitations, we designed a microfluidic patch incorporating CBVs to regulate fluid flow and retention (Fig. 3A). CBVs leverage liquid-air interfaces and microchannel geometries to create pressure barriers, enabling sequential fluid movement (*29*). Fabricated using laser-patterned medical tapes and polyethylene terephthalate (PET) film, this method provides scalability and flexibility over traditional silicon-based microfabrication (fig. S14). To stop premature fluid flow and enhance detection sensitivity, we incorporated stop valves with hydrophobic ceilings to direct fluid into reagent reservoirs before progressing downstream [Fig. 3A(i) and fig. S15A]. Stair-step trigger valves at the outlets of the detection chambers further regulate movement by pinning the liquid meniscus in narrow channels that expand abruptly [Fig. 3A(ii) and fig. S15, B and C]. This creates a burst pressure higher than that of the stop valves, preventing flow until the capillary pressure surpasses a defined threshold, thereby prolonging analyte incubation for improved sensitivity before sequential fluid release. A burst pressure gradient was introduced by designing the first chamber with a wider channel (*w*_1_ = 180 μm versus *w*_2_ = 110 μm), enabling preferential initial release as the larger channel reduces the burst pressure. Fluid then progresses through the petal-shaped outlet channel, interacting with pinned liquid meniscus at successive trigger valves, altering the liquid-air interface, and initiating sequential chamber release.

The patch integrates five detection chambers to capture dynamic stressor events, with each chamber comprising a reagent reservoir for labeled antigen delivery, a dedicated detection zone for sweat biomarker incubation, and in situ detection. Each chamber is equipped with dedicated WEs for stress hormones, along with a counter electrode, a reference electrode (RE), and a timer electrode to record the entry time of sweat (Fig. 3B). Each detection chamber functions as a self-contained immunoassay unit, where sequential sample and reagent delivery enables sufficient incubation for diffusion-limited binding. The sensor patch also incorporates ionic strength, pH, and temperature sensors for real-time calibration to enhance measurement accuracy. The WEs for each stress hormone were systematically evaluated to determine optimal performance, resulting in the selection of WE1 for NE, WE2 for Cort, and WE3 for EPI (figs. S16 and S17). Hereafter, the WEs are denoted as W*_Xi_*, where *X* represents the target hormone (N for NE, C for Cort, and E for EPI) and *i* denotes the chamber number (1 to 5) where the electrode is located.

The sequential filling behavior of the microfluidic module was characterized using flow tests with a two-dye system to simulate reagent incubation and release, where red and yellow dyes represent the labeling reagents and simulated sweat, respectively (Fig. 3C and movie S1). At a constant flow rate of 1.5 μl min^−1^, the dye sequentially filled each chamber at 6-min intervals, demonstrating precise fluid control and timed progression (movie S1). Once a chamber is filled, it is isolated from further flow by a CBV, allowing independent incubation and signal development without interference from subsequent samples. This architecture decouples the sampling time (when sweat enters the chamber) from the detection time (when signal is measured), enabling time-stamped snapshots of hormone levels every 6 min, even though signal readout may occur later. This is especially critical for capturing short-timescale hormone fluctuations while accommodating the slower kinetics of high-affinity immunoassays. During operation, the red dye remained in the detection chambers for prolonged incubation. While some upstream mixing may occur, the intermediate chamber serves to buffer and regulate flow, preventing burst flushing into downstream channels. Nonetheless, the number of chambers can be expanded in future designs to extend the monitoring window and capture longer-term hormonal fluctuations. Once all chambers were filled, sequential release was initiated from the first chamber (Fig. 3C). The release and refresh cycles were consistent for each chamber, with the outlet-channel dead volume carefully matched to the combined reagent reservoir and detection chamber volumes. Unlike conventional antibody-based assays that provide only static snapshots of analyte levels, our microfluidic platform enables time-resolved sampling through regulated sequential chamber filling and release.

The timer electrodes integrated in the multiplexed system play a critical role in addressing interpersonal variations in sweat rates. They provide a temporal reference for sweat entry into each chamber, ensuring that hormone measurements are accurately correlated with specific time points to generate precise dynamic stress hormone profiles. Chronoamperometry was performed on the timer electrodes, connected in parallel across all chambers, revealing a sharp current spike upon solution entry, followed by a gradual decay to a steady level (Fig. 3D). This response is attributed to charge redistribution upon circuit closure, prompting rapid electron and ion movement. The electrodes function as capacitors, rapidly charging or discharging until equilibrium is reached, resulting in the initial surge followed by stabilization as the current aligns with the applied voltage.

To validate system performance, we tested physiologically relevant flow rates (0.5 to 3 μl min^−1^) typically observed in iontophoresis-based sweat induction (*28*, *30*) and confirmed consistent time intervals across all chambers (Fig. 3D). Calibration data for the pH, ionic strength, and temperature sensors are provided in fig. S18. We also evaluated the mechanical robustness of the system under physical deformation. The multiplexed sensors exhibited minimal signal variation under a relatively small bending radius of 1.5 cm (fig. S19), supporting its practical applicability for continuous, on-body operation of Stressomic in real-world settings. In addition, we evaluated the performance of all three sensors under various matrix conditions (pH, ionic strength, and temperature) to ensure robust on-body operation (fig. S20). Given the minimal signal changes within the physiological skin temperature range, a multivariate polynomial fitting model was developed to calibrate concentration values using SWV peak current, pH, and ionic strength data for on-body measurement (note S1).

Flow characterization with dyes revealed three distinct operational phases (Fig. 3E). To verify these phases, we conducted flow tests using Cort (1 ng ml^−1^) at a flow rate of 1.5 μl min^−1^. During phase 1, all chambers sequentially filled, activating the timer electrodes. In phase 2, sweat and unbound labeled antigens flowed into the outlet channel, triggering the impedance (IMP)–based ionic strength sensor as excess salt and labeled antigens were released. Last, in phase 3, as all chambers refreshed, the IMP sensor readings plateaued because of the removal of excess salt. At this stage, SWV measurements were taken on W_C1_ to W_C5_, consistently yielding uniform peak current across all electrodes.

The multiplexed microfluidic patch’s ability to quantify stress hormone concentrations was further tested under two conditions. In the first scenario, Cort concentrations were maintained at a constant flow rate, either 1 or 10 ng ml^−1^, yielding consistent current readouts across all chambers with clear differentiation between the two concentrations [Fig. 3F(i)]. In the second scenario, dynamic concentration changes were introduced midrun by switching from 1 to 10 ng ml^−1^ and vice versa after the second chamber had filled [Fig. 3F(ii)]. The patch successfully detected these concentration shifts, demonstrating its capability to dynamically track hormone fluctuations in real time.

Previous studies have shown that sweat rates within an individual remain relatively consistent during the first hour after sweat induction (*28*). However, interpersonal variations (1 to 3 μl min^−1^) can affect incubation time in the microfluidic module. To address this, we investigated sensor performance across this flow range. The sensor consistently produced reliable readouts (fig. S21), aligning with earlier findings that sensor outputs stabilized after 15 min of incubation, irrespective of flow rate (fig. S12C).

While these results highlight the robustness of our sensing system, minor variability in absolute current values was occasionally observed across experimental runs. This is likely attributable to batch-to-batch differences in sensor fabrication, such as variations in electrode surface characteristics and protein immobilization efficiency. These factors can shift baseline signal levels while preserving relative detection performance (note S1). To improve interbatch consistency and quantitative reliability, future efforts will focus on fabrication process standardization, enhanced quality control measures, and implementation of batch-specific calibration strategies.

Given that the microfluidic sensor patch is intended for on-skin wearable applications, evaluating its cytocompatibility and biocompatibility is essential. Cytocompatibility was assessed by culturing cells in media containing the device extract, followed by viability analysis using a commercial live/dead assay and 3-(4,5-dimethylthiazol-2-yl)-2,5-diphenyltetrazolium bromide (MTT) assay (fig. S22). Representative live/dead staining images and absorbance measurements over a 7-day period demonstrated consistently high cell viability, confirming the excellent cytocompatibility of the sensor patch. To further evaluate biocompatibility, we performed an in vivo skin sensitivity test using a guinea pig model. The device extract was applied topically for a duration of 21 days, with saline and Freund’s complete adjuvant serving as negative and positive controls, respectively. Animals treated with the device extract showed normal weight gain during the testing period, and the application sites remained free of erythema, swelling, or ulceration (fig. S23). Histological examination of skin tissues from the application sites revealed intact epidermal and dermal layers without substantial inflammatory cell infiltration or tissue damage, confirming good biocompatibility (fig. S24).

### Pilot human studies under various stressors

Human studies were conducted to evaluate sweat stress hormone dynamics over time under various stress modulation scenarios. Cort, EPI, and NE concentrations in sweat were measured at five intervals: baseline (0 min), acute stress phase (10 min), and poststressor recovery (20, 30, and 40 min). Three representative stress modulation approaches were selected: high-intensity interval training (HIIT), psychological stress induced by negative International Affective Picture System (IAPS) images, and stress mitigation via supplement intake. Self-reported data were collected using the Positive and Negative Affect Schedule (PANAS) (*31*) and State-Trait Anxiety Inventory (STAI) (*32*) surveys at each interval to contextualize participants’ psychological responses alongside hormonal data (tables S2 to S32).

#### HIIT study

**Fig. 4.**
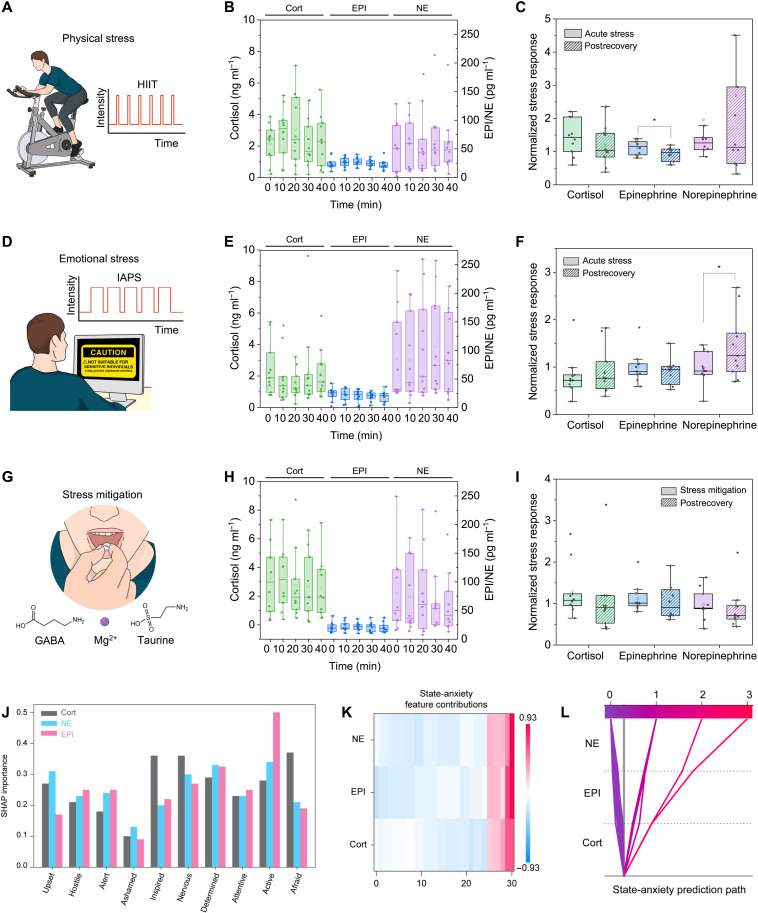
Preliminary human study on the stress hormone dynamics in response to physical, emotional, and pharmaceutical stress modulation. (**A**) Schematic of the HIIT protocol with alternating high- and low-intensity intervals in the first 10 min to induce acute physical stress. (**B**) Quantification of Cort, EPI, and NE levels at 0, 10, 20, 30, and 40 min during the HIIT study. (**C**) Baseline-normalized changes in Cort, EPI, and NE under acute stress phase (10 min) and postrecovery period (40 min) (*P < 0.05, *n* = 10). (**D**) Schematic of the IAPS protocol, showing exposure to emotionally provocative images in the first 10 min to introduce emotional stress. (**E**) Quantification of Cort, EPI, and NE levels at 0, 10, 20, 30, and 40 min during the IAPS study. (**F**) Baseline-normalized changes in Cort, EPI, and NE under acute stress phase (10 min) and postrecovery period (30 min) (*P < 0.05, *n* = 11). (**G**) Illustration of stress mitigation through supplement intake at 10 min. (**H**) Quantification of Cort, EPI, and NE levels pre- and postsupplementation. (**I**) Baseline-normalized changes in Cort, EPI, and NE under supplementation (10 min) and postrecovery period (40 min) (*n* = 10). (**J**) Relative biomarker feature importance based on SHAP values for predicting PANAS emotions. (**K**) Heatmap displaying relative SHAP feature contributions for state-anxiety prediction across training instances, grouped by hormone. (**L**) SHAP decision tree illustrating feature contributions to the predictive model for SA.

HIIT, characterized by intense exercise followed by short recovery periods, elicits strong HPA axis and SNS activation (Fig. 4A), resulting in increased plasma Cort and acute spikes in catecholamines (*33*, *34*). Sweat-based sensing effectively tracked these rapid hormonal fluctuations in real time, addressing the limitations of frequent blood sampling. Distinct temporal profiles for Cort, EPI, and NE were observed over the 40-min sampling period (Fig. 4B and fig. S25, A to C). Cort concentrations rose notably within the first 10 min, indicating an early HPA-axis response, and then gradually declined toward baseline by 40 min. EPI levels remained overall relatively low, suggesting only subtle or short-lived SNS activation. Meanwhile, NE showed a moderate upward trend and increased variability at later time points, potentially reflecting individual differences in sympathetic regulation and recovery. The temporal patterns in hormone levels align broadly with shifts in self-reported affectivity and anxiety (fig. S26A). Participants exhibited increases in positive affect (PA), negative affect (NA), and state anxiety (SA) scores corresponding to elevations in Cort and catecholamines. As stress hormones trended downward during recovery, PA scores rebounded while NA and SA declined. Given the variability among participants, stress hormone levels were normalized to each individual’s baseline (prestress) values. Normalized Cort levels showed a partial return toward baseline in postrecovery stage (30 min after stressor), reflecting the more prolonged recovery of HPA-axis activation. EPI, in contrast, dropped appreciably after the acute stress phase, indicating a faster return to baseline once the immediate stressor had subsided. NE values demonstrated considerable variability across participants even after normalization, suggesting individual differences in sympathetic regulation or recovery kinetics (Fig. 4C).

#### IAPS study

Psychological stress, relevant to mental health and cognition, was induced using negative images from the IAPS, a widely used database of standardized, emotionally charged images depicting aggression, mutilation, and other distressing scenes (Fig. 4D) (*35*). Cort levels showed a slight, nonsignificant change at 10 min, stabilizing through the 40-min period (fig. S25D). EPI levels remained unchanged, indicating that the psychological stressor did not trigger a significant epinephrine response. This may be attributed to the nature of the stressor and the distinct physiological source of epinephrine release. Epinephrine is predominantly secreted from the adrenal medulla into circulation and typically requires more intense stress for activation. In addition, epinephrine concentrations in sweat are typically present at extremely low baseline levels, often near the detection threshold of our sensors, making it difficult to resolve subtle changes under low-stress conditions. In contrast, NE demonstrated a progressive increase, suggesting prolonged SNS activation during emotional stimuli (Fig. 4E and fig. S25, E and F). Notably, SA scores showed a clear rise alongside NE at the onset of psychological stress, indicating that sympathetic activation aligned with heightened anxiety state (fig. S26B). A paired *t* test between acute stress and postrecovery conditions revealed a significant increase in NE during the postrecovery phase (*P* < 0.05), suggesting a more sustained sympathetic response compared to the other hormones (Fig. 4F). These findings align with previous research demonstrating that acute emotional stress primarily elevates NE, while EPI responses remain minimal (*36*, *37*).

#### Stress mitigation study

The effect of a stress-reducing supplement containing taurine, γ-aminobutyric acid (GABA), theanine, and magnesium was evaluated (Fig. 4G). These compounds function as inhibitory neuromodulators (*38*–*40*), dampening neural excitation. After supplement intake, sweat Cort concentrations gradually declined, suggesting a down-regulation of the HPA axis (Fig. 4H and fig. S25G). EPI levels remained relatively stable with no significant change throughout the observation period, and NE levels showed minimal decrease after supplementation (fig. S25, H and I). Notably, the participants’ self-reported stress scores did not demonstrate a significant change over the same period (fig. S26C). As no external stressors were introduced, the observed changes likely represent baseline modulation rather than acute stress attenuation (Fig. 4I).

Throughout all studies, box-and-whisker plots of sweat stress hormones revealed wide data distributions, especially in NE measurements. These differences are not experimental artifacts but instead reflect real and clinically relevant interindividual variability in both physiological regulation and psychological stress perception. Hormonal responses to stress are modulated by diverse factors, including baseline endocrine activity, genetic predisposition, psychological resilience, and prior stress exposure. Rather than masking this variability through averaging, our study embraces it as a key feature of human stress biology.

To further unpack this complexity, we begin addressing the distinction between eustress- and distress-stratified participants based on self-reported emotional states using validated PANAS and STAI scores. For each experimental condition (HIIT, IAPS, and stress modulation), we generated stratified group-averaged hormone trajectories for high and low affective subgroups (top and bottom 30%) and paired these with representative individual time-series data (figs. S27 to S30). These visualizations illustrate how hormone dynamics differ not only by stressor type but also by the subjective psychological context of each participant. For instance, individuals with high NA or SA often exhibited heightened or prolonged Cort and EPI responses, while those with higher PA showed more muted or rapidly resolving profiles, especially during the recovery condition.

These patterns suggest that the binary classification of stressors as “physical” or “emotional” may be less informative than understanding the underlying psychological interpretation of those stressors. In this light, the distinction between eustress and distress becomes less about the external trigger and more about the internal emotional and physiological signature. To explore this idea further, we developed a supervised machine learning framework that uses multivariate hormone features to predict participants’ emotional states, treating PANAS and STAI scores as ground-truth labels.

#### Affective computing with hormonal dynamics

Incorporating hormonal temporal dynamics into affective computing platforms enhances the ability to capture stress progression and recovery patterns, improving real-time applications in emotion regulation and therapy. To ensure robust and reliable classification of affective profiles, we processed the first 20 min of biometric feature signals, including baseline measurements, peak state-anxiety labels, and recovery scores, using a custom machine learning pipeline. Features and labels were preprocessed by taking the absolute difference in hormonal sweat concentration and affectivity scores. Each feature and label were normalized to have zero mean and unit variance, while extreme outliers were filtered out to maintain balanced representations. Aggregated test errors yielded mean accuracy values, with random forest (RF) models achieving strong predictive accuracies: 0.62 ± 0.098 for NA, 0.54 ± 0.089 for PA, and 0.86 ± 0.038 for SA. To interpret the RF models, SHapley Additive exPlanations (SHAP) were used to analyze the biomarker contributions (*41*). Cort, NE, and EPI were found to contribute nearly equally across all affective states (Fig. 4J), with Cort being the most crucial biomarker for distinguishing NA and NE playing a critical role in SA prediction. None of the biomarkers negatively influenced SA predictions. Notably, NE and EPI exhibited similar SHAP values for SA, indicating that each biomarker has unique, nonoverlapping information to contribute to the model (Fig. 4K), underscoring the importance of monitoring both hormones. In addition, no single biomarker dominated any prediction, with each hormone exhibiting a consistent contribution across all prediction instances (Fig. 4L).

### System integration and on-body evaluation of Stressomic

**Fig. 5.**
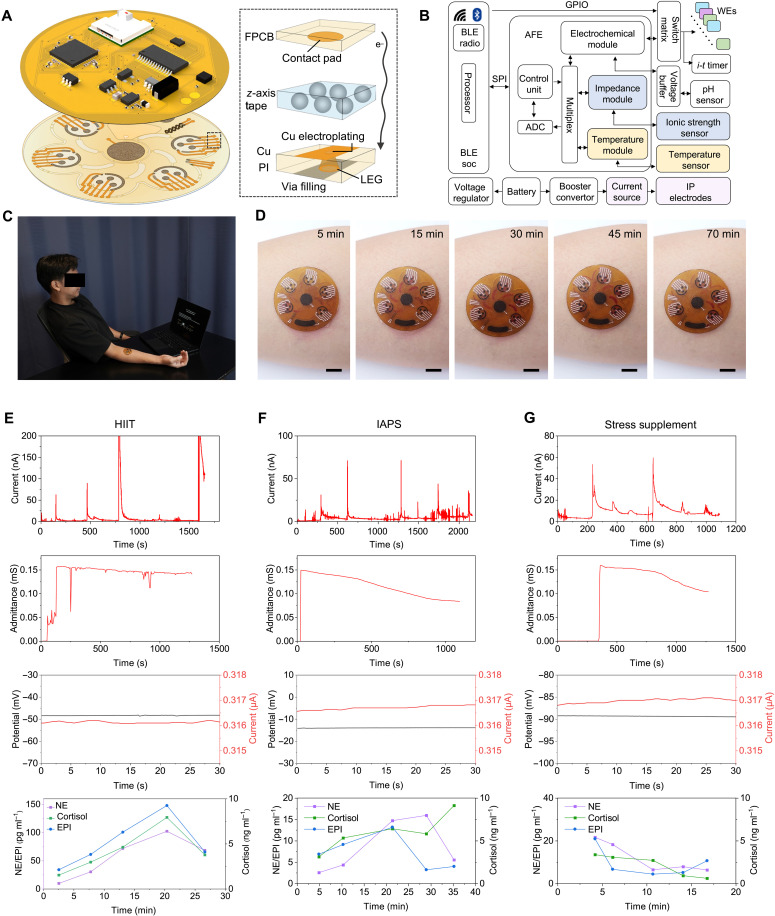
On-body evaluation of Stressomic for profiling stress hormone dynamics. (**A**) Schematic of the fully integrated Stressomic, showing the signal-processing electronics, contact pad, Cu electrodes, LEG, and microfluidic layer connected by *z*-axis tape. (**B**) Functional block diagram of the Stressomic system. Key components include the analog front-end (AFE) for electrochemical sensing, impedance measurement circuits, Bluetooth Low Energy (BLE) for wireless data transmission, and onboard processing units for real-time stress hormone analysis. ADC, analog-to-digital converter; soc, system on a chip; SPI, serial peripheral interface. (**C**) On-body testing setup with the device applied to a participant’s forearm, linked to a laptop for protocol guidance. (**D**) Optical images showing fluid dynamics and reagent flow in the microfluidic chambers on the skin over time. Scale bars, 1 cm. (**E**) On-body stress hormone profiling during the HIIT study, initiated after the first chamber filled. (**F**) On-body stress hormone profiling during the IAPS study, initiated after the first chamber filled. (**G**) On-body stress hormone profiling following stress supplement intake, showing attenuated stress responses postsupplementation.

The fully integrated Stressomic system features a vertical stack assembly that combines a flexible microfluidic sensor patch with a flexible printed circuit board (FPCB) in a compact, wearable design suitable for daily life (Fig. 5A). To enhance conductivity and minimize the device footprint, a double-layer PCB fabrication processing was used on a Cu–polyimide (PI) substrate (fig. S31). Cu contact pads and leads were patterned on the Cu side via laser etching, while UV laser drilling created vias for interlayer connections. On the PI side, graphene interconnects were patterned and electroplated to establish electrical pathways to the WEs, resulting in a flexible, double-sided, multifunctional sensor. Cu contact pads were aligned and connected to the FPCB via *z*-axis conductive tape, ensuring robust electrical connections. Electrochemical performance across 10 batches of electrodes demonstrated minimal batch-to-batch variation, confirming the consistency and reproducibility of the fabrication process (fig. S32).

Stressomic’s electronic system supports a wide range of functionalities, including current-controlled iontophoresis, multiplexed electrochemical measurements (e.g., voltammetry, impedimetry, and potentiometry), signal processing, and wireless communication (Fig. 5B). The system dynamically tracks responses from pH, ionic strength, skin temperature sensors, and the LEG-based timer, enabling real-time calibration for accurate stress hormone measurements.

On-body evaluation was conducted on healthy participants to assess Stressomic’s performance under real-world conditions (Fig. 5C). Initially, the system’s fluid handling capabilities were characterized using sweat flow generated by iontophoresis. The observed flow behavior mirrored that of in vitro syringe pump tests, confirming sequential filling and refreshing of red dye within the chambers. This demonstrated the system’s ability to effectively manage and direct sweat flow during on-body operation (Fig. 5D and movie S2). Next, we evaluated the sensor’s response under different stress conditions, including acute physical stress (HIIT), emotional stress (IAPS), and stress modulation through the intake of a stress relieving supplement (Fig. 5, E to G, and fig. S33). The sensor successfully captured real-time changes in stress hormone levels, showing substantial increases in Cort and NE in response to HIIT and a notable increase in NE level following emotional stimuli (Fig. 5, E and F). In the final study, the participant applied the Stressomic patch 10 min after the supplement intake, which reported a reduction in all three stress hormone levels (Fig. 5G). These findings demonstrate the sensor’s capability to monitor stress hormones across different stress conditions, validating its potential for real-time, noninvasive stress monitoring in everyday environments.

## DISCUSSION

Stressomic represents a major advancement in wearable, real-time stress hormone monitoring, enabling continuous tracking of Cort, EPI, and NE in sweat. By integrating a flexible microfluidic sensor patch with a multifunctional FPCB, the system effectively captures dynamic hormone fluctuations under various stress conditions, including physical stress, emotional stress, and supplement-induced stress modulation. This noninvasive approach highlights the system’s potential for continuous stress monitoring in everyday life.

Unlike previous systems constrained by single-use reagents, long incubation times, and manual replacements, Stressomic overcomes these limitations through CBVs and burst pressure gradients, autonomously regulating fluid flow and reagent release. This design facilitates real-time calibration, rapid detection, and the capture of transient hormone fluctuations, addressing issues associated with slow receptor-binding kinetics. The integration of multihormone electrodes and timer electrodes ensures precise tracking of dynamic hormone profiles. At the same time, the inclusion of pH, temperature, and electrolyte concentration sensors further enhances the system’s accuracy by correcting for environmental factors.

Under physical stress, Stressomic effectively captured increases in Cort and NE, reflecting HPA and SNS activation. In response to emotional stress, NE levels rose significantly, while cortisol exhibited a nonsignificant change. These findings corroborate prior studies, reinforcing the system’s ability to differentiate between physiological and psychological stress responses. In addition, the system tracked hormone reductions following supplement intake, demonstrating its ability to monitor changes in baseline regulation and therapeutic interventions.

While the platform shows strong performance in controlled studies, batch-to-batch variability remains an important consideration, particularly due to the porous morphology of LEG and the dendritic structure of the electroplated AuNDs used at the sensing interface. These features, while enhancing sensitivity through high surface area, introduce potential inconsistencies in surface chemistry, immobilization efficiency, and electrochemical behavior. In this study, fabrication protocols were standardized to reduce variability. To further enhance batch reproducibility, future work will incorporate systematic surface and electrochemical benchmarking [e.g., prescreening via cyclic voltammetry (CV) or SWV with redox probes] for real-time signal normalization.

The ability to continuously and noninvasively monitor stress hormones holds substantial implications for both clinical and personal health applications. For individuals with chronic stress or stress-related disorders, Stressomic provides a valuable tool for tracking hormone fluctuations and identifying early signs of stress overload. This could enable more personalized and proactive stress management approaches. For example, in athletic or rehabilitation contexts, Stressomic offers timely feedback on physical exertion and recovery, supporting tailored training regimens and improved recovery strategies. Furthermore, its ability to access emotional stress introduces opportunities for use in psychological research or therapeutic settings, offering insights into emotional resilience and coping mechanisms.

Beyond current applications, our framework lays the groundwork for advancing toward more nuanced distinctions between adaptive (eustress) and maladaptive (distress) stress responses. By integrating multihormone data with validated self-reported emotional states and applying machine learning to identify stress-related patterns, we demonstrate the potential for data-driven classification of stress valence. Building on this foundation, future studies could incorporate real-time behavioral feedback, ecological momentary assessments, and contextual information such as activity, sleep, and social interaction data. Longitudinal tracking and outcome-based validation will also be essential to refining physiological signatures and personalizing interpretations.

In summary, Stressomic introduces a transformative and noninvasive solution for real-time stress hormone monitoring. Its integration of microfluidic technology and flexible electronics allows for continuous hormone monitoring across physical, emotional, and pharmacological contexts, with broad applications in health monitoring and human performance management.

## MATERIALS AND METHODS

### Materials

Tetrachloroauric(III) acid trihydrate (HAuCl_4_·3H_2_O; Thermo Fisher Scientific) and ammonium chloride (NH_4_Cl; 99.6%; Acros Organics) were used for preparing AuNDs. 6-Mercapto-1-hexanol (MCH; 97%; Sigma-Aldrich) and BSA (≥98%; Sigma-Aldrich) were used for blocking the sensor surface. Pierce protein A (Thermo Fisher Scientific), protein G recombinant (≥90%; Sigma-Aldrich), 2-iminothiolane hydrochloride (≥98%; Sigma-Aldrich), spin column (G-Bioscience), Sephadex G-25 (Sigma-Aldrich), and EDTA (Sigma-Aldrich) were used for thiolation of protein A and G. Monoclonal mouse Cort antibody (MIC0201, Invitrogen), polyclonal rabbit anti-NE antibody (LS-C295847, LSBio), and polyclonal rabbit anti-epinephrine antibody (LS-C295834, LSBio) were used for capture antibodies. Hydrocortisone (≥98%; Sigma-Aldrich), norepinephrine small molecule (MBS2086359, MyBioSource), and epinephrine small molecule (MBS2086314, MyBioSource) were purchased as standard hormone solutions. (−)-EPI (Sigma-Aldrich), l-(−)-NE (+)-bitartrate salt monohydrate (≥99%; Sigma-Aldrich), HS-PEG-COOH (400 Da; Nanocs), *N*-ethyl-*N*′-(3-dimethylaminopropyl)carbodiimide hydrochloride (EDC-HCl; Sigma-Aldrich), *N-*hydroxysulfosuccinimide sodium salt (sulfo-NHS; ≥98%; Sigma-Aldrich), Cort 3-(O-carboxymethyl)oxime (Cort 3–CMO; NHS ester) (USBiological), Atto MB2 NHS ester (≥90%; Sigma-Aldrich), and Imject cationized BSA (cBSA; Thermo Fisher Scientific) were used for MB-labeled hapten synthesis. Agarose (Thermo Fisher Scientific), potassium chloride (KCl; 99%; Thermo Fisher Scientific), and carbachol (≥98%; Alfa Aesar) were used for the preparation of carbachol gels for iontophoresis. Silver/silver chloride paste (Ag/AgCl; 60/40), Cu(II) sulfate, potassium ferricyanide(III), potassium ferrocyanide(IV), polyvinyl butyral resin (PVB), sodium chloride (NaCl), and aniline were purchased from Sigma-Aldrich and used for electrode modification and characterization. Sodium dihydrogen phosphate (Thermo Fisher Scientific), potassium hydrogen phosphate (Thermo Fisher Scientific), phosphate-buffered saline (PBS; 10×, pH 7.4), isopropyl alcohol (IPA; 99%; Thermo Fisher Scientific), absolute ethanol (Thermo Fisher Scientific), Tween 20 (Sigma-Aldrich), sodium bicarbonate (Thermo Fisher Scientific), sodium carbonate anhydrous (Thermo Fisher Scientific), methanol (Thermo Fisher Scientific), sulfuric acid (H_2_SO_4_; 95 to 98%; Thermo Fisher Scientific), and hydrochloric acid (HCl; 36.5 to 38%; Thermo Fisher Scientific) were used for buffers and solvents. Human NE ELISA kit (EKC40270, Biomatik), human EPI ELISA kit (EKC33614, Biomatik), 96-well Nunc MaxiSorp flat-bottom plates, and 3,3′,5,5′-tetramethylbenzidine (TMB; Thermo Fisher Scientific) were used for ELISA validation for NE, EPI, and Cort, respectively. Medical adhesives were purchased from 3M. PET films (50- and 100-μm thick) were purchased from McMaster-Carr. Single-sided PI films (75-μm thick) were purchased from American Durafilm Co., Inc. Double-sided Cu-PI films (18/75-μm thick) were purchased from GBS Tape. 3M *z*-axis conductive tape was ordered from Adafruit.

### Fabrication of the multiplexed microfluidic stress immunosensor

A multiplexed microfluidic sensor patch was fabricated by engraving graphene on a PI film using a CO_2_ laser cutter (50 W; Universal Laser Systems). A PI film was raster-engraved to make iontophoresis, working, reference, and timer electrodes under 8% power, 20% speed, and 1000 points per inch. Counter electrodes were prepared under the same lasing parameters, but the lasing process was repeated three times. A PI film was vector-engraved for a pH sensor (2.5% power and 20% speed) and a temperature sensor (3% power and 15% speed). Connection leads and impedance electrodes were engraved using the same parameters for the pH sensors, but this process was repeated three times.

For the final on-body prototype patch, a single-sided Cu-PI film was patterned using a UV laser (LPKF ProtoLaser U4) and a CO_2_ laser cutter. Initially, a double-sided Cu-PI film was mounted on a UV laser plate and hatched to prepare Cu electrodes (90-kHz lasing frequency, 3-W power, 900-mm s^−1^ mark speed, and 9-μm hatching grid). The Cu patterned surface was then thoroughly rinsed with IPA, flipped, and placed on the plate to drill the via holes from the PI side (45-kHz frequency, 3-W power, 150-mm s^−1^ mark speed, and 1.5-mm focus offset). Alignment for each step was performed using four fiducials.

To connect the Cu and PI sides, graphene interconnects were engraved on the via holes using a CO_2_ laser (10% power and 20% speed). The graphene interconnects and via holes were then connected by electroplating Cu under 1 V for 10 min in a 1 M CuSO_4_ solution. The patch was subsequently realigned in the CO_2_ laser to engrave the multielectrodes. Last, the outline and outlet of the patch were cut using the UV laser (45-kHz frequency, 5.85-W power, 180-mm s^−1^ mark speed, and 0.1-mm focus offset).

The Ag/AgCl RE for the stress immunosensors was prepared by applying Ag/AgCl paste on the LEG electrode and annealing in a vacuum oven (60°C for 30 min). For the shared RE used for impedance and pH sensors, an additional PVB layer was deposited by dropcasting 0.4 μl of PVB solution, which was prepared by dissolving 79.1 mg of PVB and 50 mg of NaCl in 1 ml of methanol.

The pH sensor was prepared by electrochemical precleaning of the sensor in HCl (1 M) via CV scan from −0.2 to 1.2 V at 0.1 V s^−1^ for 10 cycles, followed by electrodeposition of polyaniline via CV from −0.2 to 1.2 V at 0.1 V s^−1^ for 10 cycles. The aniline solution was freshly prepared using 0.1 M aniline in 1 M HCl before the electrodeposition.

The iontophoresis hydrogel was prepared by dissolving 1% carbachol for the anode and 1% KCl for the cathode in an agarose solution, respectively. To prepare the agarose solution, 3% agarose was microwaved until fully dissolved, turning it into a transparent solution. The solution was then cooled on a hot plate (160°C at 1000 rpm), and 1% carbachol or 1% KCl was added. The mixture was poured into carbachol gel molds or assembled microfluidic patches, allowed to solidify at room temperature, and stored at 4°C until use.

To prepare the microfluidic module, the detection reservoirs were fabricated by stacking two layers of 50-μm medical adhesives, cutting under 80% power and 100% speed, and wiping the residue with IPA. For the detection reservoirs’ ceiling, 100-μm PET was treated with O_2_ plasma and attached onto the detection reservoir parts. Trigger valves were prepared by sandwiching 50-μm PET between two 50-μm medical adhesives and then cutting under various power and speed settings: surrounding channels (80% power and 100% speed), the first valve from chamber 1 (10% power and 10% speed), the remaining valves from chamber 1 (5% power and 10% speed), and valves from chambers 2 to 5 (4% power and 10% speed), based on the channels and valve widths. Reagent reservoirs were prepared by attaching two layers of 50-μm medical adhesives to the PET side of the detection reservoirs and cutting under 100% power and 80% speed. For the reagent reservoirs’ ceiling, 50-μm PET was O_2_ plasma–treated and cut for burst valve outlets for flow to the trigger valves. The assembly was completed by aligning and attaching the 50-μm PET (reagent reservoirs’ ceiling) between the trigger valve structure and the reagent reservoirs. Stop valves were then cut (100% power and 60% speed) and covered with 100 μm of PET. Last, a thick, petal-shaped flow path was cut (75% power and 50% speed) to complete the microfluidic modules. The redox probe–labeled competitors were dropcasted and vacuum-dried in the reagent reservoirs and stored in a dry state at 4°C until use.

### Protein A and protein G thiolation

Protein A (or G) was dissolved in 5 mM EDTA in 1× PBS to a final concentration of 10 mg ml^−1^. Iminothiolane stock (1.92 μl; 56 mM in 5 mM EDTA in 1× PBS) was added into 20 μl of protein solution and incubated for 1 hour at room temperature. The thiolated protein A (or G) was desalted using a G-25 spin column, which was equilibrated with the same buffer solution, and stored at −20°C for further use.

### Preparation of AuND-LEG–based stress immunosensors

AuNDs were electrodeposited on LEG electrodes by applying overpotential. The acidic Au precursor solution was prepared by dissolving 0.1 M HAuCl_4_ and 1 M NH_4_Cl in deionized (DI) water. Under the high overpotential (−3.5 V versus Ag/AgCl for 10 s), Au nanostructures were deposited using hydrogen bubbles as dynamic templates. The as-prepared electrodes were thoroughly rinsed with DI water for further modification. For the AuNP-LEG electrodes, AuNPs were electrodeposited by following our previous protocol (*28*).

The AuND-LEG WEs were functionalized with thiolated protein G (for Cort) and thiolated protein A (for NE and EPI) by dropcasting 5 μl of thiolated protein A or protein G (250 μg ml^−1^ in 5 mM EDTA in 1× PBS, pH 7.4) and incubating at room temperature for 2.5 hours. After rinsing with 1× PBS, the surface was blocked with 10 μl of 1 mM MCH for 1 hour and 10 μl of 1% BSA for 30 min at room temperature. Specific antibodies were then immobilized by dropcasting 5 μl of antibody solution (20 μg ml^−1^ for Cort cAb, 100 μg ml^−1^ for NE cAb, and 50 μg ml^−1^ for EPI cAb) onto the electrodes and incubating in a 37°C incubator for 1 hour. The functionalized electrodes were stored in 1× PBS until use.

### Redox probe conjugation

To prepare carboxylated catecholamine haptens, 12.5 mM NE bitartrate or EPI was dissolved in 0.1 M HCl solution and electrolyzed at 3 V until maximum conversion to *o*-quinone was observed (1 hour for NE and 2.5 hours for EPI). HS-PEG-COOH (12.5 mM; 400 Da) was dissolved into an *o*-quinone solution and stirred vigorously overnight. The reaction mixture was dialyzed in 10 mM MES buffer (pH 6.5) using a QuikPrep Ultra-Fast Dialyzer with 500-Da molecular weight cut-off cellulose acetate membrane to remove excess unmodified *o*-quinone and HS-PEG-COOH. For the activation of NE-COOH and EPI-COOH, 1 μl of EDC/sulfo-NHS solution (100 mM/200 mM in 0.1 M MES buffer, pH 5.5) was added to 10 μl of dialyzed NE-COOH or EPI-COOH, and the mixture was incubated in the dark at room temperature for 15 min to generate NE-NHS ester or EPI-NHS ester intermediates.

The conjugation reaction was carried out by colabeling the lysine residues on cBSA in the presence of both hapten-NHS ester and Atto MB2 NHS ester. Specifically for every 100 μl of reaction mixture, 1.68 μl of Atto MB2 NHS ester solution (40 mg ml^−1^) was combined with 10 μl of desalted cBSA (20 mg ml^−1^), 2 μl of hapten-NHS ester (EPI-/NE-NHS ester stock concentration or 10 mM Cort NHS ester), 2 μl of 0.1 M phosphate buffer (pH 8), and 84.32 μl of DI water. The reaction mixture was gently mixed and allowed to react at room temperature for 1 hour. Following the conjugation, the reaction mixture underwent buffer exchange to remove unreacted reagents. The first round of buffer exchange was performed with G-25 resin using DI water as the eluent to remove excess MB dyes and avoid dye precipitation. The buffer exchange step was repeated two more times using PBS as the buffer. The final conjugate was stored at −20°C for subsequent use.

#### Human sample analysis with sensors

Electrochemical detection via competitive binding of target stress hormones and MB-labeled competitors was performed by mixing stress hormone standards in 0.25× PBS (or raw biofluids) and MB-labeled competitors in 0.5 M phosphate buffer in a 9:1 ratio (v/v). Five microliters of mixture was dropcasted onto the WE for 15 min and rinsed with 1× PBS. SWV peaks were subsequently recorded in 1× PBS (pH 7.4). Benchtop experiments were conducted using electrodes with a 3-mm diameter (7.07-mm^2^ area), whereas wearable sensor measurements used miniaturized electrodes with a 1.4-mm diameter (1.54-mm^2^ area).

### Characterization

An electrochemical workstation (CHI660E, CH Instruments) was used to characterize surface modification of electrodes and electrochemical detection of stress hormones in buffer and biofluids. The morphology of the AuND was examined using a scanning electron microscope (Zeiss 1550VP FESEM). UV-vis absorbance data were collected using a UV-vis spectrophotometer (Thermo Fisher Scientific NanoDrop) to monitor redox probe labeling efficiency. ELISA validation tests were performed using a microplate photometer (accuSkan FC, Thermo Fisher Scientific) at a detection wavelength of 450 nm. Cu electroplating was performed using a dc power supply (SPS305, Kungber).

### Participants and procedures

The performance of the wearable sensor was evaluated using human sweat and blood samples collected from healthy participants, in compliance with the protocols approved by the institutional review board (numbers 21-1102 and 22-0768) at the California Institute of Technology (Caltech). Participants were recruited from the Caltech campus through advertisements by posted notices, word of mouth, and email distribution. All participants provided written, informed consent before participation in the study.

Healthy participants were asked to collect sweat under three different external stimuli: HIIT, IAPS, and stress modulation. Each study was conducted on a separate day. For the HIIT study, participants performed an intense stationary bike exercise consisting of 10 s of intense biking followed by 30 s of rest, repeated for 10 rounds. Sweat samples were collected before, during, and after the HIIT protocol. For the IAPS study, participants were exposed to emotionally negative images assisted by auditory stimuli to induce a fight-or-flight reaction. The negative images for the IAPS study were provided by the National Institute of Mental Health Center for Emotion and Attention (*42*). During the session, 20 pictures were presented for 30 s each, with a 3-s interpicture interval. Sweat samples were collected before, during, and after the IAPS protocol. For the stress modulation study, participants consumed two supplemental pills—Taurine 1000 mg from NOW Foods and Theanine Serene with Relora from Source Naturals—to promote calmness and relaxation. These supplements contained taurine and other calming agents, and sweat samples were collected before, during, and after consuming pills.

#### Sample collection for in vitro studies

Sweat collection was performed via iontophoresis using a Model 3700 Macroduct sweat collection system. Participants wore a microduct collector for 30 to 60 min for sweat collection. Fresh blood samples were collected using a finger-prick approach with lancets. Blood samples were transferred to low-bind microtubes and allowed to clot at room temperature for 1 hour. After the clotting process was complete, serum was separated by centrifugation at 1250*g* for 15 min. The supernatant was immediately collected and stored at −80°C.

#### Human sample analysis with ELISA

ELISA tests for NE and EPI were performed according to the manufacturer’s instructions. Cort ELISA was conducted following a laboratory-based protocol. Briefly, a 96-well plate was modified with anti-Cort antibodies (2.5 μg ml^−1^) in 50 mM carbonate buffer (pH 9.6) and stored overnight at 4°C. The plate was then washed three times with 300 μl of PBST (1× PBS with 0.05% Tween 20). To block nonspecific binding, 300 μl of 1% BSA in PBST was added to each well and incubated for 1 hour at room temperature. After three washing steps with washing buffer (PBST, pH 7.4), 50 μl of Cort standards (or diluted biofluid samples) and 50 μl of Cort–horseradish peroxidase (diluted 250-fold in PBST) were added to each well and incubated for 1 hour at room temperature. Following another three washing steps with PBST, 100 μl of TMB substrate was added to each well and incubated at room temperature until color developed. The reaction was stopped by adding 50 μl of H_2_SO_4_ (1 M), and absorbance values were measured at 450 nm using a microplate photometer.

#### On-body wearable evaluation

The Stressomic system was exclusively used for the on-body studies presented in Fig. 5. The participants’ arm was cleaned with alcohol swabs before the wearable sensor patches were placed on the body. A 5-min iontophoresis was applied to the participants. During the on-body trial, the sensor system continuously acquired and transmitted chronoamperometry data from the timer electrodes. When the chronoamperometry data indicated the complete filling of five chambers, the system proceeded with impedance measurement on the ionic strength sensor. After the ionic strength sensor readings plateaued, the sensor system would conduct SWV scans over the stress hormone sensors sequentially in each chamber, followed by the acquisition of the readings of the pH sensor. After being calibrated in real time using simultaneously collected electrolyte and pH information, the acquired data were converted to the concentration levels based on the calibration curve.

#### Electronic circuit system design

A dual-layer FPCB was designed and fabricated using EAGLE CAD. This circular-shaped FPCB establishes electrical connections between its interface section and the sensor electrode section via *z*-axis conductive adhesive tape. This ensures stable signal transmission and mechanical flexibility. The FPCB integrates multiple functional modules, enabling efficient electrochemical sensing and wireless data transmission. In terms of power management, a voltage regulator (ADP162, Analog Devices) was used to provide a stable operating voltage to the system. To facilitate iontophoresis-based sweat induction, the system integrates a boost converter (TPS61096, Texas Instruments), a bipolar junction transistor array (BCV62C, Nexperia), and an analog switch (DG468, Vishay Intertechnology) to generate and control the current required for iontophoresis. The sensor interface includes an analog front-end (AFE; AD5941, Analog Devices) and an operational amplifier (LPV811, Texas Instruments) for signal conditioning and data acquisition. In addition, the system incorporates multichannel switches (ADG706, Analog Devices, and TMUX1112, Texas Instruments) to control the connection and switching of multiple electrodes, enhancing system flexibility and scalability. The core control and communication functionalities of the system are implemented using a Bluetooth Low Energy (BLE) module (CYBLE-222014-01, Cypress Semiconductor), which is programmed via PSOC Creator v4.3 to support wireless data transmission and system control. The BLE module can initiate the current for iontophoresis electrodes to induce sweat generation via its general-purpose input/output (GPIO) pins. The iontophoresis device is designed to deliver a controlled and constant current to the skin and operates at a compliance voltage of 18 V, enabling stable performance under various skin impedance conditions. The system uses a temperature module built with the AFE and a potential divider to record skin temperature. After sweat stimulation is complete, the BLE module controls the AFE to execute various electrochemical techniques, including potentiometric analysis, EIS, chronoamperometry, and SWV, for acquiring high-precision stress hormone data as well as pH and ionic strength, thereby calibrating sensor data. Once a certain amount of sensor data is stored in the AFE’s internal memory, the AFE triggers an interrupt signal to notify the BLE module to read the data and wirelessly transmit it to a custom mobile application.

#### Machine learning pipeline for affectivity assessment

Sweat-based biochemical features and self-report labels were processed to assess stress-related affective states. Three features—absolute changes in NE, Cort, and EPI concentrations—were extracted from each subject’s sweat samples. Corresponding labels were derived from self-reported state-anxiety scores (STAI) and PANAS. Rather than using raw values, we computed the absolute difference in each variable across time points (e.g., prestress versus poststress), capturing dynamic physiological and affective changes for classification. To ensure comparability across features and labels, all variables were standardized to have zero mean and unit variance. Data points associated with extreme affective scores that appeared only once per class were excluded to avoid overfitting outliers and to maintain a more balanced label distribution. The total sample size for the pilot study was 31, with each data point representing a complete feature-label instance. The data were partitioned into training and testing sets using a 9:1 split ratio, stratified to ensure balanced representation across affective classes. An RF classifier comprising 100 decision trees was used, selected for its robustness with small datasets and ability to capture nonlinear relationships.

Model training and evaluation were implemented in Python 3.8. To assess generalizability and reduce bias from a single train-test split, we performed 250 iterations of randomized stratified splitting. For each iteration, model performance was evaluated on the test fold, and accuracy scores were aggregated to compute a mean performance metric across the full distribution of splits. This iterative strategy provided a robust estimate of average model performance and reduced variance in reported outcomes. No questionnaire scores were used as predictive input features; rather, they served solely as ground-truth labels for supervised learning. This design allowed the model to classify affective state changes based purely on biochemical inputs, laying the foundation for future wearable systems that operate independently of self-report instruments.

## Supplementary Materials

### The PDF file includes:

Note S1 Figs. S1 to S33 Tables S1 to S32 Legends for movies S1 and S2 References
