# Supplementary material for: Stressomic: A wearable microfluidic biosensor for dynamic profiling of multiple stress hormones in sweat
Source: Sci Adv. 2025 Aug 6;11(32):eadx6491. doi: 10.1126/sciadv.adx6491 (PMC12327446; doi:10.1126/sciadv.adx6491)
Supplement: Supplementary file 1 — Note S1 Figs. S1 to S33 Tables S1 to S32 Legends for movies S1 and S2 References [file sciadv.adx6491_sm.pdf]

Science Advances
AAAS

Supplementary Materials for

Stressomic: A wearable microfluidic biosensor for dynamic profiling of multiple stress hormones in sweat

Jiaobing Tu *et al.*

Corresponding author: Wei Gao, weigao@caltech.edu; Dong-Hwan Kim, dhkim1@skku.edu

Sci. Adv. **11**, eadx6491 (2025)
DOI: 10.1126/sciadv.adx6491

The PDF file includes:

Note S1
Figs. S1 to S33
Tables S1 to S32
Legends for movies S1 and S2
References

Other Supplementary Material for this manuscript includes the following:

Movies S1 and S2

Note S1. Multivariate calibration for Stressomic

Polynomial fitting for matrix effect

At relevant physiological skin temperatures (33–37°C) and concentrations (10 pg ml^{-1} epinephrine, 10 pg ml^{-1} norepinephrine and 1 ng ml^{-1} cortisol), we find that the signal changes are minimal (4.24% for epinephrine, 0.73% for norepinephrine, 1.54% for cortisol). Because of this low variation, we do not calibrate for temperature. We employed a regression-based approach to obtain a calibration equation that relates the stress hormone concentration to the measured SWV peak current, ionic strength, and pH sensor responses. To capture the influence of various experimental factors on the sensor response, we constructed a feature matrix ***X***, which includes the logarithmic concentration along with other relevant variables based on the matrix effect studies (fig. S20). The columns of ***X*** include: The logarithmic concentration ($\log_{10} x$), the ionic strength (E), the pH (pH) and the squared values of ionic strength (E^2) and pH (pH^2) to account for non-linear effects observed in the matrix effect studies. The feature of X can be expressed as:

$$\boldsymbol{X} = [\log_{10} x, E, pH, E^2, pH^2] \qquad \text{S1}$$

where each column represents a different feature that influences the sensor response.

We used a polynomial fitting function in Matlab, *polyfitn*, to fit a linear model to the data. The model predicts the SWV current response variable, ***Y***, based on the feature, ***X***. In this case, we fit a first-degree polynomial (linear model) with the form:

$$\boldsymbol{Y} = a_1 \cdot \log_{10} x + a_2 \cdot e + a_3 \cdot pH + a_4 \cdot e^2 + a_5 \cdot pH^2 + b \qquad \text{S2}$$

where a_1, a_2, a_3, a_4, a_5 are the model coefficients, and b is the intercept. The model parameters are organized in a parameter matrix $[a_1, a_2, a_3, a_4, a_5, b]$.

The model parameters for each stress hormone are as follows: for epinephrine, the coefficients are [-0.0616, 0.0034, 0.0106, -8.3945e-06, -0.0023, 0.1135], for cortisol, the coefficients are [-0.1161, 0.0130, 0.0884, -5.0030e-05, -0.0065, -0.2392], for norepinephrine, the coefficients are [-0.0717, 0.0054, 0.1252, -8.6909e-06, -0.0094, -0.3640].

The calibration models perform well with an R^2 value of 0.9546 for epinephrine, 0.9532 for cortisol, and 0.9805 for norepinephrine. To predict the target concentration, we solve the calibration equation derived from the polynomial regression model.

Batch-to-batch calibration

To address potential variability between fabrication batches, a standardized batch-level calibration can be performed. Specifically, for each new fabrication batch, 2–3 representative sensors can be selected and calibrated against three or more standard solutions spanning the physiological concentration range.

For a given batch-specific calibration curve and standardized calibration

$$signal_{batch} = m_{batch} \log([target]) + b_{batch} \qquad \text{S3}$$

$$signal_{standard} = m_{standard} \log([target]) + b_{standard} \quad \text{S4}$$

where m is the slope of the calibration curve and b is the intercept:

One could convert the raw electrochemical signals into batch-normalized standard signals using the following transformation:

$$signal_{standard} = \frac{m_{standard}}{m_{batch}}(signal_{batch} - b_{batch}) + b_{standard} \quad \text{S5}$$

This step corrects for fabrication variability without requiring per-device calibration and ensures that sensor responses from different batches can be directly compared.

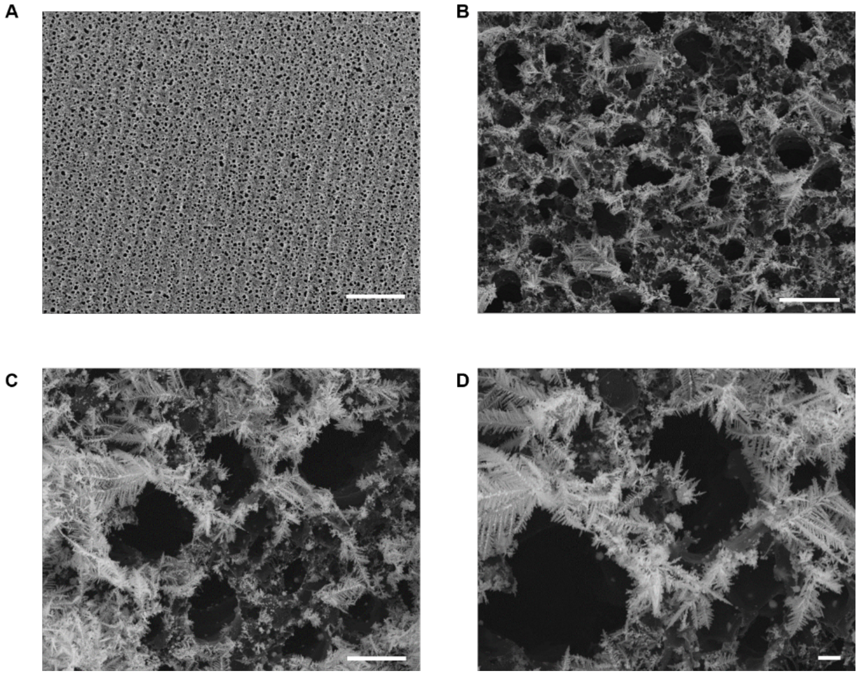

Fig. S1. SEM images of AuNDs-LEG electrodes at varying magnifications. Scale bars, 200 μm (**A**), 20 μm (**B**), 10 μm (**C**), and 2 μm (**D**).

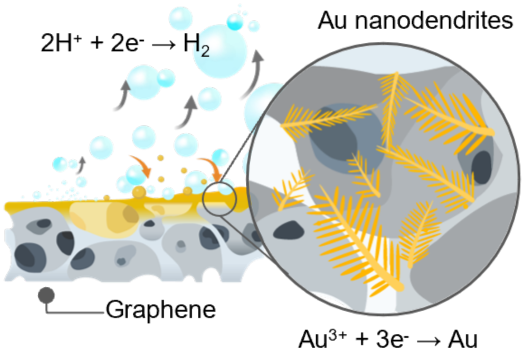

Fig. S2. Schematic illustration of AuND electrodeposition on LEG electrodes.

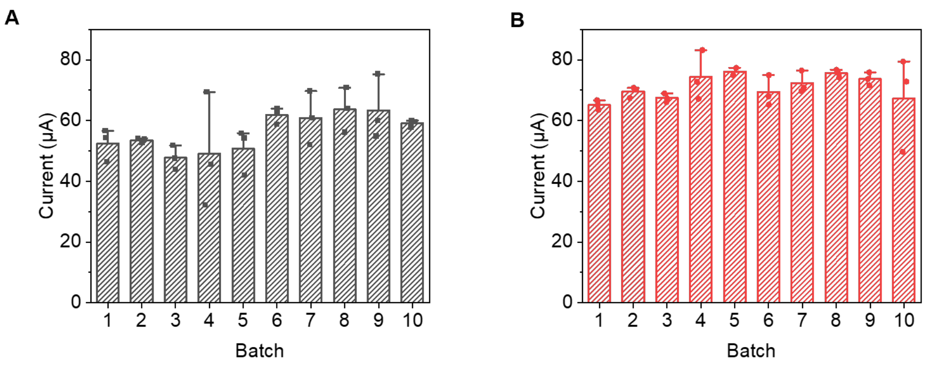

Fig. S3. Batch-to-batch variation of LEG and AuNDs-LEG electrodes. (**A** and **B**) Oxidative peak height of LEG (A) and AuNDs-LEG (B) electrodes measured by cyclic voltammograms in 0.1 M KCl and 5 mM $[Fe(CN)_6]^{3-}$. Error bars represent the s.d. of the mean from 3 sensors.

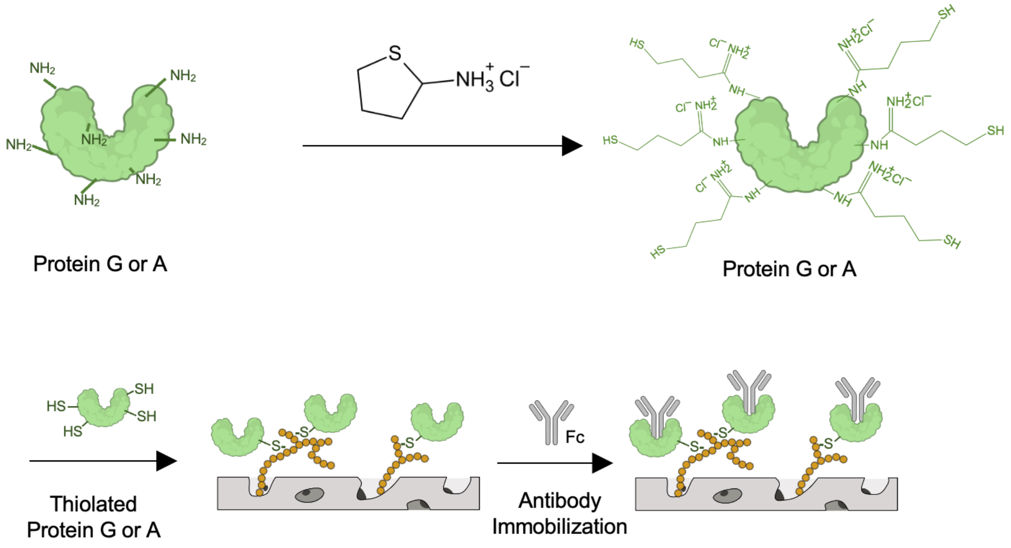

Fig. S4. Schematic illustration of protein A or protein G thiolation and immobilization. Thiolated protein A or protein G is immobilized on the AuNDs via thiol-gold bonding, followed by antibody binding to the protein A or protein G.

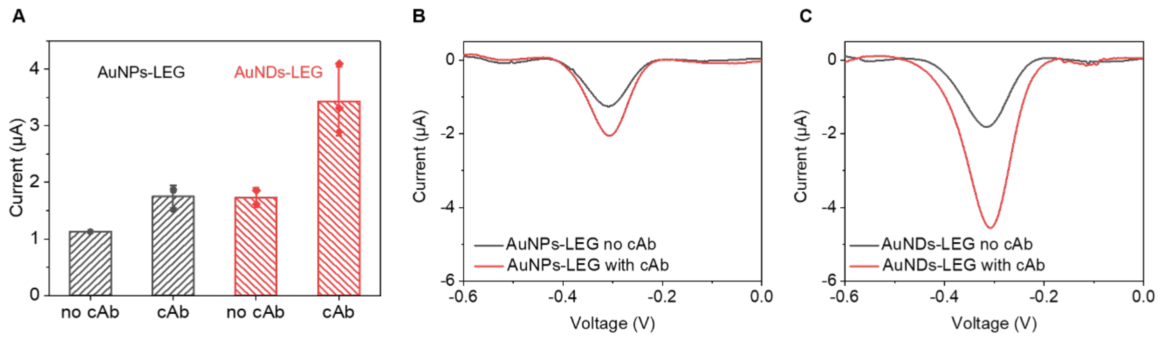

Fig. S5. Comparison of AuNPs-LEG and AuNDs-LEG based sensors. (**A**) Comparison of current intensities for norepinephrine sensors prepared using AuNPs-LEG and AuNDs-LEG electrodes. Control samples (no cAb) were prepared without protein A and norepinephrine capture antibody, while norepinephrine sensors (cAb) were prepared with protein A and norepinephrine capture antibody. Error bars represent the s.d. of the mean from three sensors. (**B** and **C**) Representative SWV voltammograms of norepinephrine sensors using AuNPs-LEG (B) and AuNDs-LEG (C) electrodes. Sensors were incubated in 0 ng ml^{-1} of norepinephrine in 0.25× PBS (pH 7.4) and detected in 1× PBS (pH 7.4).

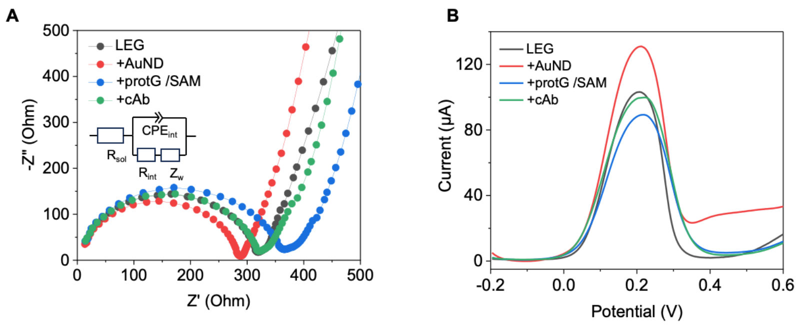

Fig. S6. Electrochemical impedance spectroscopy (EIS) and differential pulse voltammogram (DPV) of the stress immunosensor after each modification step. (**A** and **B**) EIS (A) and DPV (B) measurements conducted successive modification steps: pristine LEG, AuND electrodeposition, protein G immobilization and surface blocking with a self-assembled monolayer (SAM), and cAb immobilization. EIS tests were performed in 0.1 M KCl containing 5 mM $[Fe(CN)_6]^{3-}$ at open circuit potential with an alternating current amplitude of 5 mV, across a frequency range of 0.1–1,000,000 Hz. The inset in (A) shows the equivalent circuit used for fitting, consisting of solution resistance (R_{sol}), interfacial resistance (R_{int}), constant phase element (CPE_{int}), and Warburg impedance (Z_t). DPV measurements were conducted in 0.1 M KCl containing 5 mM $[Fe(CN)_6]^{3-/4-}$ (1:1 v/v).

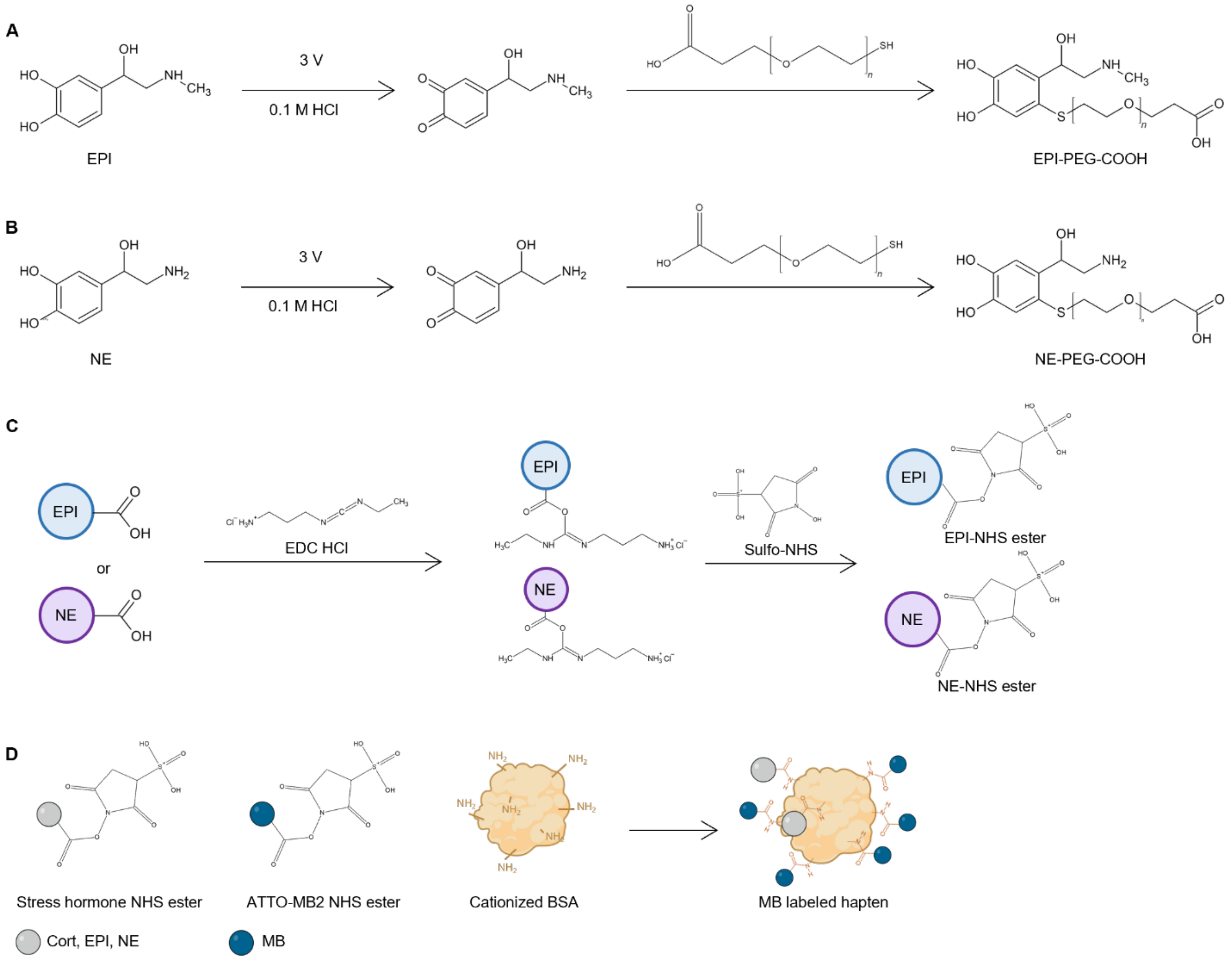

Fig. S7. Synthesis of functionalized haptens and conjugation of redox-active competitors. (**A** and **B**) Oxidation of catecholamine epinephrine (A) and norepinephrine (B) and subsequent modification with thiol-PEG-COOH. (**C**) Formation of EPI-NHS ester and NE-NHS ester intermediates using EDC/NHS chemistry. (**D**) Conjugation of methylene blue (MB)- and stress hormone-NHS esters onto cationized bovine serum albumin (BSA) to form MB-labeled competitors.

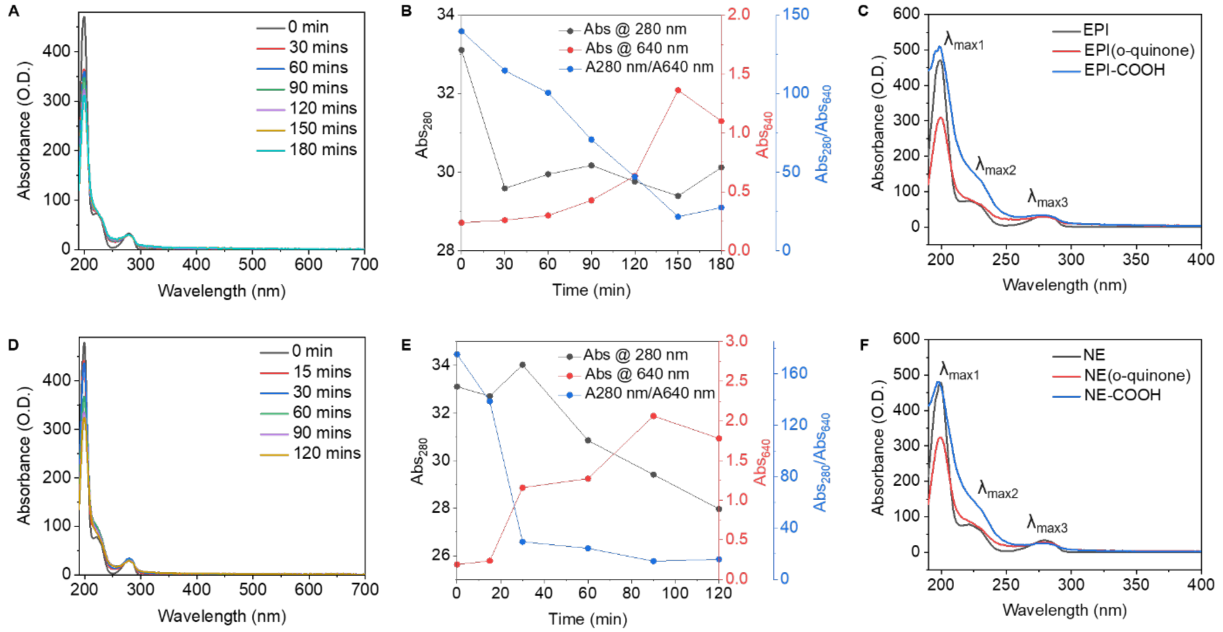

Fig. S8. Ultraviolet–visible absorbance spectra of catecholamines upon electrolysis and ring conjugation. Absorbance spectra of epinephrine (**A**) and norepinephrine (**D**) haptens were measured at varying electrolysis durations. Oxidation leads to the formation of bright-orange o-quinone intermediates with increasing absorbance at 640 nm and a decrease in characteristic catecholamine peak at 280 nm for epinephrine (**B**) and norepinephrine (**E**). By taking the ratio of both peaks, optimal electrolysis duration can be determined by taking the time point at which the ratio starts to plateau. Beyond the optimal electrolysis time, norepinephrine hapten tends to form a black, melanin-like polymer precipitate. Successful direct ring-conjugation of thiol-PEG-COOH to epinephrine (o-quinone) (**C**) and norepinephrine (o-quinone) (**F**) intermediates results in the recovery of the characteristic absorbance peak at 200 nm (λ_{max1}).

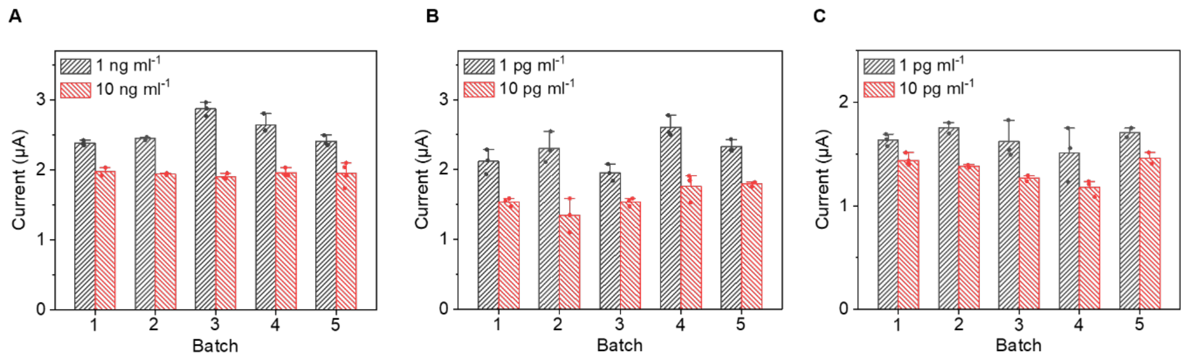

Fig. S9. Reproducibility of stress immunosensors across sensor batches. (**A** to **C**) Reduction peak current height in the SWV voltammograms for cortisol sensors measured at 1 and 10 ng ml^{-1} (A), and epinephrine (B) and norepinephrine (C) sensors measured at 1 and 10 pg ml^{-1} in 1× PBS (pH 7.4) across 5 sensor batches for each concentration. Error bars represent the s.d. of the mean from 3 electrodes.

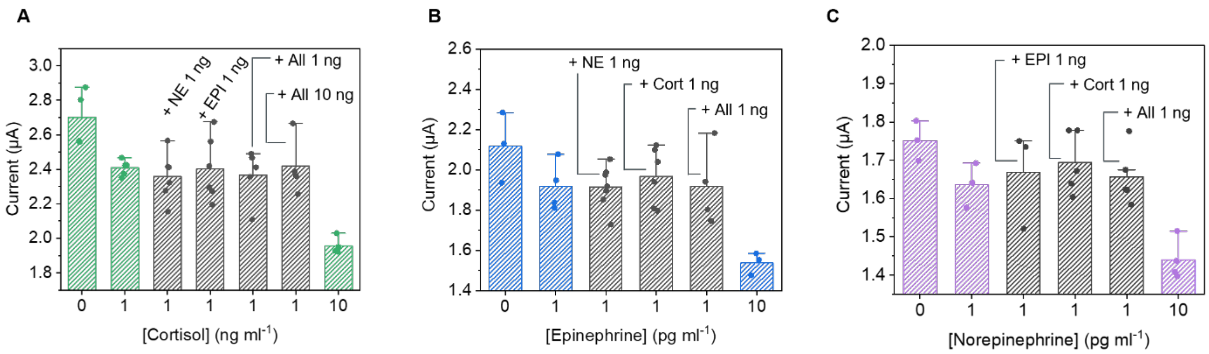

Fig. S10. Interference test of the stress immunosensors against other stress hormones. (**A** to **C**) Interference tests for cortisol (A), epinephrine (B), and norepinephrine (C) sensors were conducted by introducing interferents. For cortisol sensors, 1 ng ml^{-1} of norepinephrine and epinephrine were individually added, along with mixed concentrations of 1 and 10 ng ml^{-1} of norepinephrine and epinephrine, in the presence of 1 ng ml^{-1} of cortisol. For epinephrine and norepinephrine sensors, interference tests were conducted by adding 1 ng ml^{-1} of individual and mixed stress hormones in the presence of 1 pg ml^{-1} of epinephrine or norepinephrine, respectively. Error bars represent the s.d. of the mean from 3 to 5 sensors.

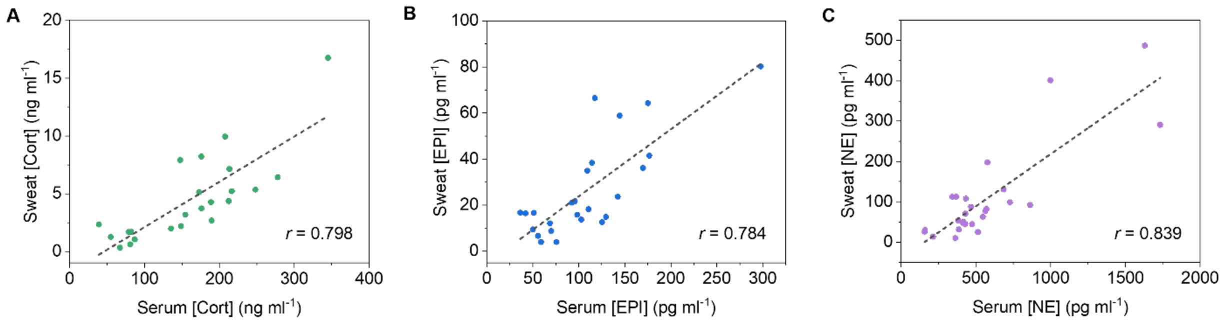

Fig. S11. Individual Pearson's correlation analysis of sweat and serum stress hormones. (**A to C**) Correlation between serum and sweat stress level of cortisol (A), epinephrine (B), and norepinephrine (C). Sweat stress hormone concentrations were determined using the stress immunosensors, while serum hormone levels were determined by ELISA. The correlation coefficient r was acquired through Pearson's correlation analysis (Cort, n=23; EPI, n=25; NE, n=26, P<0.00001). Sweat and serum samples were collected at the same time point.

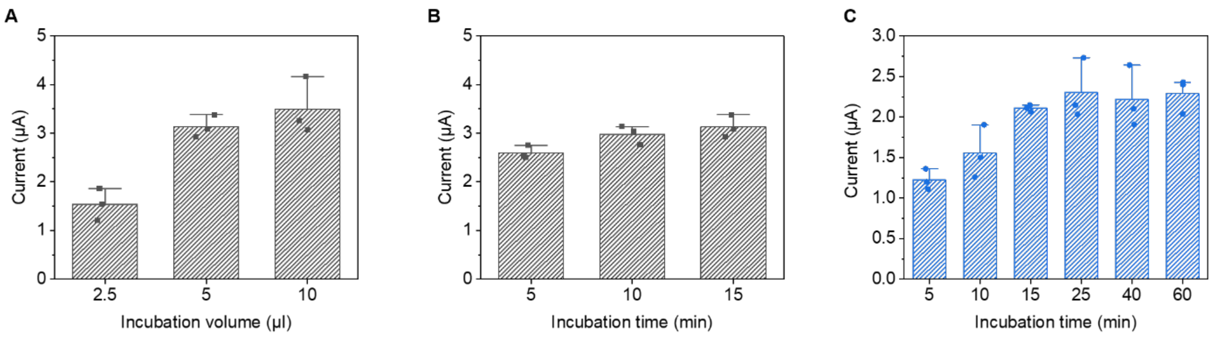

Fig. S12. Influence of incubation volume and time on stress sensor responses. (**A** and **B**) Cortisol sensor response on single electrodes (3 mm diameter) at 0 ng ml^{-1} of cortisol measured in 1× PBS (pH 7.4) under varying incubation volumes (A) and incubation times (B). (**C**) Cortisol sensors on multielectrodes (1.8 mm diameter) at 0 ng ml^{-1} of cortisol measured in 1× PBS (pH 7.4) under different incubation times. Error bars represent the s.d. of the mean from 3 sensors.

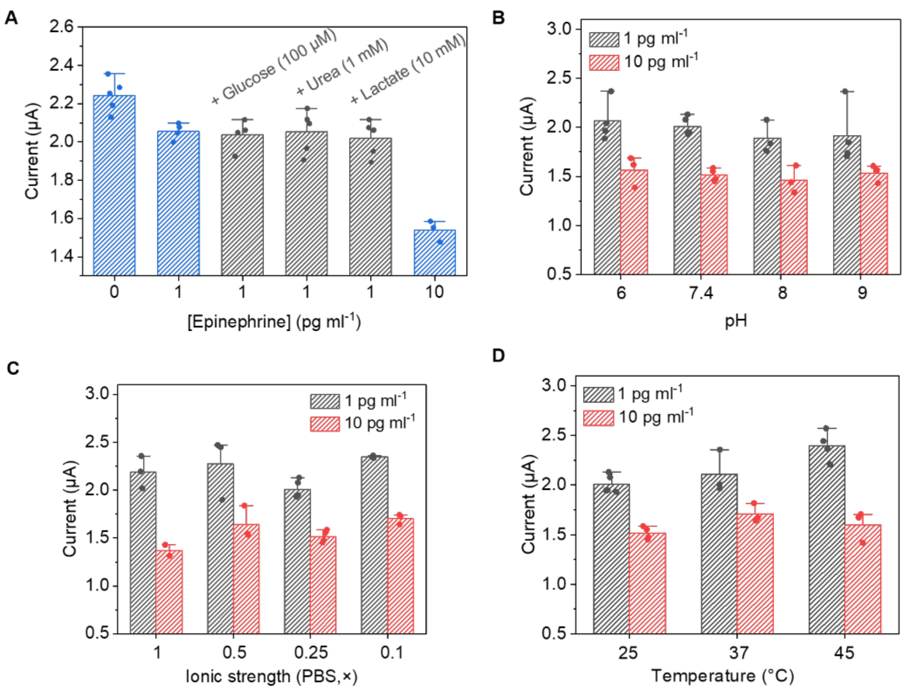

Fig. S13. Effect of potential interferents and incubation pH, ionic strength, and temperature on stress sensor responses. (**A**) SWV peak current of epinephrine sensors in the presence of glucose, urea, and lactate. Interference test was performed by adding potential interferents in 1 pg ml^{-1} of epinephrine. (**B**) SWV peak current height in the SWV of epinephrine sensors incubated with 1 and 10 pg ml^{-1} of epinephrine in buffer adjusted to pH 6–9 (0.25× PBS, 25 °C). (**C**) SWV peak current of epinephrine sensors incubated with 1 and 10 pg ml^{-1} of epinephrine in buffer with varying ionic strengths (0.1–1× PBS, pH 7.4, 25 °C). (**D**) SWV peak current of epinephrine sensors incubated with 1 and 10 pg ml^{-1} of EPI under different incubation temperatures (25–45 °C) in 0.25× PBS (pH 7.4) for 15 min. Incubation involved mixing standard epinephrine solution with EPI-MB redox probe at a 9:1 volume ratio. The EPI-MB was prepared by diluting redox probe stock in 0.5 M phosphate buffer (pH 7.4). Detection was performed in 1× PBS (pH 7.4) at 25°C. Error bars represent the s.d. of the mean from 3−5 sensors.

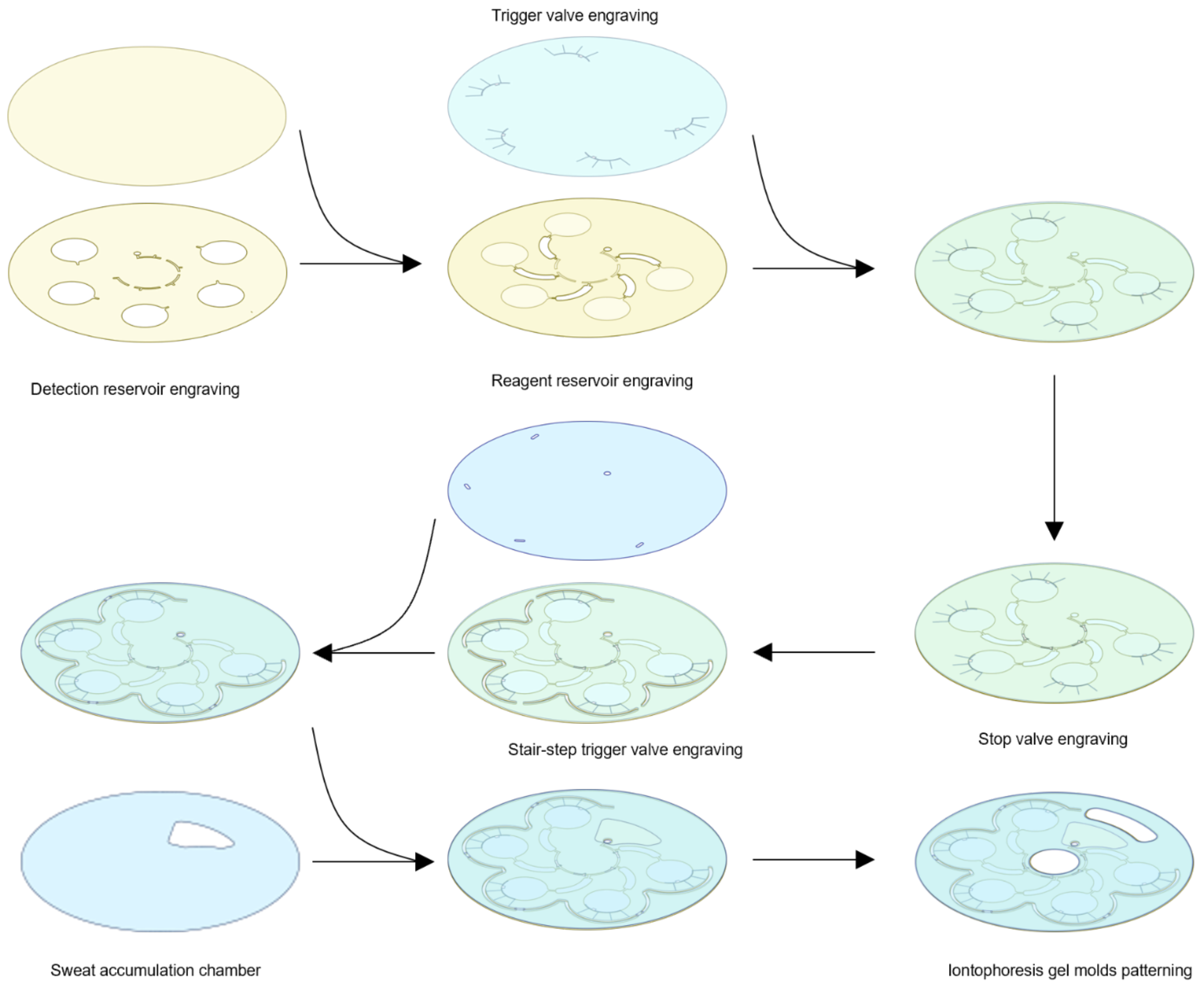

Fig. S14. Fabrication process of the microfluidic assembly. The fabrication begins with the detection reservoir engraving to define sensing regions. This is followed by trigger valve engraving to create channels that control fluid flow initiation and stoppage. Reagent reservoir engraving is performed to incorporate storage areas for necessary reagents required for analysis. Stop valve engraving ensures precise regulation of flow movement within the microfluidic device. Stair-step trigger valve engraving facilitates sequential fluid delivery by incorporating flow-triggering mechanisms. The sweat accumulation chamber is fabricated to collect and direct sweat into the microfluidic channels. Finally, iontophoresis gel mold patterning is applied to the assembled PET-tape assembly, enabling hydrogel casting for sweat induction and sampling.

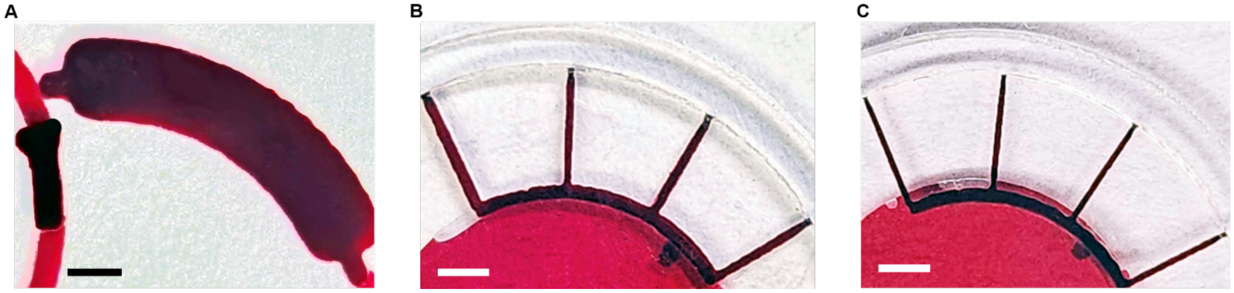

Fig. S15. Optical images of microfluidic valves. (**A** to **C**) Optical images of the stop valve (A) and stair-step trigger valves at the first (B) and second (C) detection reservoirs. Scale bars, 1 mm.

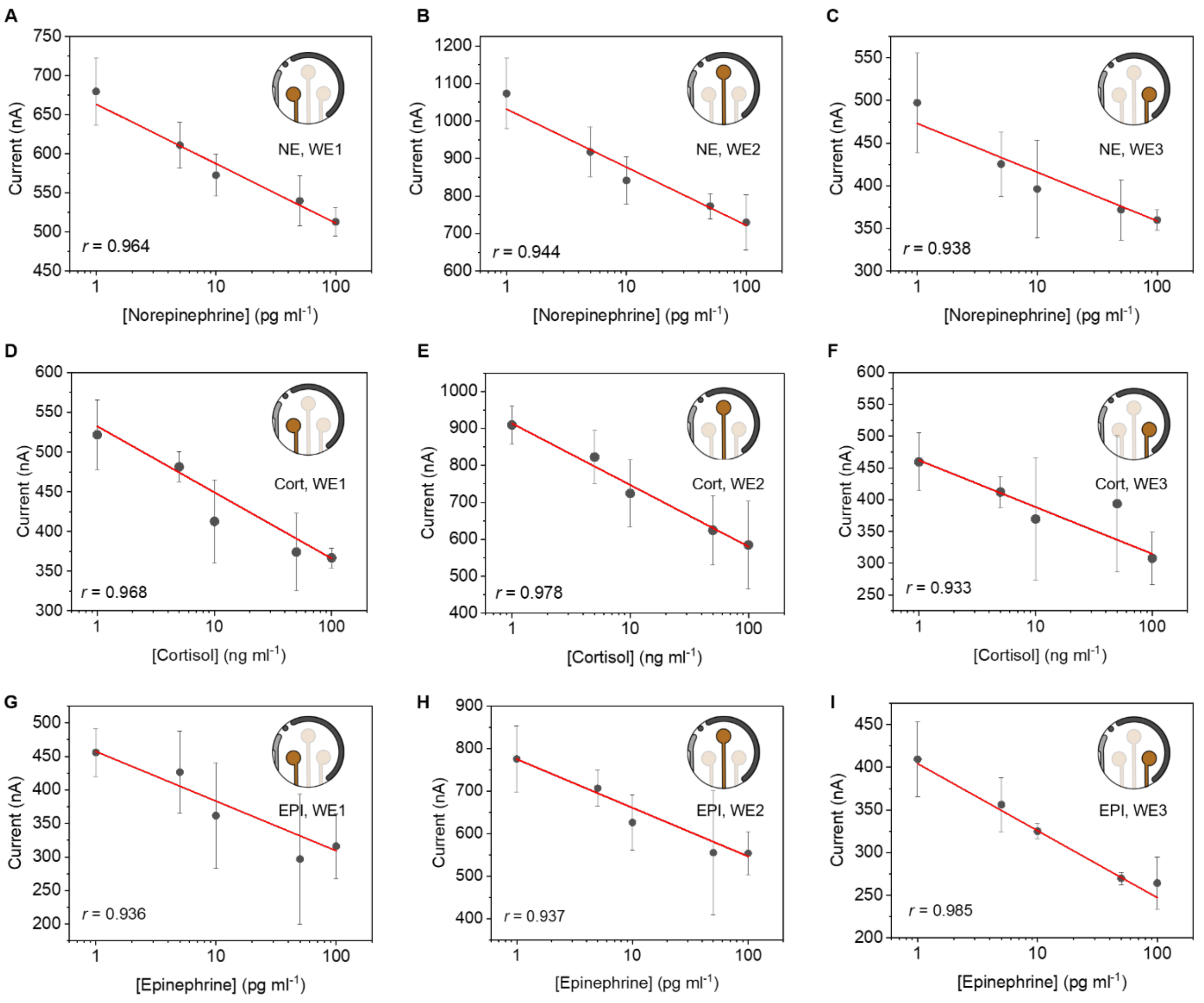

Fig. S16. Calibration of stress immunosensors in a multiplexed sensor platform. (**A** to **C**) Calibration of norepinephrine sensors on working electrodes WE1 (A), WE2 (B), and WE3 (C). (**D** to **F**) Calibration of cortisol sensors on WE1 (D), WE2 (E), and WE3 (F). (**G** to **I**) Calibration of epinephrine sensors on WE1 (G), WE2 (H), and WE3 (I). Incubation was performed by mixing standard stress hormones (prepared in 0.25× PBS, pH 7.4) with all 3 mixed redox probes (NE-MB, Cort-MB, and EPI-MB) at a 9:1 volume ratio. The mixed redox probe mixture was prepared by diluting each redox probe stock in 0.5 M phosphate buffer (pH 7.4). Detection was performed in a static chamber without flow with 5 µl of 1× PBS (pH 7.4) at 25°C. Error bars represent the s.d. of the mean from 3 sensors.

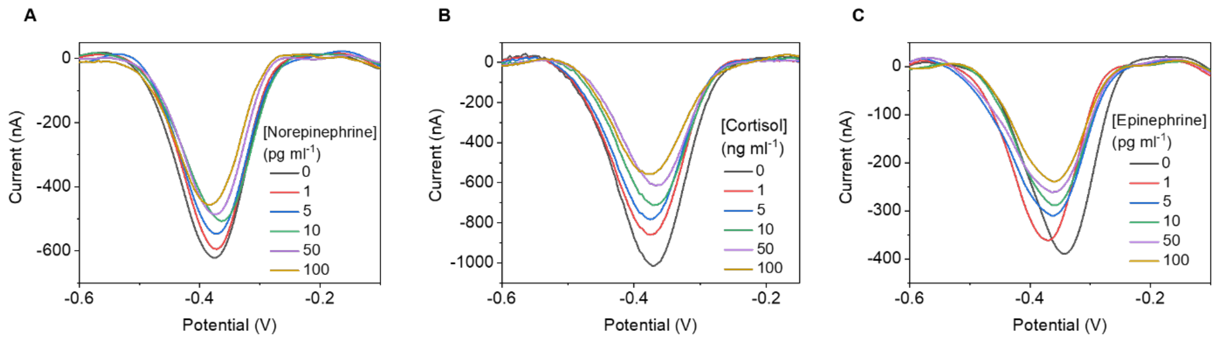

Fig. S17. SWV calibration plots of multiplexed stress immunosensors. (**A** to **C**) SWV voltammograms of norepinephrine sensors on WE1 (A), cortisol sensors on WE2 (B), and epinephrine sensors on WE3 (C). Incubation was performed by mixing standard stress hormones (prepared in 0.25× PBS, pH 7.4) with all 3 mixed redox probes (NE-MB, Cort-MB, and EPI-MB) in a 9:1 volume ratio. The mixed redox probe mixture was prepared by diluting each redox probe stock in 0.5 M phosphate buffer (pH 7.4). Detection was performed in a static chamber without flow with 5 µl of 1× PBS (pH 7.4) at 25°C.

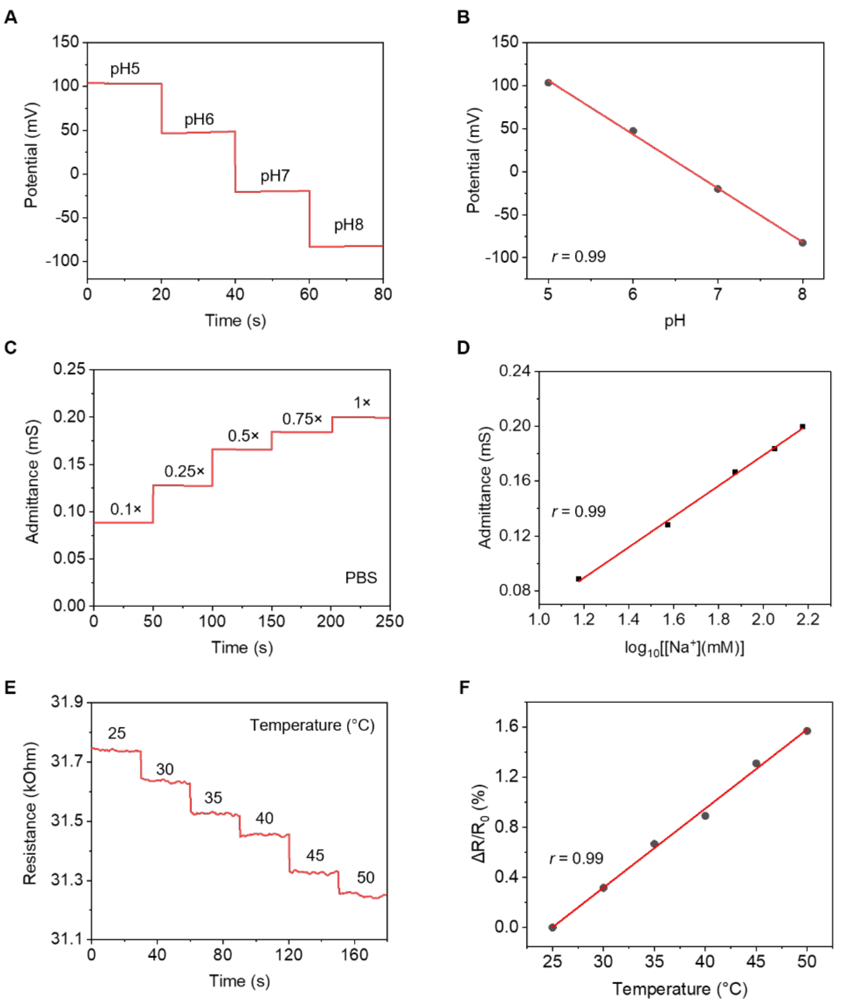

Fig. S18. Calibration of pH, ionic strength, and temperature sensors. (**A** and **B**) Potentiometric responses (A) and corresponding calibration plot (B) of the pH sensor in McIlvaine buffer. (**C** and **D**) Admittance responses (C) and corresponding calibration plot (D) of the impedimetric ionic strength sensor in PBS. (**E** and **F**) Resistance responses (E) and corresponding calibration plot (F) of the temperature sensor.

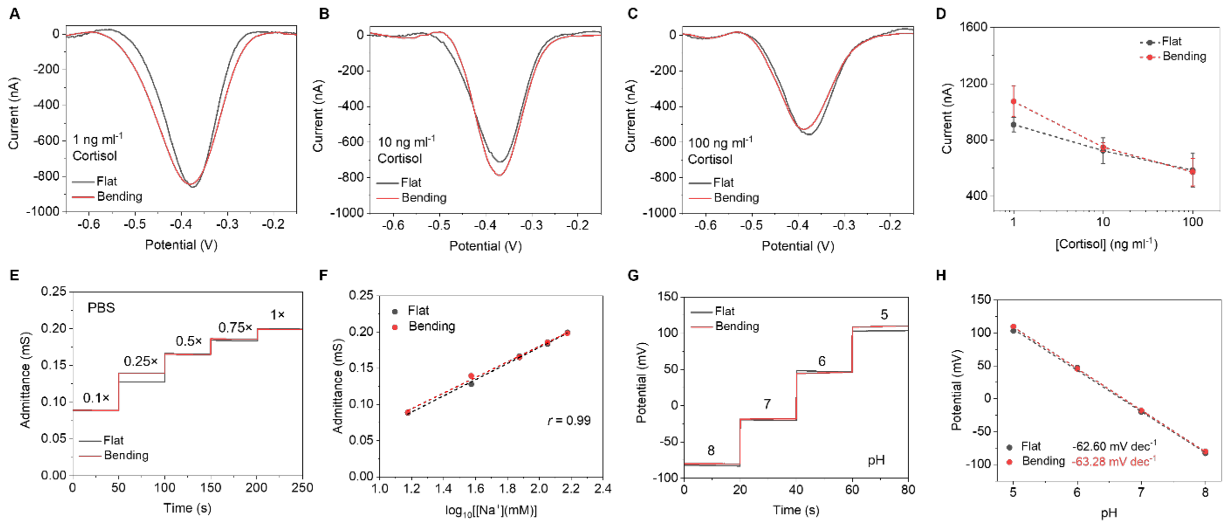

Fig. S19. Performance of the multiplexed stress immunosensor, ionic strength sensor, and pH sensor under mechanical deformation. (**A** to **C**) SWV voltammograms of the multiplexed cortisol sensors on WE2 at 1 ng ml^{-1} (A), 10 ng ml^{-1} (B), and 100 ng ml^{-1} (C) cortisol under flat and bent state. (**D**) Corresponding calibration curves of cortisol under flat and bending states. Incubation was performed by mixing standard stress hormones (prepared in 0.25× PBS, pH 7.4) with all 3 mixed redox probes (NE-MB, Cort-MB, and EPI-MB) in a 9:1 volume ratio. The mixed redox probe mixture was prepared by diluting each redox probe stock in 0.5 M phosphate buffer (pH 7.4). Detection was performed in a static chamber without flow with 5 µl of 1× PBS (pH 7.4) at 25°C. Error bars represent s.d. of the mean from 3 sensors. (**E** and **F**) Admittance responses (E) and corresponding calibration plots (F) of the impedimetric ionic strength sensor in PBS under flat and bending condition. (**G** and **H**) Potentiometric responses (G) and corresponding calibration plots (H) of pH sensors in McIlvaine buffer under flat and bending states. Bending radius, 1.5 cm.

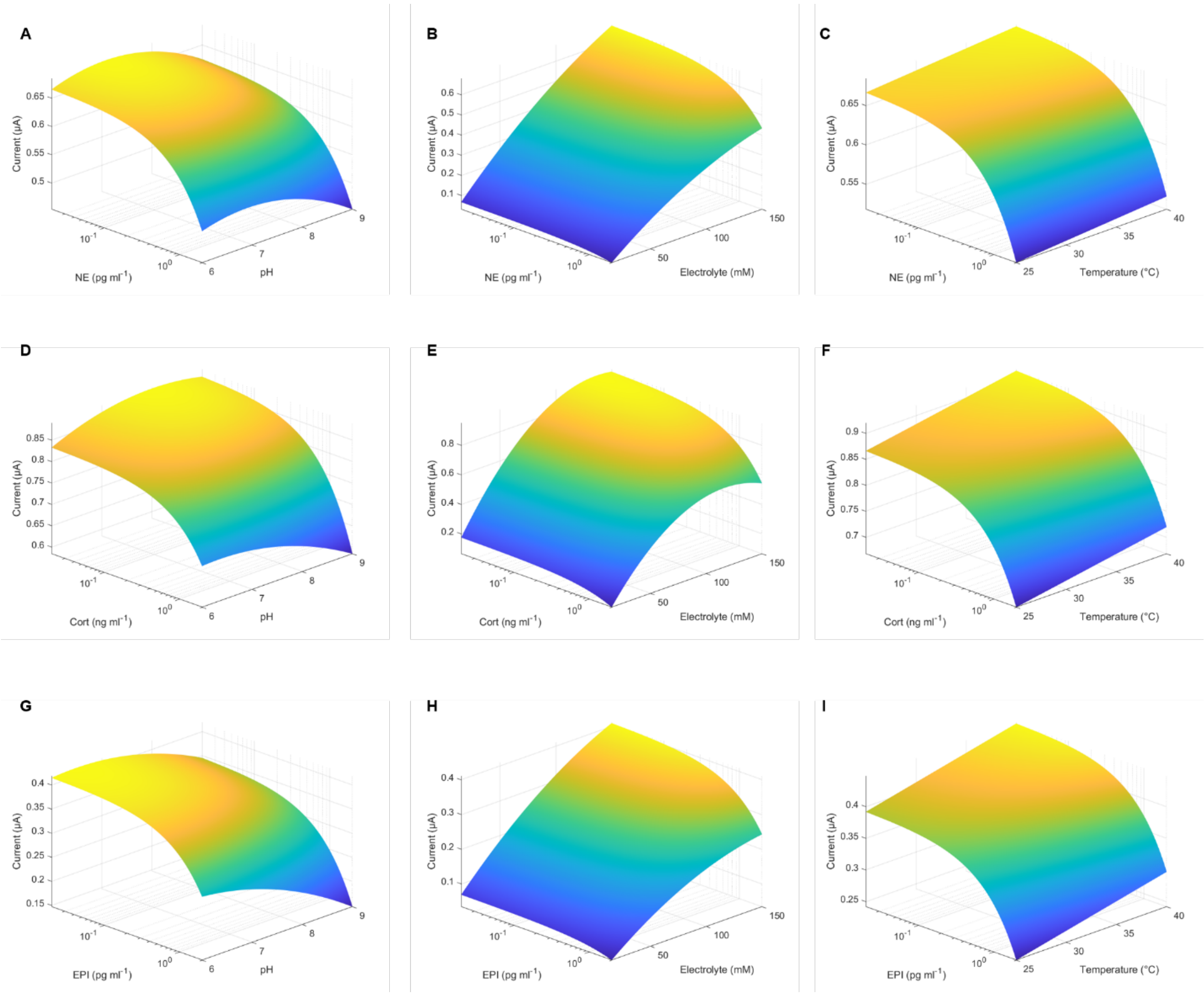

Fig. S20. Effect of pH, ionic strength, and temperature on stress immunosensor performance. (**A** to **C**) Color maps showing the dependence of norepinephrine sensors (WE1) on varying pH (A), electrolyte (B), and temperature (C). (**D** to **F**) Color maps showing the dependence of cortisol sensors (WE2) on varying pH (D), electrolyte (E), and temperature (F). (**G** to **I**) Color maps showing the dependence of epinephrine sensors (WE3) on varying pH (G), electrolyte (H), and temperature (I).

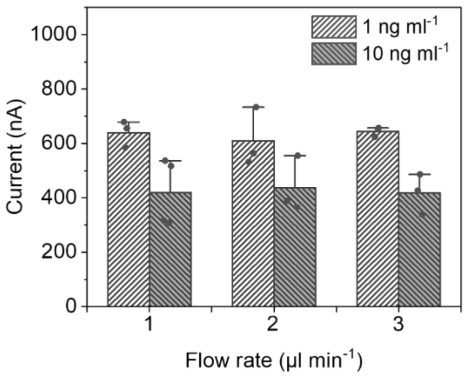

Fig. S21. Influence of flow rates on stress sensor performance. Cortisol sensor response at 1 and 10 ng ml^{-1} of cortisol under varying flow rates. Incubation was performed in 0.25× PBS (pH 7.4) with mixed redox probes (NE-MB, Cort-MB, and EPI-MB) in a 9:1 volume ratio. The mixed redox probe was prepared by diluting each redox probe stock in 0.5 M phosphate buffer (pH 7.4). Detection was performed in a static chamber without flow with 5 µl of 1× PBS (pH 7.4) at 25°C. Error bars represent the s.d. of the mean from 3 sensors.

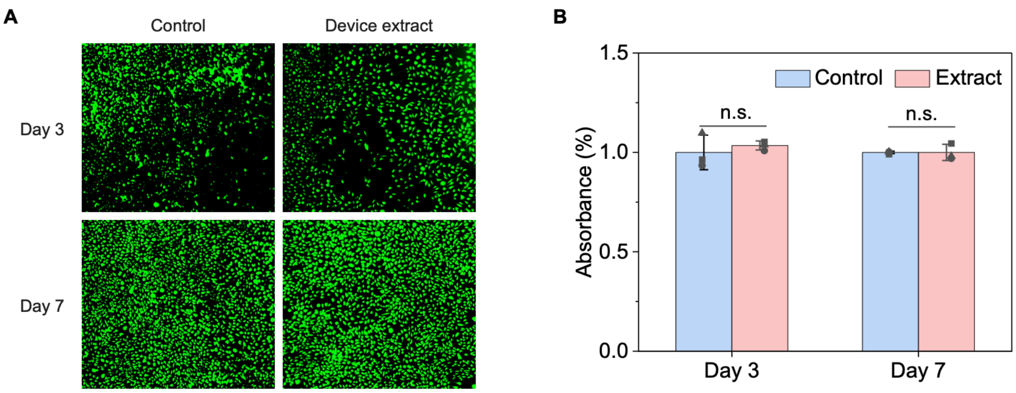

Fig. S22. In vitro cytotoxicity study. (**A**) Fluorescence microscope images of L929 cells stained with Calcein-AM (green, live cells) and Ethidium Homodimer-1 (red, dead cells) after exposure to control medium or the device extract on day 3 and day 7. (**B**) Cell viability evaluation by MTT assay. Absorbance of L929 cells exposed to control medium and device extract at day 3 and day 7. Error bars represent the s.d. of the mean from three samples. Statistical analysis was performed using Student's *t*-test. n.s., not significant.

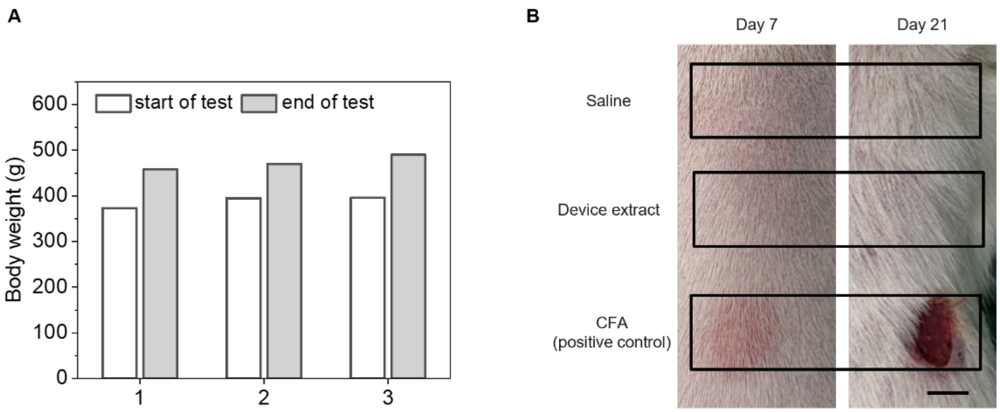

Fig. S23. Skin sensitization study. (**A**) Individual body weights of guinea pigs measured at the start and end of the skin sensitization study. (**B**) Images of the injection sites on Day 7 and Day 21 post-application. Saline and device extract sites remained normal, showing no signs of erythema, swelling, or ulceration, whereas the Complete Freund's Adjuvant (CFA) control site exhibited marked erythema and scabbing. Scale bar, 5 mm.

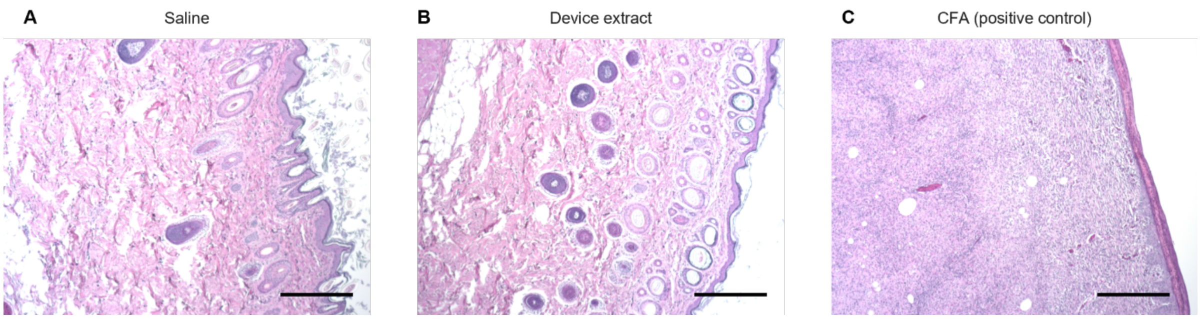

Fig. S24. In vivo biocompatibility test. Representative H&E-stained skin sections from (**A**) saline, (**B**) device extract, and (**C**) Complete Freund's Adjuvant (CFA) sites. The saline and device extract sites exhibited intact epidermal and dermal structures with no significant inflammatory cell infiltration or tissue damage, indicating good biocompatibility. In contrast, the CFA (positive control) site showed pronounced inflammatory cell infiltration and dermal disruption. Scale bars, 200 µm.

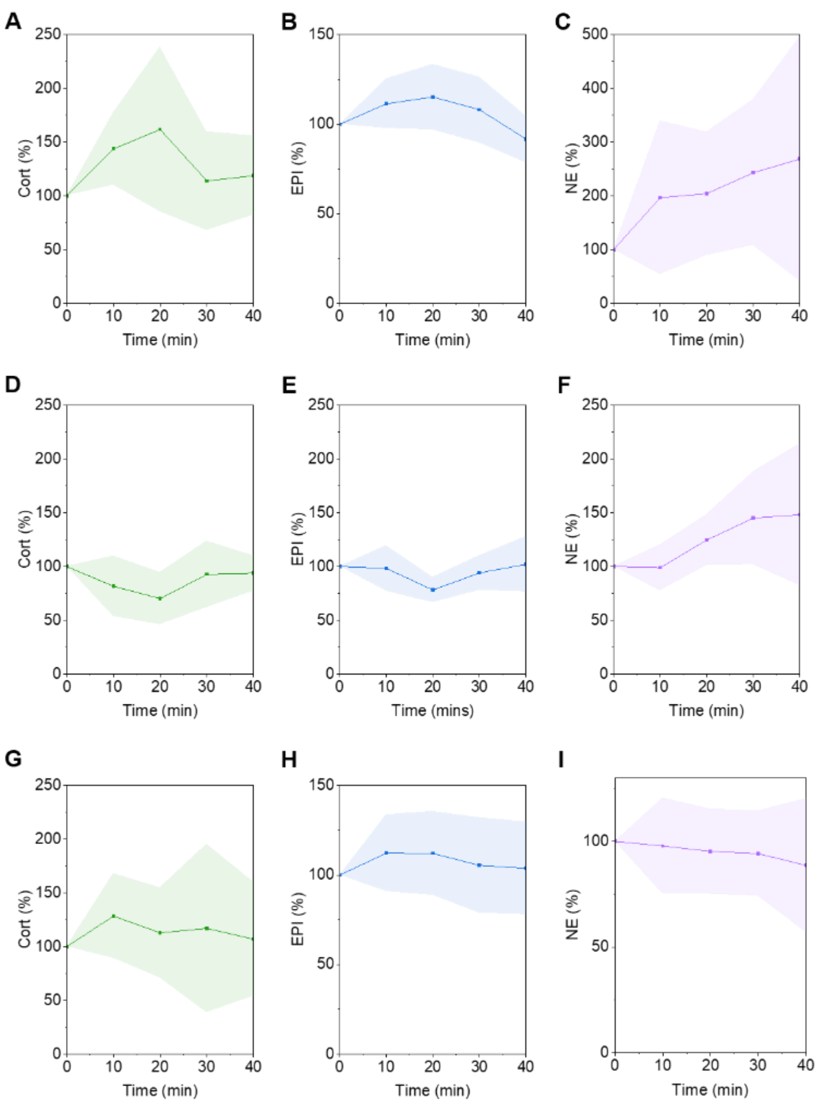

Fig. S25. Dynamic, baseline (pre-stress)-normalized changes of stress hormones. (**A** to **C**) Normalized stress hormone changes over time in cortisol (A), epinephrine (B), and norepinephrine (C) during high-intensity interval training (HIIT) study (n=10). (**D** to **F**) Normalized stress hormone changes over time cortisol (D), epinephrine (E), and norepinephrine (F) during International Affective Picture System (IAPS) (n=11). (**G** to **I**) Normalized stress hormone changes over time in cortisol (G), epinephrine (H), and norepinephrine (I) during stress mitigation through supplement intake (n=10). Shaded areas indicate the 95% confidence interval.

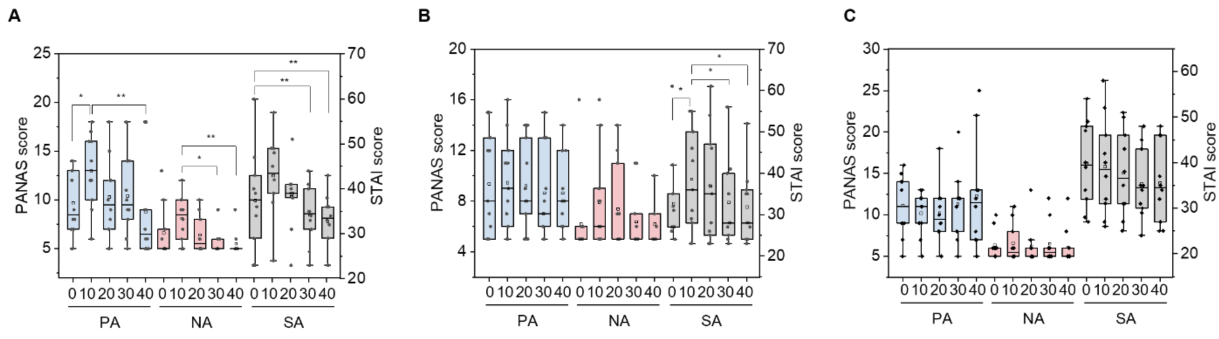

Fig. S26. Positive affect (PA), Negative affect (NA), and State Anxiety (SA) data using the Positive and Negative Affect Schedule (PANAS) and State-Trait Anxiety Inventory (STAI) surveys collected during stress modulation. One-way repeated ANOVA ($p < 0.05$) were proceeded for five time intervals, followed by Tuckey's Post Hoc tests for HIIT training (n=10) (**A**), IAPS study (n=11) (**B**), and stress releasing supplement intake (n=10) (**C**).

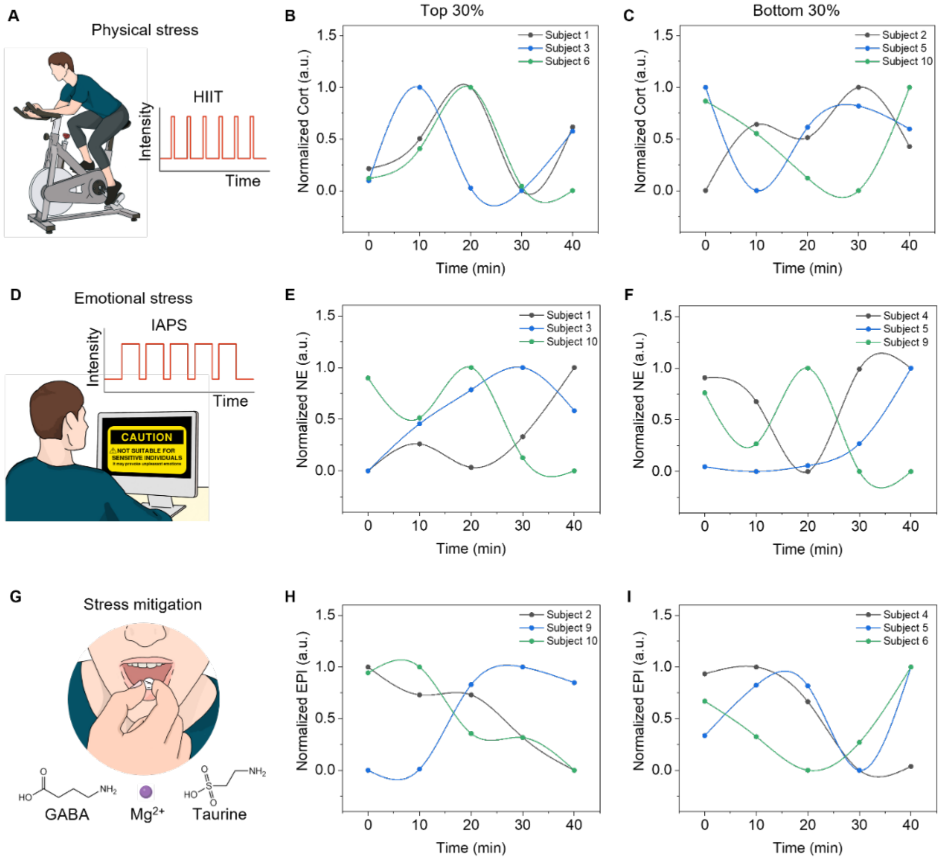

Fig. S27. Representative participants' stress hormonal profiles under various stress scenarios, stratified by a 30/70 percentile split of SA scores. (**A** to **C**) Cortisol profiles during HIIT: representative participants in the top 30% SA group (B) and bottom 30% SA group (C). (**D** to **F**) Norepinephrine profiles during IAPS: representative participants in the top 30% SA group (E) and bottom 30% SA group (F). (**G** to **I**) Epinephrine profiles during therapy: representative participants in the top 30% SA group (H) and bottom 30% SA group (I).

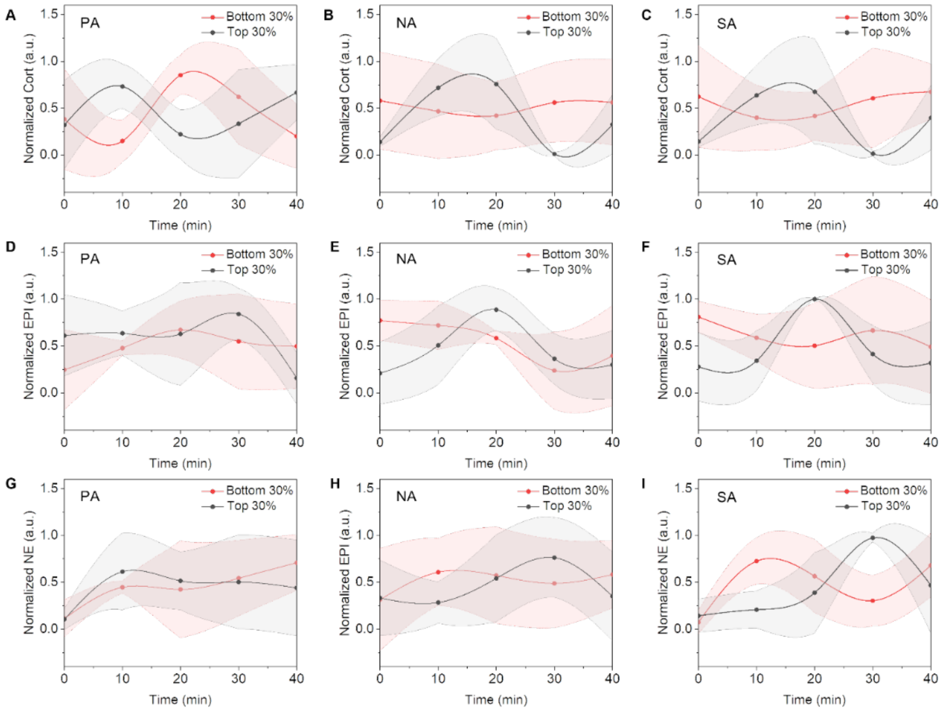

Fig. S28. Stress hormone profiles during HIIT, stratified by top and bottom 30 percentile divisions based on PANAS and SA scores. (**A** to **C**) Cortisol temporal profiles stratified by PA (A), NA (B), and SA (C). (**D** to **F**) Epinephrine temporal profiles stratified by PA (D), NA (E), and SA (F). (**G** to **I**) Norepinephrine temporal profiles stratified by PA (G), NA (H), and SA (I).

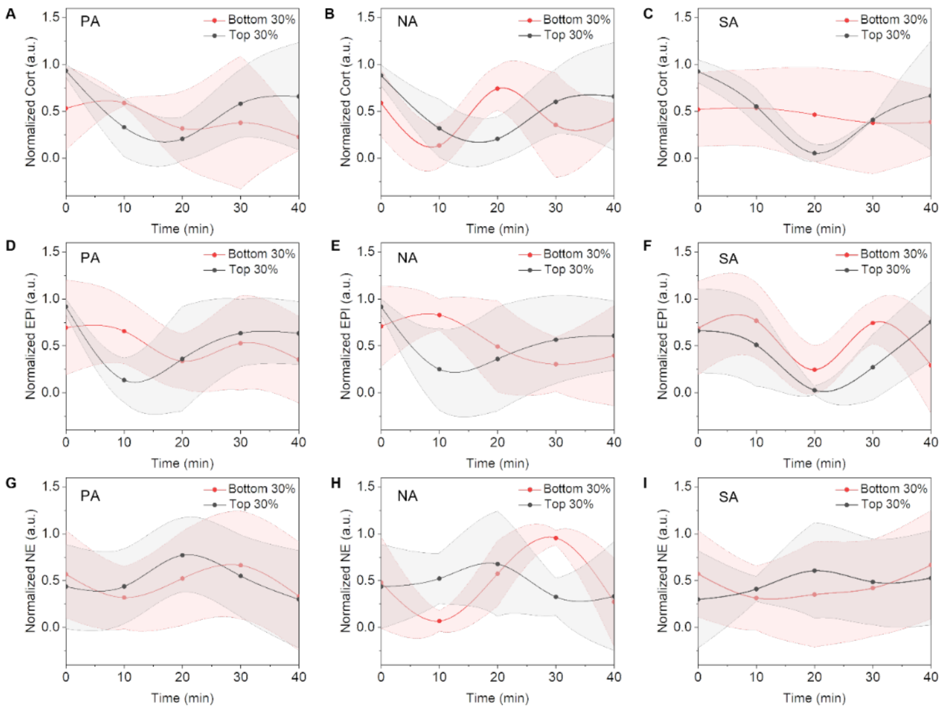

Fig. S29. Stress hormone profiles during IAPS, stratified by top and bottom 30 percentile divisions based on PANAS and SA scores. (**A** to **C**) Cortisol temporal profiles stratified by PA (A), NA (B), and SA (C). (**D** to **F**) Epinephrine temporal profiles stratified by PA (D), NA (E), and SA (F). (**G** to **I**) Norepinephrine temporal profiles stratified by PA (G), NA (H), and SA (I).

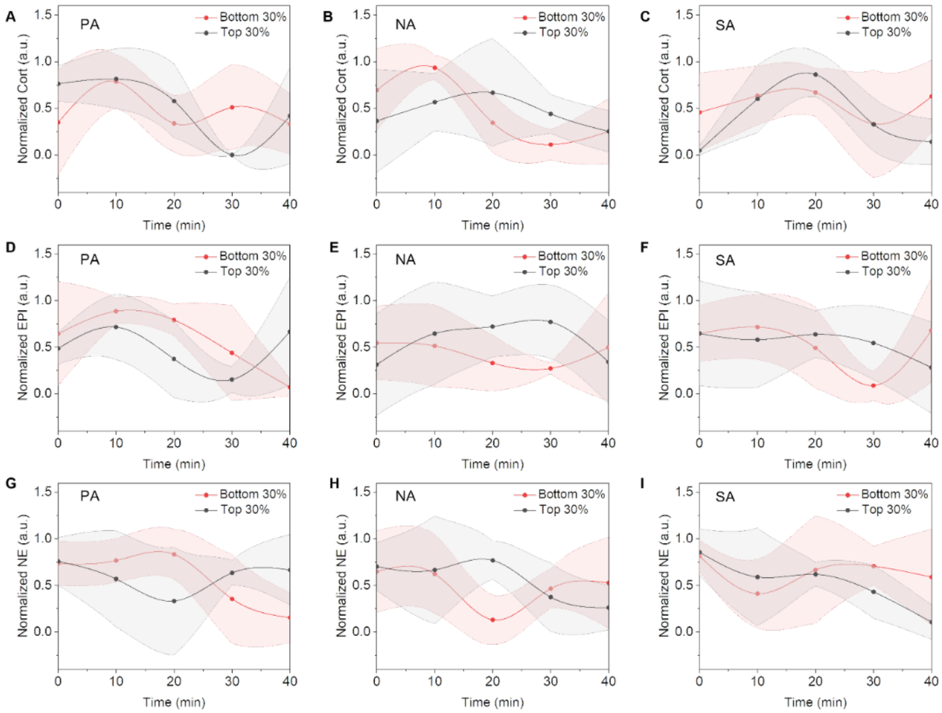

Fig. S30. Stress hormone profiles during therapy, stratified by top and bottom 30 percentile divisions based on PANAS and SA scores. (**A** to **C**) Cortisol temporal profiles stratified by PA (A), NA (B), and SA (C). (**D** to **F**) Epinephrine temporal profiles stratified by PA (D), NA (E), and SA (F). (**G** to **I**) Norepinephrine temporal profiles stratified by PA (G), NA (H), and SA (I).

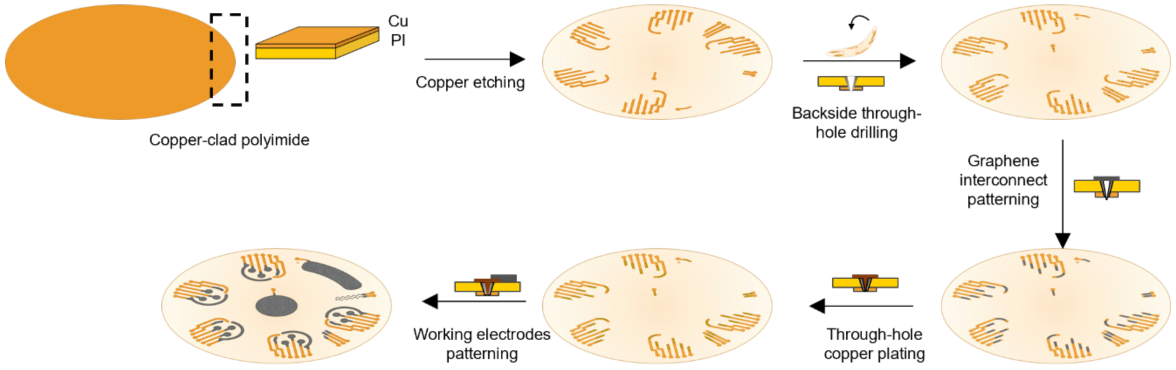

Fig. S31. Fabrication process of the double-sided flexible sensor patch. The process begins with copper-clad polyimide (Cu-PI), followed by UV laser etching of copper to define the circuit pattern. Backside through-hole drilling creates vias for interconnections between the layers. Graphene interconnects are patterned to connect the electrodes, followed by copper-plating to ensure robust electrical connectivity. The working electrodes are patterned on the backside, completing the fully integrated, double-sided flexible sensor array.

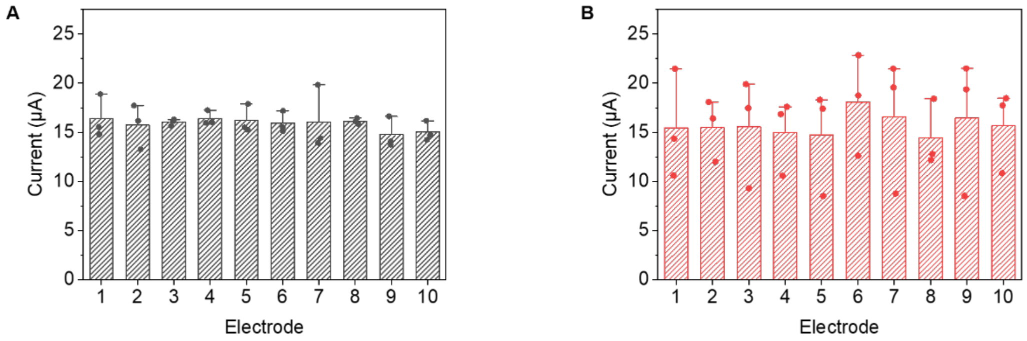

Fig. S32. Batch-to-batch variation of LEG electrodes fabricated using the double-sided processing protocol. (**A** and **B**) Oxidative peak height of single-sided (A) and double-sided (B) multiplexed LEG electrodes measured by cyclic voltammograms in 0.1 M KCl containing 5 mM $[Fe(CN)_6]^{3-}$. Error bars represent the s.d. of the mean from 3 electrodes.

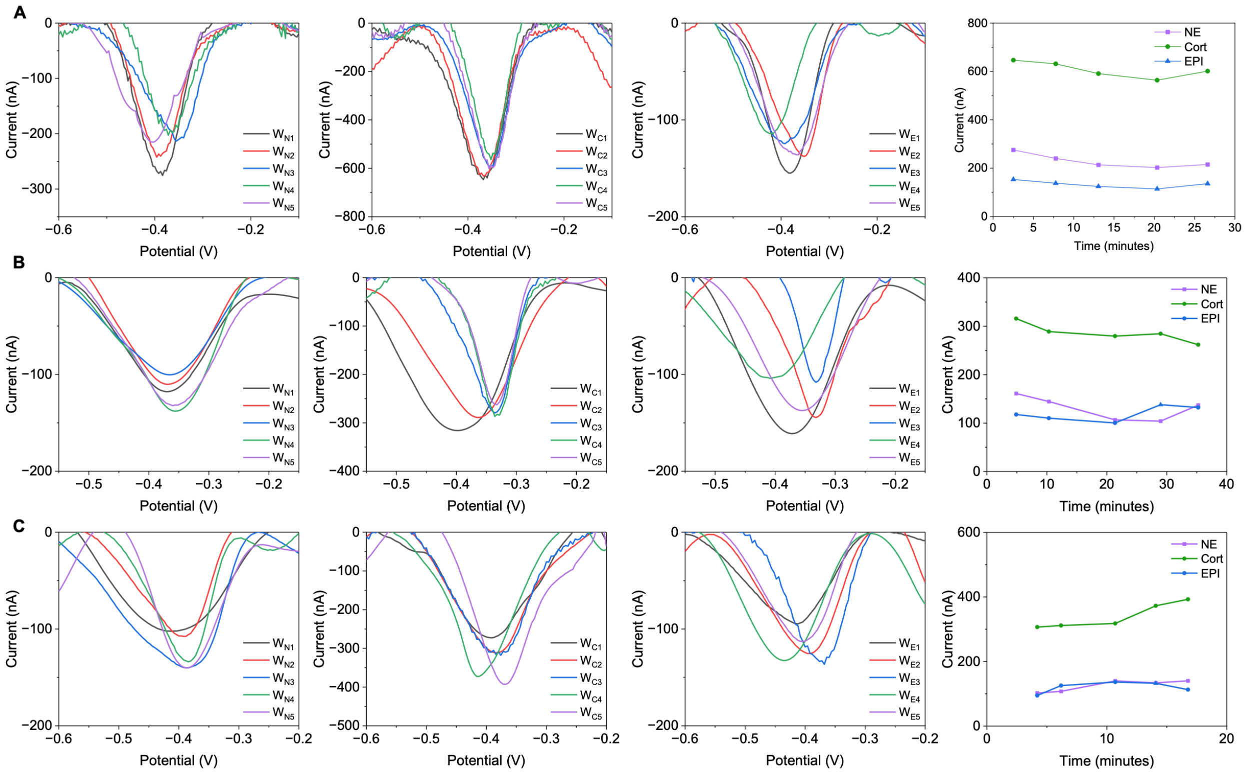

Fig. S33. On-body evaluation of the wearable sensor under various stress conditions. (**A** to **C**) Sensor response during physical stress induced by high-intensity interval training (A), under emotional stress (B), and following the intakes of stress-relief supplements (C).

Table S1. Current stress monitoring technologies.

Sensing probe	Detection method	Bio-sample	Target	Sensing range	Limit of detection (LOD)	Response/incubation time	Ref
Antibody	SWV	Sweat	Cort	0–100 ng ml^{-1} (0–276 nM)	2.7 ng ml^{-1} (7.45 nM)	15 min	This work
			EPI	0–100 pg ml^{-1} (0–546 pM)	2.73 pg ml^{-1} (14.9 pM)		
			NE	0–100 pg ml^{-1} (0–591 pM)	9.14 pg ml^{-1} (54 pM)		
MWNT/EPPGE	SWV	Plasma, urine	EPI	0.5–100 nM	0.15 pM	N/A	43
			NE	0.5–100 nM	0.09 pM		
Graphene-elastomer composite	FSCV	Artificial CSF, intestinal fluid,	EPI	10–200 nM	6.6 nM	N/A	44
			NE	10–200 nM	7.2 nM		
MIP	CV, DPV	Sweat	EPI	0.56–20 nM	8.2 nM (CV), 0.60 nM (DPV)	1 min incubation, 1 min response time	45
			Cort	0.013–0.28 µM	0.042 µM (CV), 0.025 µM (DPV)		
MWNT/IL-GC	DPV	Serum	NE	0.1–30 µM	49 nM	N/A	46
Aptamer	FET	Sweat, saliva	Cort	1 pM–1 µM	1 pM	N/A	21
MOF	FET	Artificial CSF	EPI	10 pM–1 mM	1.55 nM	10 min incubation	47
			NE	10 pM–1 mM	1.66 nM		
MnCo@C nanozymes	Colorimetric	Serum	EPI	1.09–109.2 µM	0.70 µM	3 min incubation	48
Aptamer	Fluorescence	Urine	EPI	0–300 µM	0.41 µM	N/A	49
			NE	0–300 µM	0.83 µM		
18 fluorescence probes	Fluorescence	Saliva	Cort	1 pM–1 mM	1 pM	N/A	50
			EPI	1 pM–1 mM	1 pM		

Table S2. HIIT Subject Survey: Subject 1.

PANAS	Items	Experiment Time (min)				
		0	10	20	30	40
	Upset	3	2	2	2	1
	Hostile	3	2	2	2	1
	Alert	3	2	2	2	1
	Ashamed	1	1	1	1	1
	Inspired	1	1	2	2	1
	Nervous	3	2	2	2	1
	Determined	2	4	3	2	1
	Attentive	3	4	2	2	1
	Active	3	5	3	3	2
	Afraid	3	2	2	2	1
**STAI-Y2**	**Items**	**Experiment Time (min)**				
		0	10	20	30	40
	I feel calm.	1	2	2	2	3
	I feel secure.	1	2	2	2	3
	I am tense.	3	3	2	1	2
	I feel strained.	3	2	2	1	2
	I feel at ease.	2	2	1	1	2
	I feel upset.	3	2	2	1	2
	I am presently worrying over possible misfortunes.	3	2	2	2	1
	I feel satisfied.	1	2	1	2	2
	I feel frightened.	3	2	2	1	1
	I feel comfortable.	1	2	1	2	3
	I feel self-confident.	1	2	1	1	2
	I feel nervous.	3	2	2	1	1
	I am jittery.	1	1	1	1	1
	I feel indecisive.	2	2	1	1	1
	I am relaxed.	2	2	1	2	2
	I feel content.	2	2	2	2	2
	I am worried.	3	2	2	1	1
	I feel confused.	3	3	2	1	1
	I feel steady.	2	3	2	1	1
	I feel pleasant.	1	2	1	1	1

Table S3. HIIT Subject Survey: Subject 2.

PANAS	Items	Experiment Time (min)				
		0	10	20	30	40
	Upset	1	2	2	1	1
	Hostile	1	1	1	1	1
	Alert	1	3	2	1	1
	Ashamed	1	1	1	1	1
	Inspired	1	2	1	2	2
	Nervous	1	2	2	1	1
	Determined	4	1	2	4	5
	Attentive	5	2	4	4	5
	Active	3	1	3	3	5
	Afraid	1	3	2	1	1
**STAI-Y2**	**Items**	**Experiment Time (min)**				
		0	10	20	30	40
	I feel calm.	4	2	3	4	4
	I feel secure.	4	2	3	4	4
	I am tense.	1	4	2	1	1
	I feel strained.	1	4	2	1	1
	I feel at ease.	4	2	3	4	4
	I feel upset.	1	2	3	1	1
	I am presently worrying over possible misfortunes.	1	2	1	1	1
	I feel satisfied.	4	2	3	4	4
	I feel frightened.	1	3	1	1	1
	I feel comfortable.	4	1	2	3	4
	I feel self-confident.	4	2	3	4	4
	I feel nervous.	1	3	2	1	1
	I am jittery.	1	1	1	2	1
	I feel indecisive.	1	3	2	1	1
	I am relaxed.	4	1	3	4	4
	I feel content.	4	2	3	4	4
	I am worried.	1	3	2	1	1
	I feel confused.	1	2	2	1	1
	I feel steady.	4	1	3	4	4
	I feel pleasant.	4	2	2	4	4

Table S4. HIIT Subject Survey: Subject 3.

PANAS	Items	Experiment Time (min)				
		0	10	20	30	40
	Upset	2	2	2	2	2
	Hostile	1	2	2	1	1
	Alert	1	2	2	2	2
	Ashamed	2	2	2	2	2
	Inspired	3	4	4	4	4
	Nervous	3	2	2	2	2
	Determined	3	4	4	4	4
	Attentive	3	4	4	4	4
	Active	3	4	4	4	4
	Afraid	2	2	2	2	2
STAI-Y2	Items	Experiment Time (min)				
		0	10	20	30	40
	I feel calm.	3	3	3	3	3
	I feel secure.	3	3	3	3	3
	I am tense.	2	2	2	2	2
	I feel strained.	3	3	2	2	1
	I feel at ease.	3	3	3	3	3
	I feel upset.	2	2	2	2	2
	I am presently worrying over possible misfortunes.	2	2	2	2	2
	I feel satisfied.	2	3	3	3	3
	I feel frightened.	2	2	2	2	2
	I feel comfortable.	2	3	3	4	3
	I feel self-confident.	3	3	3	3	3
	I feel nervous.	3	2	2	2	2
	I am jittery.	2	2	2	2	1
	I feel indecisive.	3	2	2	2	2
	I am relaxed.	2	3	3	4	4
	I feel content.	3	3	3	3	3
	I am worried.	2	2	2	2	2
	I feel confused.	2	2	2	2	2
	I feel steady.	2	3	3	3	3
	I feel pleasant.	2	3	3	3	3

Table S5. HIIT Subject Survey: Subject 4.

PANAS	Items	Experiment Time (min)				
		0	10	20	30	40
	Upset	1	1	1	1	1
	Hostile	1	1	1	1	1
	Alert	1	1	1	1	1
	Ashamed	1	1	1	1	1
	Inspired	1	2	2	2	2
	Nervous	1	1	2	2	1
	Determined	2	2	3	3	3
	Attentive	1	3	2	2	1
	Active	3	4	2	2	2
	Afraid	1	1	1	1	1
STAI-Y2	Items	Experiment Time (min)				
		0	10	20	30	40
	I feel calm.	3	2	2	2	3
	I feel secure.	3	3	2	3	3
	I am tense.	2	3	2	1	1
	I feel strained.	1	3	2	1	1
	I feel at ease.	2	1	2	2	2
	I feel upset.	1	1	1	1	1
	I am presently worrying over possible misfortunes.	1	1	1	1	1
	I feel satisfied.	3	2	3	3	3
	I feel frightened.	1	1	1	1	1
	I feel comfortable.	3	2	3	3	3
	I feel self-confident.	2	2	3	3	3
	I feel nervous.	1	2	2	1	1
	I am jittery.	1	1	1	1	1
	I feel indecisive.	1	1	2	1	1
	I am relaxed.	2	1	2	3	3
	I feel content.	2	2	3	2	3
	I am worried.	1	1	1	1	1
	I feel confused.	1	1	1	1	1
	I feel steady.	3	2	2	2	2
	I feel pleasant.	3	2	2	3	3

Table S6. HIIT Subject Survey: Subject 5.

PANAS	Items	Experiment Time (min)				
		0	10	20	30	40
	Upset	1	1	1	1	1
	Hostile	1	1	1	1	1
	Alert	2	1	1	1	1
	Ashamed	1	1	1	1	1
	Inspired	1	1	1	1	1
	Nervous	1	1	1	1	1
	Determined	1	1	1	1	1
	Attentive	2	1	1	1	1
	Active	1	2	1	1	1
	Afraid	1	1	1	1	1
**STAI-Y2**	**Items**	**Experiment Time (min)**				
		0	10	20	30	40
	I feel calm.	4	4	4	4	4
	I feel secure.	4	4	4	4	4
	I am tense.	1	1	1	1	1
	I feel strained.	1	1	1	1	1
	I feel at ease.	4	4	4	4	4
	I feel upset.	1	1	1	1	1
	I am presently worrying over possible misfortunes.	1	1	1	1	1
	I feel satisfied.	4	4	4	4	4
	I feel frightened.	1	1	1	4	1
	I feel comfortable.	4	4	4	4	4
	I feel self-confident.	4	4	4	4	4
	I feel nervous.	1	1	1	1	1
	I am jittery.	1	1	1	1	1
	I feel indecisive.	1	1	1	1	1
	I am relaxed.	4	3	4	4	4
	I feel content.	4	4	4	4	4
	I am worried.	1	1	1	1	1
	I feel confused.	1	1	1	1	1
	I feel steady.	4	4	4	4	4
	I feel pleasant.	4	4	4	4	4

Table S7. HIIT Subject Survey: Subject 6.

PANAS	Items	Experiment Time (min)				
		0	10	20	30	40
	Upset	1	4	1	1	1
	Hostile	1	1	1	1	1
	Alert	1	3	1	1	1
	Ashamed	1	1	1	1	1
	Inspired	1	2	2	2	1
	Nervous	2	3	1	2	2
	Determined	2	1	2	1	1
	Attentive	1	2	1	1	1
	Active	2	4	1	1	1
	Afraid	1	1	1	1	1
STAI-Y2	Items	Experiment Time (min)				
		0	10	20	30	40
	I feel calm.	3	1	3	3	3
	I feel secure.	3	3	2	3	3
	I am tense.	1	3	1	2	1
	I feel strained.	1	3	1	2	1
	I feel at ease.	3	1	3	2	2
	I feel upset.	1	3	3	1	1
	I am presently worrying over possible misfortunes.	1	1	1	1	1
	I feel satisfied.	1	2	2	2	3
	I feel frightened.	1	1	1	1	1
	I feel comfortable.	3	2	3	3	2
	I feel self-confident.	1	2	2	1	1
	I feel nervous.	2	3	1	2	2
	I am jittery.	1	1	1	1	1
	I feel indecisive.	1	1	1	1	1
	I am relaxed.	3	1	3	3	3
	I feel content.	2	1	2	2	2
	I am worried.	1	1	1	1	1
	I feel confused.	1	1	1	1	1
	I feel steady.	3	2	2	3	3
	I feel pleasant.	1	3	2	2	2

Table S8. HIIT Subject Survey: Subject 7.

PANAS	Items	Experiment Time (min)				
		0	10	20	30	40
	Upset	1	2	1	1	1
	Hostile	1	1	1	1	1
	Alert	2	4	2	2	2
	Ashamed	1	1	1	1	1
	Inspired	1	1	1	1	1
	Nervous	1	1	1	1	1
	Determined	1	3	2	2	2
	Attentive	2	3	2	2	1
	Active	2	3	2	2	1
	Afraid	1	1	1	1	1
**STAI-Y2**	**Items**	**Experiment Time (min)**				
		0	10	20	30	40
	I feel calm.	2	2	3	3	3
	I feel secure.	2	2	3	3	3
	I am tense.	2	2	1	1	1
	I feel strained.	1	2	3	1	1
	I feel at ease.	2	2	3	2	3
	I feel upset.	1	1	1	1	1
	I am presently worrying over possible misfortunes.	1	1	1	1	1
	I feel satisfied.	2	3	2	2	2
	I feel frightened.	1	1	1	1	1
	I feel comfortable.	3	2	3	3	3
	I feel self-confident.	3	3	3	2	2
	I feel nervous.	1	1	1	1	1
	I am jittery.	3	2	1	1	1
	I feel indecisive.	2	1	1	1	1
	I am relaxed.	2	2	2	3	3
	I feel content.	2	3	2	3	2
	I am worried.	1	1	1	1	1
	I feel confused.	1	1	1	1	1
	I feel steady.	2	2	2	3	2
	I feel pleasant.	2	2	2	2	2

Table S9. HIIT Subject Survey: Subject 8.

PANAS	Items	Experiment Time (min)				
		0	10	20	30	40
	Upset	1	5	1	1	1
	Hostile	1	4	1	1	1
	Alert	1	3	1	2	1
	Ashamed	1	1	1	1	1
	Inspired	1	1	1	1	1
	Nervous	1	1	1	1	1
	Determined	1	1	1	1	1
	Attentive	1	1	2	3	1
	Active	1	4	2	1	1
	Afraid	1	1	1	1	1
STAI-Y2	Items	Experiment Time (min)				
		0	10	20	30	40
	I feel calm.	4	1	3	4	3
	I feel secure.	3	3	3	3	3
	I am tense.	1	3	1	1	1
	I feel strained.	1	4	3	2	1
	I feel at ease.	3	2	3	3	3
	I feel upset.	1	4	1	1	1
	I am presently worrying over possible misfortunes.	1	1	1	1	1
	I feel satisfied.	1	2	1	3	3
	I feel frightened.	1	1	1	1	1
	I feel comfortable.	3	1	2	3	3
	I feel self-confident.	1	4	1	3	3
	I feel nervous.	1	1	1	1	1
	I am jittery.	2	1	1	2	1
	I feel indecisive.	3	1	2	2	2
	I am relaxed.	4	1	2	3	3
	I feel content.	4	1	3	3	3
	I am worried.	1	1	1	1	1
	I feel confused.	2	1	1	1	1
	I feel steady.	1	1	3	3	3
	I feel pleasant.	1	1	1	3	1

Table S10. HIIT Subject Survey: Subject 9.

PANAS	Items	Experiment Time (min)				
		0	10	20	30	40
	Upset	1	1	1	1	1
	Hostile	1	2	1	1	1
	Alert	4	5	5	5	3
	Ashamed	1	1	1	1	1
	Inspired	1	1	1	1	1
	Nervous	1	3	2	1	1
	Determined	1	3	2	4	2
	Attentive	2	3	4	2	2
	Active	1	5	3	2	1
	Afraid	1	1	1	1	1
STAI-Y2	Items	Experiment Time (min)				
		0	10	20	30	40
	I feel calm.	3	2	3	3	3
	I feel secure.	4	3	3	3	3
	I am tense.	2	3	1	1	1
	I feel strained.	1	3	1	1	1
	I feel at ease.	3	2	3	3	4
	I feel upset.	1	1	1	1	1
	I am presently worrying over possible misfortunes.	1	1	1	1	1
	I feel satisfied.	2	2	2	3	3
	I feel frightened.	1	1	1	1	1
	I feel comfortable.	3	2	2	3	3
	I feel self-confident.	3	3	3	3	3
	I feel nervous.	1	1	1	1	1
	I am jittery.	2	2	1	1	1
	I feel indecisive.	2	2	1	1	1
	I am relaxed.	2	3	3	3	4
	I feel content.	3	3	3	3	3
	I am worried.	1	1	1	1	1
	I feel confused.	1	1	1	1	1
	I feel steady.	4	4	3	3	3
	I feel pleasant.	3	4	3	3	3

Table S11. HIIT Subject Survey: Subject 10.

PANAS	Items	Experiment Time (min)				
		0	10	20	30	40
	Upset	1	2	1	1	1
	Hostile	1	1	1	1	1
	Alert	2	3	1	2	1
	Ashamed	1	2	1	1	1
	Inspired	3	3	2	2	1
	Nervous	3	1	1	1	1
	Determined	3	3	1	2	1
	Attentive	3	2	2	2	2
	Active	3	5	2	1	1
	Afraid	1	1	1	1	1
**STAI-Y2**	**Items**	**Experiment Time (min)**				
		0	10	20	30	40
	I feel calm.	3	1	3	3	3
	I feel secure.	4	4	2	3	3
	I am tense.	1	2	2	1	1
	I feel strained.	1	4	3	2	1
	I feel at ease.	4	1	2	3	4
	I feel upset.	1	1	1	1	1
	I am presently worrying over possible misfortunes.	1	1	1	1	1
	I feel satisfied.	3	3	3	3	2
	I feel frightened.	1	1	1	1	1
	I feel comfortable.	3	1	3	3	3
	I feel self-confident.	3	3	3	3	3
	I feel nervous.	2	1	1	2	1
	I am jittery.	1	2	2	2	1
	I feel indecisive.	1	1	1	1	1
	I am relaxed.	2	1	2	3	4
	I feel content.	4	3	3	3	1
	I am worried.	1	1	1	1	1
	I feel confused.	1	1	1	1	1
	I feel steady.	3	3	3	3	3
	I feel pleasant.	4	3	2	3	3

Table S12. IAPS Subject Survey: Subject 1.

PANAS	Items	Experiment Time (min)				
		0	10	20	30	40
	Upset	3	2	3	2	2
	Hostile	3	2	4	3	2
	Alert	4	3	3	3	2
	Ashamed	1	1	1	1	1
	Inspired	1	1	1	1	1
	Nervous	4	2	3	3	2
	Determined	2	1	3	1	1
	Attentive	2	5	2	1	1
	Active	3	1	2	1	1
	Afraid	5	2	3	2	3
**STAI-Y2**	**Items**	**Experiment Time (min)**				
		0	10	20	30	40
	I feel calm.	1	1	1	1	2
	I feel secure.	1	1	1	1	1
	I am tense.	3	3	3	2	2
	I feel strained.	3	3	3	2	1
	I feel at ease.	2	2	2	1	1
	I feel upset.	2	2	3	2	2
	I am presently worrying over possible misfortunes.	3	2	3	2	2
	I feel satisfied.	1	1	1	1	1
	I feel frightened.	2	2	2	2	2
	I feel comfortable.	1	1	1	2	1
	I feel self-confident.	1	2	1	2	1
	I feel nervous.	3	2	3	2	2
	I am jittery.	3	1	2	1	1
	I feel indecisive.	1	1	2	2	1
	I am relaxed.	1	1	2	1	1
	I feel content.	1	1	1	1	1
	I am worried.	3	2	3	2	2
	I feel confused.	3	2	3	2	2
	I feel steady.	2	1	2	1	2
	I feel pleasant.	1	1	1	1	1

Table S13. IAPS Subject Survey: Subject 2.

PANAS	Items	Experiment Time (min)				
		0	10	20	30	40
	Upset	1	1	1	1	1
	Hostile	1	1	1	1	1
	Alert	1	1	1	1	1
	Ashamed	1	1	1	1	1
	Inspired	1	1	1	1	1
	Nervous	1	1	1	1	1
	Determined	1	1	1	1	1
	Attentive	1	2	1	1	1
	Active	1	1	1	1	1
	Afraid	1	1	1	1	1
**STAI-Y2**	**Items**	**Experiment Time (min)**				
		0	10	20	30	40
	I feel calm.	3	3	3	3	3
	I feel secure.	3	3	3	3	3
	I am tense.	1	1	1	1	1
	I feel strained.	1	1	1	1	1
	I feel at ease.	3	3	3	3	3
	I feel upset.	1	1	1	1	1
	I am presently worrying over possible misfortunes.	1	1	1	1	1
	I feel satisfied.	3	2	3	3	3
	I feel frightened.	1	1	1	1	1
	I feel comfortable.	3	3	3	3	3
	I feel self-confident.	1	1	1	1	1
	I feel nervous.	1	1	1	1	1
	I am jittery.	1	1	1	1	1
	I feel indecisive.	1	1	1	1	1
	I am relaxed.	3	3	3	3	3
	I feel content.	3	3	3	3	3
	I am worried.	1	1	1	1	1
	I feel confused.	1	1	1	1	1
	I feel steady.	3	3	3	3	3
	I feel pleasant.	1	1	1	1	1

Table S14. IAPS Subject Survey: Subject 3.

PANAS	Items	Experiment Time (min)				
		0	10	20	30	40
	Upset	1	1	1	1	1
	Hostile	1	1	1	1	1
	Alert	1	2	3	1	1
	Ashamed	1	1	1	1	1
	Inspired	1	1	1	1	2
	Nervous	1	3	2	1	1
	Determined	1	1	1	1	3
	Attentive	1	4	1	2	1
	Active	1	1	1	1	1
	Afraid	1	2	2	1	1
STAI-Y2	Items	Experiment Time (min)				
		0	10	20	30	40
	I feel calm.	4	2	2	3	3
	I feel secure.	3	1	1	3	3
	I am tense.	2	3	2	1	1
	I feel strained.	1	1	1	1	1
	I feel at ease.	4	2	3	3	3
	I feel upset.	1	1	1	1	1
	I am presently worrying over possible misfortunes.	1	1	2	1	1
	I feel satisfied.	1	1	1	1	3
	I feel frightened.	1	2	2	1	1
	I feel comfortable.	3	2	3	2	3
	I feel self-confident.	3	3	1	3	2
	I feel nervous.	1	2	2	2	1
	I am jittery.	3	3	4	3	2
	I feel indecisive.	4	4	4	4	3
	I am relaxed.	3	2	3	3	3
	I feel content.	3	3	1	2	3
	I am worried.	1	2	2	1	1
	I feel confused.	4	4	4	2	1
	I feel steady.	2	3	4	3	3
	I feel pleasant.	2	3	2	3	3

Table S15. IAPS Subject Survey: Subject 4.

PANAS	Items	Experiment Time (min)				
		0	10	20	30	40
	Upset	1	2	1	1	1
	Hostile	1	1	1	1	1
	Alert	1	2	1	1	1
	Ashamed	1	1	1	1	1
	Inspired	1	1	1	1	1
	Nervous	1	1	1	1	1
	Determined	1	1	1	1	1
	Attentive	2	2	1	1	2
	Active	1	1	1	1	1
	Afraid	1	1	1	1	1
STAI-Y2	Items	Experiment Time (min)				
		0	10	20	30	40
	I feel calm.	4	4	4	4	4
	I feel secure.	4	4	4	4	4
	I am tense.	1	1	1	1	1
	I feel strained.	1	1	1	1	1
	I feel at ease.	4	4	4	4	4
	I feel upset.	1	1	1	1	1
	I am presently worrying over possible misfortunes.	1	1	1	1	1
	I feel satisfied.	4	4	4	4	4
	I feel frightened.	1	1	1	1	1
	I feel comfortable.	4	4	4	4	4
	I feel self-confident.	4	4	4	3	4
	I feel nervous.	1	1	1	1	1
	I am jittery.	1	1	1	1	2
	I feel indecisive.	2	1	1	1	1
	I am relaxed.	4	4	4	4	4
	I feel content.	4	4	4	4	4
	I am worried.	1	1	1	1	1
	I feel confused.	1	1	1	1	1
	I feel steady.	4	4	4	4	4
	I feel pleasant.	4	4	4	4	4

Table S16. IAPS Subject Survey: Subject 5.

PANAS	Items	Experiment Time (min)				
		0	10	20	30	40
	Upset	1	1	1	1	1
	Hostile	1	1	1	1	1
	Alert	1	1	1	1	1
	Ashamed	1	1	1	1	1
	Inspired	1	1	1	4	1
	Nervous	2	1	1	1	1
	Determined	1	1	4	1	5
	Attentive	1	1	1	1	1
	Active	1	1	1	1	1
	Afraid	1	1	1	1	1
**STAI-Y2**	**Items**	**Experiment Time (min)**				
		0	10	20	30	40
	I feel calm.	4	4	4	4	4
	I feel secure.	3	4	4	4	4
	I am tense.	1	1	1	1	1
	I feel strained.	1	1	1	1	1
	I feel at ease.	4	3	4	4	4
	I feel upset.	1	1	1	1	1
	I am presently worrying over possible misfortunes.	1	1	1	1	1
	I feel satisfied.	4	4	4	4	1
	I feel frightened.	1	1	1	1	1
	I feel comfortable.	4	4	4	4	4
	I feel self-confident.	3	3	3	3	4
	I feel nervous.	1	1	1	1	1
	I am jittery.	1	1	1	1	1
	I feel indecisive.	2	3	2	2	2
	I am relaxed.	4	4	4	4	4
	I feel content.	4	4	4	4	3
	I am worried.	1	1	1	1	1
	I feel confused.	1	2	1	1	1
	I feel steady.	4	4	4	4	4
	I feel pleasant.	4	4	4	4	4

Table S17. IAPS Subject Survey: Subject 6.

PANAS	Items	Experiment Time (min)				
		0	10	20	30	40
	Upset	1	1	1	1	1
	Hostile	1	1	1	1	1
	Alert	1	3	2	1	1
	Ashamed	1	1	1	1	1
	Inspired	3	2	1	1	1
	Nervous	1	1	1	1	1
	Determined	2	1	1	1	1
	Attentive	4	4	4	3	4
	Active	2	1	1	1	1
	Afraid	1	1	1	1	1

STAI-Y2	Items	Experiment Time (min)				
		0	10	20	30	40
	I feel calm.	4	4	4	4	4
	I feel secure.	4	4	4	4	4
	I am tense.	1	2	1	1	1
	I feel strained.	1	1	1	1	1
	I feel at ease.	4	4	4	4	4
	I feel upset.	1	2	1	1	1
	I am presently worrying over possible misfortunes.	1	2	1	1	1
	I feel satisfied.	3	3	3	3	4
	I feel frightened.	1	1	1	1	1
	I feel comfortable.	4	4	4	4	4
	I feel self-confident.	3	3	3	3	3
	I feel nervous.	1	1	1	1	1
	I am jittery.	1	1	1	1	1
	I feel indecisive.	2	2	1	1	1
	I am relaxed.	4	3	4	4	4
	I feel content.	4	4	3	3	4
	I am worried.	2	1	1	1	1
	I feel confused.	1	1	1	1	1
	I feel steady.	4	4	4	4	4
	I feel pleasant.	4	3	4	3	4

Table S18. IAPS Subject Survey: Subject 7.

PANAS	Items	Experiment Time (min)				
		0	10	20	30	40
	Upset	1	2	1	1	1
	Hostile	1	4	2	1	1
	Alert	2	3	3	2	1
	Ashamed	1	3	3	2	2
	Inspired	3	3	2	3	3
	Nervous	1	4	2	2	2
	Determined	4	3	3	3	4
	Attentive	3	3	3	3	2
	Active	3	4	3	2	2
	Afraid	1	3	3	1	1
STAI-Y2	Items	Experiment Time (min)				
		0	10	20	30	40
	I feel calm.	4	2	2	3	3
	I feel secure.	4	4	4	4	4
	I am tense.	2	3	3	2	2
	I feel strained.	2	3	3	1	2
	I feel at ease.	3	2	2	3	3
	I feel upset.	1	2	1	1	1
	I am presently worrying over possible misfortunes.	1	1	1	1	1
	I feel satisfied.	4	2	2	3	3
	I feel frightened.	1	3	3	1	2
	I feel comfortable.	4	2	3	3	3
	I feel self-confident.	3	3	2	2	3
	I feel nervous.	1	3	2	2	2
	I am jittery.	1	3	2	2	1
	I feel indecisive.	1	1	2	1	1
	I am relaxed.	2	2	2	3	3
	I feel content.	3	2	3	3	3
	I am worried.	1	1	1	2	2
	I feel confused.	1	1	1	1	2
	I feel steady.	3	3	2	3	3
	I feel pleasant.	3	2	2	3	3

Table S19. IAPS Subject Survey: Subject 8.

PANAS	Items	Experiment Time (min)				
		0	10	20	30	40
	Upset	1	1	1	1	1
	Hostile	1	1	1	1	1
	Alert	4	4	4	4	3
	Ashamed	1	1	1	1	1
	Inspired	3	1	2	3	2
	Nervous	2	2	2	2	2
	Determined	3	1	2	3	2
	Attentive	3	4	4	3	3
	Active	2	2	2	2	2
	Afraid	1	3	2	1	1
**STAI-Y2**	**Items**	**Experiment Time (min)**				
		0	10	20	30	40
	I feel calm.	4	2	3	4	4
	I feel secure.	4	2	3	4	4
	I am tense.	3	3	3	1	1
	I feel strained.	1	1	1	1	1
	I feel at ease.	4	2	3	3	4
	I feel upset.	1	1	1	1	1
	I am presently worrying over possible misfortunes.	1	1	1	1	1
	I feel satisfied.	4	2	3	4	3
	I feel frightened.	1	3	2	2	1
	I feel comfortable.	4	2	3	3	3
	I feel self-confident.	3	3	3	3	3
	I feel nervous.	2	2	2	2	2
	I am jittery.	1	3	2	1	1
	I feel indecisive.	1	1	1	1	1
	I am relaxed.	4	2	3	3	3
	I feel content.	4	2	3	4	3
	I am worried.	1	1	2	1	1
	I feel confused.	1	1	1	1	1
	I feel steady.	4	3	3	4	4
	I feel pleasant.	4	3	3	3	4

Table S20. IAPS Subject Survey: Subject 9.

PANAS	Items	Experiment Time (min)				
		0	10	20	30	40
	Upset	1	2	1	1	1
	Hostile	1	1	1	1	1
	Alert	1	1	1	1	1
	Ashamed	1	1	1	1	1
	Inspired	1	1	1	1	1
	Nervous	1	1	1	1	1
	Determined	2	1	3	3	3
	Attentive	2	1	1	1	1
	Active	1	1	1	1	1
	Afraid	1	1	1	1	1

STAI-Y2	Items	Experiment Time (min)				
		0	10	20	30	40
	I feel calm.	3	2	4	4	4
	I feel secure.	4	4	4	4	4
	I am tense.	1	1	1	1	1
	I feel strained.	1	1	1	1	1
	I feel at ease.	4	3	4	4	4
	I feel upset.	1	1	1	1	1
	I am presently worrying over possible misfortunes.	2	1	1	1	1
	I feel satisfied.	3	2	4	4	4
	I feel frightened.	1	1	1	1	1
	I feel comfortable.	4	3	4	4	4
	I feel self-confident.	3	4	4	4	4
	I feel nervous.	1	1	1	1	1
	I am jittery.	1	1	1	1	1
	I feel indecisive.	1	1	1	1	1
	I am relaxed.	4	4	4	4	4
	I feel content.	3	4	4	4	4
	I am worried.	1	1	1	1	1
	I feel confused.	1	1	1	1	1
	I feel steady.	3	4	4	4	4
	I feel pleasant.	2	2	4	4	4

Table S21. IAPS Subject Survey: Subject 10.

PANAS	Items	Experiment Time (min)				
		0	10	20	30	40
	Upset	1	3	3	3	2
	Hostile	1	3	3	2	2
	Alert	1	3	2	2	2
	Ashamed	1	2	2	2	2
	Inspired	3	1	2	2	3
	Nervous	1	3	2	2	2
	Determined	4	4	4	4	4
	Attentive	2	3	2	2	2
	Active	3	3	3	3	3
	Afraid	1	3	2	2	2
**STAI-Y2**	**Items**	**Experiment Time (min)**				
		0	10	20	30	40
	I feel calm.	4	3	2	3	3
	I feel secure.	4	2	2	3	3
	I am tense.	1	3	2	2	2
	I feel strained.	1	3	2	2	2
	I feel at ease.	4	2	2	3	3
	I feel upset.	1	3	3	3	2
	I am presently worrying over possible misfortunes.	1	2	2	2	2
	I feel satisfied.	3	2	2	3	3
	I feel frightened.	1	3	3	2	2
	I feel comfortable.	4	2	2	3	3
	I feel self-confident.	3	3	3	3	3
	I feel nervous.	1	3	2	2	2
	I am jittery.	1	3	2	2	1
	I feel indecisive.	1	2	1	1	1
	I am relaxed.	3	2	3	3	3
	I feel content.	4	2	2	3	3
	I am worried.	2	3	2	2	2
	I feel confused.	1	2	1	1	1
	I feel steady.	4	2	2	3	3
	I feel pleasant.	4	1	2	2	3

Table S22. IAPS Subject Survey: Subject 11.

PANAS	Items	Experiment Time (min)				
		0	10	20	30	40
	Upset	1	1	1	1	1
	Hostile	1	1	1	1	1
	Alert	1	2	1	1	1
	Ashamed	1	1	1	1	1
	Inspired	1	1	1	2	1
	Nervous	1	1	1	1	1
	Determined	1	1	2	2	2
	Attentive	3	2	2	2	2
	Active	2	2	2	2	2
	Afraid	1	1	1	1	1

STAI-Y2	Items	Experiment Time (min)				
		0	10	20	30	40
	I feel calm.	4	4	4	4	4
	I feel secure.	4	4	4	4	4
	I am tense.	1	1	1	1	1
	I feel strained.	1	1	1	1	1
	I feel at ease.	4	4	4	4	4
	I feel upset.	1	1	1	1	1
	I am presently worrying over possible misfortunes.	1	1	1	1	1
	I feel satisfied.	3	3	3	3	3
	I feel frightened.	1	1	1	1	1
	I feel comfortable.	3	3	3	4	4
	I feel self-confident.	3	3	3	3	3
	I feel nervous.	1	1	1	1	1
	I am jittery.	1	1	1	1	1
	I feel indecisive.	1	1	1	1	1
	I am relaxed.	4	3	3	4	4
	I feel content.	3	3	3	3	3
	I am worried.	1	1	1	1	1
	I feel confused.	1	1	1	1	1
	I feel steady.	3	3	3	3	3
	I feel pleasant.	3	3	3	3	3

Table S23. Stress Modulation Subject Survey: Subject 1.

PANAS	Items	Experiment Time (min)				
		0	10	20	30	40
	Upset	1	2	2	2	1
	Hostile	1	2	1	1	1
	Alert	1	2	1	1	1
	Ashamed	1	1	1	1	1
	Inspired	2	1	2	1	1
	Nervous	1	2	2	1	1
	Determined	2	2	2	2	2
	Attentive	2	2	2	2	2
	Active	2	2	2	2	1
	Afraid	2	3	1	1	1
**STAI-Y2**	**Items**	**Experiment Time (min)**				
		0	10	20	30	40
	I feel calm.	2	1	2	3	3
	I feel secure.	2	1	2	3	3
	I am tense.	2	3	1	1	1
	I feel strained.	2	3	2	1	1
	I feel at ease.	3	1	1	3	3
	I feel upset.	1	2	1	1	1
	I am presently worrying over possible misfortunes.	1	1	1	1	1
	I feel satisfied.	2	2	2	2	2
	I feel frightened.	1	3	1	1	1
	I feel comfortable.	3	1	2	2	3
	I feel self-confident.	3	2	1	2	2
	I feel nervous.	2	3	1	1	1
	I am jittery.	2	3	2	1	1
	I feel indecisive.	1	2	1	2	1
	I am relaxed.	2	1	2	3	2
	I feel content.	2	2	2	2	2
	I am worried.	1	3	2	1	1
	I feel confused.	1	3	1	1	1
	I feel steady.	3	2	2	2	3
	I feel pleasant.	3	2	3	3	3

Table S24. Stress Modulation Subject Survey: Subject 2.

PANAS	Items	Experiment Time (min)				
		0	10	20	30	40
	Upset	1	1	1	1	1
	Hostile	1	1	1	1	1
	Alert	1	1	1	1	1
	Ashamed	1	1	1	1	1
	Inspired	1	1	1	1	1
	Nervous	1	1	1	1	1
	Determined	1	1	1	1	1
	Attentive	1	1	1	1	1
	Active	1	1	1	1	1
	Afraid	1	1	1	1	1

STAI-Y2	Items	Experiment Time (min)				
		0	10	20	30	40
	I feel calm.	1	2	2	2	1
	I feel secure.	1	1	1	1	1
	I am tense.	1	1	1	1	1
	I feel strained.	1	1	1	1	1
	I feel at ease.	1	2	2	3	2
	I feel upset.	1	1	1	1	1
	I am presently worrying over possible misfortunes.	1	1	1	1	1
	I feel satisfied.	1	1	1	1	1
	I feel frightened.	1	1	1	1	1
	I feel comfortable.	1	2	2	1	1
	I feel self-confident.	1	1	1	1	1
	I feel nervous.	1	1	1	1	1
	I am jittery.	2	2	1	1	2
	I feel indecisive.	1	1	1	1	1
	I am relaxed.	1	2	1	2	1
	I feel content.	1	1	1	1	1
	I am worried.	1	1	1	1	1
	I feel confused.	1	1	1	1	1
	I feel steady.	1	1	1	1	2
	I feel pleasant.	1	1	1	1	1

Table S25. Stress Modulation Subject Survey: Subject 3.

PANAS	Items	Experiment Time (min)				
		0	10	20	30	40
	Upset	1	1	1	1	1
	Hostile	1	1	1	1	1
	Alert	3	2	3	2	3
	Ashamed	1	1	1	1	1
	Inspired	2	3	2	3	2
	Nervous	2	2	2	2	1
	Determined	3	2	3	2	2
	Attentive	3	4	2	2	2
	Active	3	2	2	2	2
	Afraid	1	1	1	1	1
STAI-Y2	Items	Experiment Time (min)				
		0	10	20	30	40
	I feel calm.	3	3	3	4	4
	I feel secure.	3	3	3	3	3
	I am tense.	2	2	1	1	1
	I feel strained.	2	1	1	1	1
	I feel at ease.	3	3	4	4	3
	I feel upset.	1	1	1	1	1
	I am presently worrying over possible misfortunes.	2	1	2	2	2
	I feel satisfied.	2	3	3	3	3
	I feel frightened.	1	1	1	1	1
	I feel comfortable.	3	3	4	4	4
	I feel self-confident.	3	3	3	3	3
	I feel nervous.	2	2	2	1	1
	I am jittery.	3	2	2	2	2
	I feel indecisive.	1	3	1	1	1
	I am relaxed.	3	3	3	3	3
	I feel content.	3	3	3	3	4
	I am worried.	2	2	2	1	2
	I feel confused.	1	1	1	1	1
	I feel steady.	3	3	3	3	3
	I feel pleasant.	3	3	3	4	4

Table S26. Stress Modulation Subject Survey: Subject 4.

PANAS	Items	Experiment Time (min)				
		0	10	20	30	40
	Upset	1	1	1	1	1
	Hostile	1	1	1	1	1
	Alert	2	1	1	1	1
	Ashamed	1	1	1	1	1
	Inspired	2	1	1	1	1
	Nervous	2	1	1	1	1
	Determined	1	1	1	1	1
	Attentive	2	2	3	2	2
	Active	2	2	2	2	2
	Afraid	1	1	1	1	1

STAI-Y2	Items	Experiment Time (min)				
		0	10	20	30	40
	I feel calm.	4	4	4	4	4
	I feel secure.	4	4	4	4	4
	I am tense.	1	1	1	1	1
	I feel strained.	1	1	1	1	1
	I feel at ease.	4	4	4	4	4
	I feel upset.	1	1	1	1	1
	I am presently worrying over possible misfortunes.	2	2	1	1	1
	I feel satisfied.	3	3	3	3	3
	I feel frightened.	1	1	1	1	1
	I feel comfortable.	4	4	4	3	4
	I feel self-confident.	4	3	3	3	3
	I feel nervous.	2	1	1	1	1
	I am jittery.	2	1	1	1	1
	I feel indecisive.	1	1	1	1	1
	I am relaxed.	4	4	4	4	4
	I feel content.	4	4	4	4	4
	I am worried.	1	2	1	1	1
	I feel confused.	1	1	1	1	1
	I feel steady.	4	4	4	4	4
	I feel pleasant.	4	4	4	4	4

Table S27. Stress Modulation Subject Survey: Subject 5.

PANAS	Items	Experiment Time (min)				
		0	10	20	30	40
	Upset	2	1	1	1	1
	Hostile	1	1	1	1	1
	Alert	2	1	1	1	4
	Ashamed	1	1	1	1	1
	Inspired	2	3	1	4	4
	Nervous	1	1	1	2	1
	Determined	3	1	1	2	4
	Attentive	5	5	4	5	5
	Active	3	1	1	2	5
	Afraid	1	1	1	1	1
**STAI-Y2**	**Items**	**Experiment Time (min)**				
		0	10	20	30	40
	I feel calm.	4	4	4	3	3
	I feel secure.	4	4	4	1	2
	I am tense.	1	1	1	1	1
	I feel strained.	1	1	1	1	1
	I feel at ease.	4	4	4	4	4
	I feel upset.	1	1	1	1	1
	I am presently worrying over possible misfortunes.	2	1	1	1	1
	I feel satisfied.	3	3	2	4	4
	I feel frightened.	1	1	1	1	1
	I feel comfortable.	4	4	3	3	3
	I feel self-confident.	4	4	4	4	4
	I feel nervous.	1	1	1	1	1
	I am jittery.	2	1	1	1	1
	I feel indecisive.	1	3	3	3	3
	I am relaxed.	4	4	4	4	4
	I feel content.	3	4	4	4	4
	I am worried.	1	1	1	1	1
	I feel confused.	1	1	2	1	1
	I feel steady.	4	4	4	4	4
	I feel pleasant.	3	4	4	3	4

Table S28. Stress Modulation Subject Survey: Subject 6.

PANAS	Items	Experiment Time (min)				
		0	10	20	30	40
	Upset	1	1	1	1	1
	Hostile	1	1	1	1	1
	Alert	1	1	1	1	1
	Ashamed	1	1	1	1	1
	Inspired	1	1	1	2	2
	Nervous	1	1	1	1	1
	Determined	1	1	2	2	1
	Attentive	3	5	3	4	2
	Active	1	1	2	3	2
	Afraid	1	1	1	1	1
STAI-Y2	Items	Experiment Time (min)				
		0	10	20	30	40
	I feel calm.	3	4	3	2	3
	I feel secure.	3	3	3	3	3
	I am tense.	1	1	1	1	1
	I feel strained.	1	1	1	1	1
	I feel at ease.	3	4	3	2	2
	I feel upset.	1	1	1	1	1
	I am presently worrying over possible misfortunes.	1	1	1	1	1
	I feel satisfied.	3	2	3	3	2
	I feel frightened.	1	1	1	1	1
	I feel comfortable.	3	3	3	3	3
	I feel self-confident.	3	3	3	3	2
	I feel nervous.	1	1	1	1	1
	I am jittery.	1	1	1	1	1
	I feel indecisive.	1	3	1	1	1
	I am relaxed.	3	4	3	3	2
	I feel content.	2	3	2	2	2
	I am worried.	1	1	1	1	1
	I feel confused.	1	1	1	1	1
	I feel steady.	4	4	4	3	2
	I feel pleasant.	2	2	3	3	3

Table S29. Stress Modulation Subject Survey: Subject 7.

PANAS	Items	Experiment Time (min)				
		0	10	20	30	40
	Upset	1	1	1	1	1
	Hostile	1	1	1	1	1
	Alert	1	2	4	4	5
	Ashamed	1	1	1	1	1
	Inspired	3	2	4	4	5
	Nervous	1	1	1	1	1
	Determined	3	2	3	4	5
	Attentive	5	3	4	4	5
	Active	2	2	3	4	5
	Afraid	1	1	1	1	1
**STAI-Y2**	**Items**	**Experiment Time (min)**				
		0	10	20	30	40
	I feel calm.	3	3	3	3	4
	I feel secure.	3	3	3	4	4
	I am tense.	1	1	1	1	1
	I feel strained.	1	1	1	1	1
	I feel at ease.	2	2	3	3	3
	I feel upset.	1	1	1	1	1
	I am presently worrying over possible misfortunes.	1	1	1	1	1
	I feel satisfied.	3	3	3	3	4
	I feel frightened.	1	1	1	1	1
	I feel comfortable.	3	3	3	4	4
	I feel self-confident.	3	3	3	4	4
	I feel nervous.	1	1	1	1	1
	I am jittery.	1	1	1	1	1
	I feel indecisive.	1	1	1	1	1
	I am relaxed.	3	3	3	3	4
	I feel content.	3	3	3	3	4
	I am worried.	1	1	1	1	1
	I feel confused.	1	1	1	1	1
	I feel steady.	2	3	3	3	3
	I feel pleasant.	2	3	3	3	4

Table S30. Stress Modulation Subject Survey: Subject 8.

PANAS	Items	Experiment Time (min)				
		0	10	20	30	40
	Upset	2	3	1	3	2
	Hostile	1	1	1	1	2
	Alert	1	2	2	2	3
	Ashamed	2	1	1	1	1
	Inspired	2	2	2	2	1
	Nervous	3	2	1	3	2
	Determined	2	3	3	3	3
	Attentive	2	3	2	3	3
	Active	2	3	1	2	2
	Afraid	1	1	2	2	1
**STAI-Y2**	**Items**	**Experiment Time (min)**				
		0	10	20	30	40
	I feel calm.	1	3	1	2	3
	I feel secure.	2	3	2	2	2
	I am tense.	3	2	2	3	3
	I feel strained.	1	1	3	2	3
	I feel at ease.	2	2	2	2	2
	I feel upset.	3	1	3	1	2
	I am presently worrying over possible misfortunes.	2	2	2	1	2
	I feel satisfied.	2	1	2	2	1
	I feel frightened.	2	1	1	2	1
	I feel comfortable.	3	3	2	2	1
	I feel self-confident.	3	3	2	2	3
	I feel nervous.	3	2	2	1	1
	I am jittery.	2	1	1	2	2
	I feel indecisive.	1	1	1	1	1
	I am relaxed.	2	2	2	2	3
	I feel content.	1	1	1	3	2
	I am worried.	2	2	2	2	2
	I feel confused.	2	1	2	2	3
	I feel steady.	1	2	2	2	2
	I feel pleasant.	1	3	1	2	2

Table S31. Stress Modulation Subject Survey: Subject 9.

PANAS	Items	Experiment Time (min)				
		0	10	20	30	40
	Upset	2	2	3	3	3
	Hostile	2	2	2	2	2
	Alert	4	3	2	3	3
	Ashamed	1	2	2	2	2
	Inspired	3	1	2	2	3
	Nervous	3	2	3	3	3
	Determined	4	2	3	2	2
	Attentive	2	2	2	2	2
	Active	3	4	2	3	3
	Afraid	2	3	3	2	2
**STAI-Y2**	**Items**	**Experiment Time (min)**				
		0	10	20	30	40
	I feel calm.	2	2	3	3	3
	I feel secure.	2	3	2	3	3
	I am tense.	3	2	3	2	2
	I feel strained.	3	2	3	2	2
	I feel at ease.	2	3	2	2	2
	I feel upset.	2	3	3	3	3
	I am presently worrying over possible misfortunes.	1	3	2	2	2
	I feel satisfied.	3	2	3	2	3
	I feel frightened.	2	2	3	2	2
	I feel comfortable.	3	3	2	3	3
	I feel self-confident.	2	2	2	2	2
	I feel nervous.	3	3	3	2	2
	I am jittery.	1	2	2	2	2
	I feel indecisive.	1	2	2	2	2
	I am relaxed.	3	2	3	3	3
	I feel content.	2	2	3	2	2
	I am worried.	3	3	2	3	3
	I feel confused.	3	3	4	3	3
	I feel steady.	2	2	3	3	2
	I feel pleasant.	3	3	3	3	3

Table S32. Stress Modulation Subject Survey: Subject 10.

PANAS	Items	Experiment Time (min)				
		0	10	20	30	40
	Upset	1	1	1	1	1
	Hostile	1	1	1	1	1
	Alert	4	3	3	3	3
	Ashamed	1	1	1	1	1
	Inspired	1	1	1	1	2
	Nervous	2	2	1	1	2
	Determined	2	2	2	2	3
	Attentive	3	3	3	3	3
	Active	3	3	3	3	2
	Afraid	1	1	1	1	1

STAI-Y2	Items	Experiment Time (min)				
		0	10	20	30	40
	I feel calm.	3	3	3	3	3
	I feel secure.	2	2	3	3	3
	I am tense.	3	2	2	2	1
	I feel strained.	2	2	1	1	2
	I feel at ease.	3	3	3	2	3
	I feel upset.	1	1	1	1	1
	I am presently worrying over possible misfortunes.	1	1	1	1	1
	I feel satisfied.	2	2	2	3	3
	I feel frightened.	1	1	1	1	1
	I feel comfortable.	3	3	3	2	2
	I feel self-confident.	2	2	2	2	2
	I feel nervous.	3	2	2	2	2
	I am jittery.	3	2	2	1	1
	I feel indecisive.	2	1	1	1	1
	I am relaxed.	2	3	3	3	2
	I feel content.	2	3	3	3	3
	I am worried.	3	2	2	2	1
	I feel confused.	1	1	1	1	1
	I feel steady.	2	3	2	3	3
	I feel pleasant.	3	2	3	3	3

Movie Captions

Movie S1. (separate file) Laboratory flow test illustrating the sequential sampling and release in the microfluidic module.

Movie S2. (separate file) On-body flow test showing the sequential delivery and refreshment of red dye in the detection reservoir.

REFERENCES AND NOTES

1. H. Selye, *The Stress of Life* (Mcgraw Hill, rev. ed., 1978).

2. E. O. Acevedo, P. Ekkekakis, The transactional psychobiological nature of cognitive appraisal during exercise in environmentally stressful conditions. *Psychol. Sport Exerc.* **2**, 47–67 (2001).

3. M. M. Spada, A. V. Nikčević, G. B. Moneta, A. Wells, Metacognition, perceived stress, and negative emotion. *Personal. Individ. Differ.* **44**, 1172–1181 (2008).

4. A. Mariotti, The effects of chronic stress on health: New insights into the molecular mechanisms of brain-body communication. *Future Sci. OA* **1**, FSO23 (2015).

5. A. Caspi, R. M. Houts, T. E. Moffitt, L. S. Richmond-Rakerd, M. R. Hanna, H. F. Sunde, F. A. Torvik, A nationwide analysis of 350 million patient encounters reveals a high volume of mental-health conditions in primary care. *Nat. Ment. Health* **2**, 1208–1216 (2024).

6. K. M. Harris, A. E. Gaffey, J. E. Schwartz, D. S. Krantz, M. M. Burg, The Perceived Stress Scale as a measure of stress: Decomposing score variance in longitudinal behavioral medicine studies. *Ann. Behav. Med.* **57**, 846–854 (2023).

7. C. Xu, S. A. Solomon, W. Gao, Artificial intelligence-powered electronic skin. *Nat. Mach. Intell.* **5**, 1344–1355 (2023).

8. C. Xu, Y. Song, J. R. Sempionatto, S. A. Solomon, Y. Yu, H. Y. Y. Nyein, R. Y. Tay, J. Li, W. Heng, J. Min, A. Lao, T. K. Hsiai, J. A. Sumner, W. Gao, A physicochemical-sensing electronic skin for stress response monitoring. *Nat. Electron.* **7**, 168–179 (2024).

9. B. A. Hickey, T. Chalmers, P. Newton, C.-T. Lin, D. Sibbritt, C. S. McLachlan, R. Clifton-Bligh, J. Morley, S. Lal, Smart devices and wearable technologies to detect and monitor mental health conditions and stress: A systematic review. *Sensors* **21**, 3461 (2021).

10. C. Samson, A. Koh, Stress monitoring and recent advancements in wearable biosensors. *Front. Bioeng. Biotechnol.* **8**, 1037 (2020).

11. A. S. P. Jansen, X. Van Nguyen, V. Karpitskiy, T. C. Mettenleiter, A. D. Loewy, Central command neurons of the sympathetic nervous system: Basis of the fight-or-flight response. *Science* **270**, 644–646 (1995).

12. J. Min, J. Tu, C. Xu, H. Lukas, S. Shin, Y. Yang, S. A. Solomon, D. Mukasa, W. Gao, Skin-interfaced wearable sweat sensors for precision medicine. *Chem. Rev.* **123**, 5049–5138 (2023).

13. J. Heikenfeld, A. Jajack, B. Feldman, S. W. Granger, S. Gaitonde, G. Begtrup, B. A. Katchman, Accessing analytes in biofluids for peripheral biochemical monitoring. *Nat. Biotechnol.* **37**, 407–419 (2019).

14. J. Kim, A. S. Campbell, B. E.-F. de Ávila, J. Wang, Wearable biosensors for healthcare monitoring. *Nat. Biotechnol.* **37**, 389–406 (2019).

15. D. S. Yang, R. Ghaffari, J. A. Rogers, Sweat as a diagnostic biofluid. *Science* **379**, 760–761 (2023).

16. N. Brasier, J. Wang, W. Gao, J. R. Sempionatto, C. Dincer, H. C. Ates, F. Güder, S. Olenik, I. Schauwecker, D. Schaffarczyk, E. Vayena, N. Ritz, M. Weisser, S. Mtenga, R. Ghaffari, J. A. Rogers, J. Goldhahn, Applied body-fluid analysis by wearable devices. *Nature* **636**, 57–68 (2024).

17. R. M. Torrente-Rodríguez, J. Tu, Y. Yang, J. Min, M. Wang, Y. Song, Y. Yu, C. Xu, C. Ye, W. W. IsHak, W. Gao, Investigation of cortisol dynamics in human sweat using a graphene-based wireless mHealth system. *Matter* **2**, 921–937 (2020).

18. S. Kim, B. Lee, J. T. Reeder, S. H. Seo, S.-U. Lee, A. Hourlier-Fargette, J. Shin, Y. Sekine, H. Jeong, Y. S. Oh, A. J. Aranyosi, S. P. Lee, J. B. Model, G. Lee, M.-H. Seo, S. S. Kwak, S. Jo, G. Park, S. Han, I. Park, H.-I. Jung, R. Ghaffari, J. Koo, P. V. Braun, J. A. Rogers, Soft, skin-interfaced microfluidic systems with integrated immunoassays, fluorometric sensors, and impedance measurement capabilities. *Proc. Natl. Acad. Sci. U.S.A.* **117**, 27906–27915 (2020).

19. O. Parlak, S. T. Keene, A. Marais, V. F. Curto, A. Salleo, Molecularly selective nanoporous membrane-based wearable organic electrochemical device for noninvasive cortisol sensing. *Sci. Adv.* **4**, eaar2904 (2018).

20. W. Tang, L. Yin, J. R. Sempionatto, J.-M. Moon, H. Teymourian, J. Wang, Touch-based stressless cortisol sensing. *Adv. Mater.* **33**, 2008465 (2021).

21. B. Wang, C. Zhao, Z. Wang, K.-A. Yang, X. Cheng, W. Liu, W. Yu, S. Lin, Y. Zhao, K. M. Cheung, H. Lin, H. Hojaiji, P. S. Weiss, M. N. Stojanović, A. J. Tomiyama, A. M. Andrews, S. Emaminejad, Wearable aptamer-field-effect transistor sensing system for noninvasive cortisol monitoring. *Sci. Adv.* **8**, eabk0967 (2022).

22. J. Ok, S. Park, Y. H. Jung, T. Kim, Wearable and implantable cortisol-sensing electronics for stress monitoring. *Adv. Mater.* **36**, 2211595 (2024).

23. M. Frankenhaeuser, Behavior and circulating catecholamines. *Brain Res.* **31**, 241–262 (1971).

24. R. Laverty, Catecholamines: Role in health and disease. *Drugs* **16**, 418–440 (1978).

25. H. du Toit, M. Di Lorenzo, Electrodeposited highly porous gold microelectrodes for the direct electrocatalytic oxidation of aqueous glucose. *Sens. Actuators B* **192**, 725–729 (2014).

26. B. Lu, M. R. Smyth, R. O'Kennedy, Tutorial review. Oriented immobilization of antibodies and its applications in immunoassays and immunosensors. *Analyst* **121**, 29R–32R (1996).

27. J. S. Mitchell, Y. Wu, C. J. Cook, L. Main, Direct ring conjugation of catecholamines and their immunological interactions. *Bioconjug. Chem.* **18**, 268–274 (2007).

28. J. Tu, J. Min, Y. Song, C. Xu, J. Li, J. Moore, J. Hanson, E. Hu, T. Parimon, T.-Y. Wang, E. Davoodi, T.-F. Chou, P. Chen, J. J. Hsu, H. B. Rossiter, W. Gao, A wireless patch for the monitoring of C-reactive protein in sweat. *Nat. Biomed. Eng.* **7**, 1293–1306 (2023).

29. H. Cho, H.-Y. Kim, J. Y. Kang, T. S. Kim, How the capillary burst microvalve works. *J. Colloid Interface Sci.* **306**, 379–385 (2007).

30. J. Min, S. Demchyshyn, J. R. Sempionatto, Y. Song, B. Hailegnaw, C. Xu, Y. Yang, S. Solomon, C. Putz, L. E. Lehner, J. F. Schwarz, C. Schwarzinger, M. C. Scharber, E. Shirzaei Sani, M. Kaltenbrunner, W. Gao, An autonomous wearable biosensor powered by a perovskite solar cell. *Nat. Electron.* **6**, 630–641 (2023).

31. D. Watson, L. A. Clark, A. Tellegen, Development and validation of brief measures of positive and negative affect: The PANAS scales. *J. Pers. Soc. Psychol.* **54**, 1063–1070 (1988).

32. C. D. Spielberger, *State-Trait Anxiety Inventory for Adults* (Mind Garden, 1983).

33. R. M. Bracken, D. M. Linnane, S. Brooks, Plasma catecholamine and nephrine responses to brief intermittent maximal intensity exercise. *Amino Acids* **36**, 209–217 (2009).

34. N. Athanasiou, G. C. Bogdanis, G. Mastorakos, Endocrine responses of the stress system to different types of exercise. *Rev. Endocr. Metab. Disord.* **24**, 251–266 (2023).

35. P. J. Lang, M. M. Bradley, B. N. Cuthbert, "International affective picture system (IAPS): Instruction manual and affective ratings" (Tech. Rep. A-4, Center for Research in Psychophysiology, University of Florida, 1999).

36. M. Codispoti, G. Gerra, O. Montebarocci, A. Zaimovic, M. Augusta Raggi, B. Baldaro, Emotional perception and neuroendocrine changes. *Psychophysiology* **40**, 863–868 (2003).

37. M. J. A. G. Henckens, E. J. Hermans, Z. Pu, M. Joëls, G. Fernández, Stressed memories: How acute stress affects memory formation in humans. *J. Neurosci.* **29**, 10111–10119 (2009).

38. S. Schaffer, H. W. Kim, Effects and mechanisms of taurine as a therapeutic agent. *Biomol. Ther.* **26**, 225–241 (2018).

39. J. J. Caine, T. D. Geracioti, Taurine, energy drinks, and neuroendocrine effects. *Cleve. Clin. J. Med.* **83**, 895–904 (2016).

40. D. J. White, S. De Klerk, W. Woods, S. Gondalia, C. Noonan, A. B. Scholey, Anti-stress, behavioural and magnetoencephalography effects of an L-theanine-based nutrient drink: A randomised, double-blind, placebo-controlled, crossover trial. *Nutrients* **8**, 53 (2016).

41. S. M. Lundberg, S.-I. Lee, "A unified approach to interpreting model predictions," in *Advances in Neural Information Processing Systems 30* (2017).

42. P. J. Lang, M. M. Bradley, B. N. Cuthbert, *International Affective Picture System (IAPS): Affective Ratings of Pictures and Instruction Manual* (NIMH, Center for the Study of Emotion and Attention, 2005).

43. R. N. Goyal, S. Bishnoi, Simultaneous determination of epinephrine and norepinephrine in human blood plasma and urine samples using nanotubes modified edge plane pyrolytic graphite electrode. *Talanta* **84**, 78–83 (2011).

44. J. Li, Y. Liu, L. Yuan, B. Zhang, E. S. Bishop, K. Wang, J. Tang, Y.-Q. Zheng, W. Xu, S. Niu, L. Beker, T. L. Li, G. Chen, M. Diyaolu, A.-L. Thomas, V. Mottini, J. B.-H. Tok, J. C. Y. Dunn, B. Cui, S. P. Pașca, Y. Cui, A. Habtezion, X. Chen, Z. Bao, A tissue-like neurotransmitter sensor for the brain and gut. *Nature* **606**, 94–101 (2022).

45. S. M. Mugo, S. V. Robertson, W. Lu, A molecularly imprinted screen-printed carbon electrode for electrochemical epinephrine, lactate, and cortisol metabolites detection in human sweat. *Anal. Chim. Acta* **1278**, 341714 (2023).

46. M. Mazloum-Ardakani, A. Khoshroo, High sensitive sensor based on functionalized carbon nanotube/ionic liquid nanocomposite for simultaneous determination of norepinephrine and serotonin. *J. Electroanal. Chem.* **717–718**, 17–23 (2014).

47. C. Keum, S. Park, H. Kim, H. Kim, K. H. Lee, Y. Jeong, Modular conductive MOF-gated field-effect biosensor for sensitive discrimination on the small molecular scale. *Chem. Eng. J.* **456**, 141079 (2023).

48. X. Zhu, J. Tang, X. Ouyang, Y. Liao, H. Feng, J. Yu, L. Chen, Y. Lu, Y. Yi, L. Tang, Multifunctional MnCo@C yolk-shell nanozymes with smartphone platform for rapid

colorimetric analysis of total antioxidant capacity and phenolic compounds. *Biosens. Bioelectron.* **216**, 114652 (2022).

49. L. Zou, Y. Liu, J. Liu, Capture-SELEX of DNA aptamers for label-free detection of epinephrine and norepinephrine in urine. *Biosens. Bioelectron.* **279**, 117392 (2025).

50. R. Santonocito, A. Cavallaro, A. Pappalardo, R. Puglisi, A. Marano, M. Andolina, N. Tuccitto, G. Trusso Sfrazzetto, Detection of human salivary stress biomarkers using an *easy-to-use* array sensor based on fluorescent organic molecules. *Biosens. Bioelectron.* **270**, 116986 (2025).
